# The lung microvasculature promotes alveolar type 2 cell differentiation via secreted SPARCL1

**DOI:** 10.1016/j.stemcr.2025.102451

**Published:** 2025-03-20

**Authors:** Paolo Panza, Hyun-Taek Kim, Till Lautenschläger, Janett Piesker, Stefan Günther, Yousef Alayoubi, Ondine Cleaver, Mario Looso, Didier Y.R. Stainier

**Affiliations:** 1 Department of Developmental Genetics, Max Planck Institute for Heart and Lung Research, Bad Nauheim, Germany; 2 Department of Medicine V, Internal Medicine, Infectious Diseases and Infection Control, Justus-Liebig University Giessen, Giessen, Germany; 3 Scientific Service Group Microscopy, Max Planck Institute for Heart and Lung Research, Bad Nauheim, Germany; 4 Deep Sequencing Platform, Max Planck Institute for Heart and Lung Research, Bad Nauheim, Germany; 5 Bioinformatics Core Unit, Max Planck Institute for Heart and Lung Research, Bad Nauheim, Germany; 6 UT Southwestern Medical Center, Dallas, TX, USA; 7 Member of the German Center for Lung Research, DZL-UGMLC; 8 Member of the Excellence Cluster Cardio-Pulmonary Institute, CPI

**Keywords:** lung development, lung alveologenesis, vascular-epithelial crosstalk, extracellular matrix, cell differentiation, lung organoids, endothelial cells, pericytes, alveolar type 2 cells, SPARCL1

## Abstract

Lung endothelial cells (ECs) and pericytes are closely juxtaposed with the respiratory epithelium before birth and thus may have instructive roles during development. To test this hypothesis, we screened EC-secreted proteins for their ability to alter cell differentiation in alveolar organoids. We identified secreted protein acidic and rich in cysteine-like protein 1 (SPARCL1) as an extracellular matrix molecule that can promote alveolar type 2 (AT2) cell differentiation *in vitro*. SPARCL1-treated organoids display *lysozyme* upregulation and a doubling in the number of AT2 cells at the expense of intermediate progenitors. SPARCL1 also induces the upregulation of nuclear factor κB (NF-κB) target genes, and suppression of NF-κB activation in lung organoids blocked SPARCL1 effects. NF-κB activation by lipopolysaccharide (LPS) was sufficient to induce AT2 cell differentiation; however, pharmacological inhibition of the pathway alone did not prevent it. These data support a role for SPARCL1 and NF-κB in alveolar cell differentiation and suggest a potential value in targeting this signaling axis to promote alveolar maturation and regeneration.

## Highlights

• SPARCL1 promotes AT2 cell differentiation in mouse lung organoids
• SPARCL1 is expressed by endothelial cells and pericytes in embryonic mouse lungs
• Pharmacological inhibition of NF-κB signaling blocks SPARCL1 effects
• NF-κB target genes are also expressed by mature AT2 cells in the mouse lung

## Teaser

Panza, Stainier, and colleagues identify SPARCL1, secreted by endothelial cells and pericytes in late-gestation mouse lungs, to promote alveolar type 2 cell differentiation via NF-κB signaling. This finding reveals an important signaling role of blood vessel-associated cells during lung development, with the potential to stimulate lung maturation in premature infants as well as lung repair in chronic disease.

## Introduction

At late embryonic stages in the mouse (E16-E18), the branched fetal lung undergoes a dramatic morphogenetic transition into a meshwork of alveolar sacs. At the same time, alveolar epithelial progenitors differentiate into morphologically, functionally, and molecularly distinct respiratory cell types. Flat, thin, and elongated alveolar type 1 (AT1) cells cover the surface of pre-alveolar structures, while cuboidal alveolar type 2 (AT2) cells secrete surfactant.

Several models have been proposed to explain the molecular and mechanical diversification of alveolar epithelial cells (Treutlein et al., 2014; Li et al., 2018; Frank et al., 2019). However, it remains unclear how the differentiation of alveolar progenitor cells is controlled in space and time, and in particular whether and how specific interstitial cues influence the alveolar fate (Hogan, 2018).

AT2 cell differentiation is critical for surfactant production and immune protection, preventing respiratory distress at birth. Despite the physiological importance of AT2 cells, only a few extracellular molecules have proposed roles in inducing their differentiation. Mesenchymal cell-derived fibroblast growth factor (FGF) 10 acts via epithelial FGF receptor 2 (FGFR2) and drives the apical constriction of differentiating AT2 cells from the alveolar lumen (Li et al., 2018). Consistent with these data, FGF7 stimulation together with FGFR2 overexpression induces the AT2 fate in cultured E16.5 epithelial progenitors (Brownfield et al., 2022). Hepatocyte growth factor (HGF)/c-Met signaling has also been implicated in saccular morphogenesis (Yamamoto et al., 2007) and alveolar cell proliferation (Calvi et al., 2013; Kato et al., 2018). Besides these players, however, little is known about the molecular microenvironment provided by interstitial cells to drive alveolar cell differentiation.

The mature lung in both humans and mice is highly vascularized, with approximately 30% of all cells being endothelial cells (ECs) (Weibel, 1984). While the high density of blood vessels in the lung reflects its physiological role in gas exchange, it also suggests that vascular cells have an additional role(s) beyond blood circulation and gas exchange (Ramasamy et al., 2015).

In adult stages, the lung endothelium responds to injury by activating pathways for alveolar re-epithelialization, for example, by secreting MMP14 (Ding et al., 2011; Rafii et al., 2015), and thrombospondin-1 (Lee et al., 2014). During embryonic development, disrupting vascularization *ex vivo* affects the stereotypical pattern of airway branching, consistent with a perfusion-independent crosstalk between the endothelium and epithelium (Lazarus et al., 2011). During late gestation and coinciding with lung sacculation, ECs begin to acquire organ-specific heterogeneity and start expressing distinct angiocrine factors (Gomez-Salinero et al., 2021).

Here, we address the molecular contribution of ECs and pericytes to a key event in alveologenesis, namely, the differentiation of distal airway progenitor cells into mature alveolar epithelial cells. By investigating the expression landscape of microvascular ECs in the mouse lung at late gestation, we focus on 6 candidate secreted molecules. Using an organoid model of alveolar cell differentiation (Gkatzis et al., 2021), we identify secreted protein acidic and rich in cysteine-like protein 1 (SPARCL1) as a factor that promotes AT2 cell differentiation via nuclear factor κB (NF-κB) signaling. SPARCL1/NF-κB promotes the expression of a subset of immune-related transcripts that are markers of mature AT2 cells, thereby completing their differentiation.

## Results

### SPARCL1 promotes AT2 cell differentiation in lung organoids

**Figure 1:**
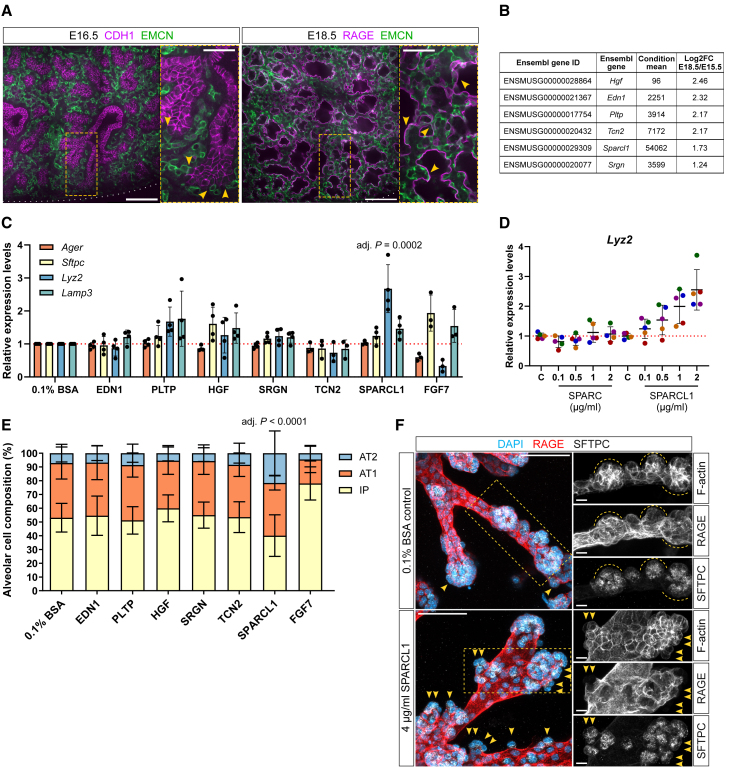
SPARCL1 promotes AT2 cell differentiation in fetal lung organoids (A) Endothelial cells (ECs) are closely juxtaposed with the alveolar epithelium in saccular lungs. Immunostaining of 150 μm precision-cut lung slices from E16.5 and E18.5 mouse embryos. CDH1 (magenta, epithelium), RAGE (magenta, basolateral membranes of AT1 cells), EMCN (green, ECs). Insets: high magnification of endothelial-epithelial contacts (yellow arrowheads). Scale bars: 100 μm, 50 μm (inset). (B) Shortlist of candidate genes encoding secreted proteins. Candidates’ expression levels at E18.5 compared with E15.5 in sorted (KDR^+^) lung ECs (Daniel et al., 2018). (C) Recombinant protein screen (*n* ≥ 3). SPARCL1 treatments lead to an upregulation of *Lyz2*. FGF7 control treatments lead to an upregulation of *Sftpc* (adj. *p* = 0.0371) and a downregulation of *Ager* and *Lyz2* (adj. *p* = 0.0002). Relative mRNA levels for markers of alveolar epithelial cell identity: *Ager* (AT1 and intermediate progenitor [IP] cells), *Sftpc* (AT2 and IP), *Lyz2* (AT2), and *Lamp3* (AT2). Data are presented as mean ± SD. *p* values are from one-way ANOVA, Tukey’s multiple comparison testing. (D) *Lyz2* mRNA levels increase dose dependently in organoids treated with SPARCL1, but not SPARC (*n* = 5 dams, at least 12 organoids per condition). Data are presented as mean ± SD. (E) Quantification of alveolar cell proportions in recombinant protein-treated organoids (*n* = 3 dams, at least 6 organoids per condition). SPARCL1 induces an increase in AT2 cell counts. FGF7 control treatments lead to an increase in IP cell counts (adj. *p* < 0.0001). Data are presented as mean ± SD. *p* values are from one-way ANOVA, Tukey’s multiple comparison testing. (F) Increased AT2 cell counts in SPARCL1-treated organoids. Maximum intensity projections of representative control and SPARCL1-treated organoids (4 μg/mL) immunostained for RAGE (red, AT1 cells) and SFTPC (white, AT2 cells). Increased numbers of SFTPC^+^/RAGE^−^ cells (yellow arrowheads) in SPARCL1-treated organoids. Insets: details of organoid branches. Scale bars: 50 μm, 10 μm (insets).

During lung sacculation (E17-P4 in mouse), a first wave of cell differentiation takes place in the distal airway. To identify extra-epithelial modulators of AT1 and AT2 cell differentiation, we focused on ECs as a potential signal-producing cell population. ECs surround the terminal airway at E16, and starting at saccular stages, they come in close proximity with the alveolar lining (Figure 1A). We analyzed a multi-organ EC expression dataset of the fetal mouse (Daniel et al., 2018) and listed genes that (1) are expressed by lung ECs, (2) encode secreted proteins, and (3) are expressed at increasing levels between E15.5 and E18.5, correlating with alveolar morphogenesis (Table S1). Shortlisted candidates include *endothelin 1* (*Edn1*), *Hgf* (both previously described as angiocrine factor genes), *phospholipid transfer protein* (*Pltp*), *transcobalamin* 2 (*Tcn2*), *Sparcl1*, and *serglycin* (*Srgn*) (Figure 1B), and their expression pattern within the distal lung interstitium was verified by *in situ* hybridization (Figure S1A).

Next, to test whether the candidate proteins could affect alveolar epithelial cell differentiation, we generated alveolar organoids from freshly isolated E14.5 distal lung epithelial tips (Gkatzis et al., 2021) and screened recombinant proteins at a dose of 1 μg/mL from days 6 to 8 of culture. During this phase, in the absence of growth factors, 10%–20% of distally located cells in the organoids acquire differentiated AT1 or AT2 cell characteristics (Gkatzis et al., 2021). To identify which proteins could alter the number of differentiated cells in organoids, we first measured the mRNA levels of the alveolar cell marker genes *Ager*, *Sftpc*, *Lyz2*, and *Lamp3* by quantitative reverse-transcription PCR (RT-qPCR) (see supplemental information for details about these markers). Next, to determine the proportion of differentiated cells in the treated organoids, we performed immunostaining for pro-surfactant protein C (Pro-SFTPC, hereafter referred to as SFTPC) and advanced glycosylation end product-specific receptor (RAGE), which can distinguish between undifferentiated intermediate progenitor (IP: SFTPC^+^/RAGE^+^) and differentiated cell types (AT1: SFTPC^−^/RAGE^+^; AT2: SFTPC^+^/RAGE^−^) in the organoids (Gkatzis et al., 2021). Control treatments using 10 ng/μL FGF7 led to increased *Sftpc* and decreased *Ager* and *Lyz2* mRNA levels (Figure 1C). At the cellular level, FGF7-treated organoids were composed mostly of IP cells at the expense of AT1 cells, an effect that can be explained by the growth-promoting activity of FGF7, or/and by its ability to induce an intermediate state in AT1 cells (Figures 1E and S1B). These data, together with previous studies (Gkatzis et al., 2021), indicate that *Sftpc* mRNA levels correlate with the number of IP cells (about 50% in control treatments) in these alveolar organoids, whereas *Ager* and *Lyz2* mRNA levels correlate with the number of AT1 and AT2 cells (40% and 10%), respectively.

Upon HGF stimulation, the branching structures of organoids appeared shorter and stumpier compared with control (Figure S1B). This morphology corresponded with an increased proportion of IP cells, which however was not statistically significant (Figures 1E and S1B). Similar to FGF7, HGF treatments also led to a slight upregulation of *Sftpc* and *Lamp3*, but no change in *Ager* or *Lyz2* mRNA levels, together reflecting the higher proportion of IP cells (Figures 1C, 1E, and S1B). Taken together, FGF7 and HGF treatments lead to the expansion of the IP cell population, likely as a result of the growth-promoting activity of these factors. The high number of cells in an intermediate state, in turn, correlates with the poor elongation of epithelial branches (Figure S1B). These data further indicate that the fetal lung organoids offer a reliable readout of alveolar cell differentiation.

Among the screened factors, we found that recombinant SPARCL1 promoted AT2-like gene expression as shown by the strong increase in *Lyz2* mRNA levels, while no change was observed in *Sftpc* or *Ager* mRNA levels (Figure 1C). This transcriptional effect was dose dependent and was not observed when organoids were treated with equal concentrations of SPARC, a protein encoded by a *Sparcl1* paralog (Figures 1D and S1C).

Organoids treated with SPARCL1 exhibited a doubling of the number of AT2 cells at the expense of IP cells, while the proportion of AT1 cells remained comparable with controls (Figure 1E). Confirming these results, high-dose SPARCL1 treatments (4 μg/mL) led to higher numbers of AT2 cells in distal branches in lung organoids, as well as increased mRNA levels for mature AT2 marker genes (Figures 1F, S1D, and S1E). Most AT2 cells in these lung organoids bud outward and maintain limited access with the lumen, similar to observations *in vivo* (Li et al., 2018). They express abundant SFTPC within cytoplasmic organelles that are predominantly apical in localization. The basolateral expression of RAGE is absent in these cells compared with neighboring ones, indicating differentiation from the intermediate cell state (Figures 1F and S1F). These cells display features of functionally mature AT2 cells, including lamellar bodies (Figure S1G), and uptake labeled phosphatidylcholine (Figure S1H) consistent with previous observations (Chiu et al., 2022).

To determine whether SPARCL1 treatments influence alveolar cell proliferation in these lung organoids, we performed EdU incorporation in the presence of recombinant SPARCL1 for 24 and 48 h. We did not observe significant differences in the number or identity of the EdU^+^ cells (Figure S2), suggesting that the increased number of AT2 cells is not due to increased proliferation.

In summary, our recombinant protein screen revealed that activation of growth factor signaling by FGF7 or HGF can inhibit organoid branch outgrowth and cell differentiation. Among the 6 secreted proteins tested, only SPARCL1 led to an increased number of mature AT2 cells, apparently by promoting their differentiation from IP cells and without affecting AT1 cell numbers.

### SPARCL1 is a marker of the E18 lung microvasculature

**Figure 2:**
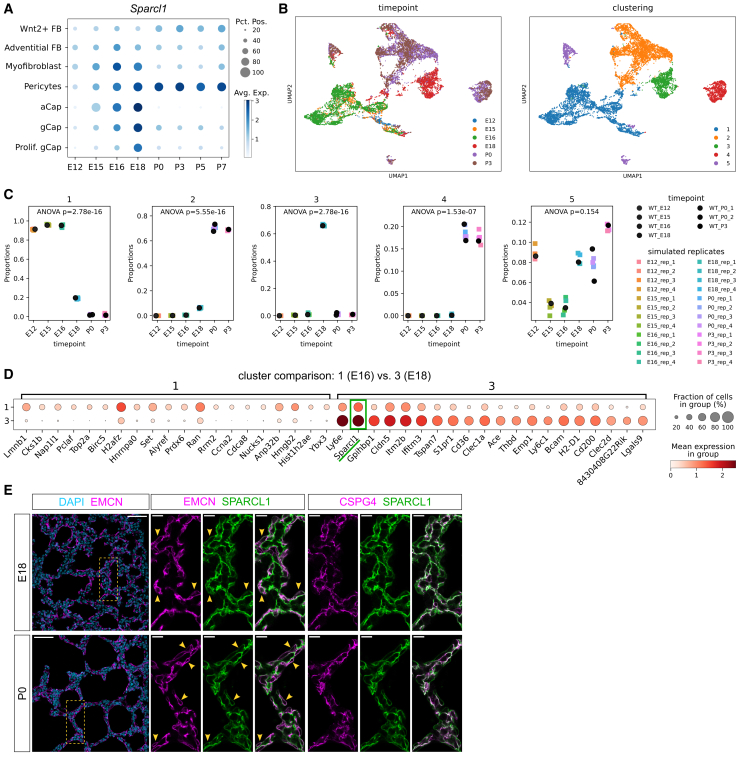
SPARCL1 is a marker of the E18 microvasculature (A) *Sparcl1* expression peaks in lung capillary ECs at E18 and is maintained in pericytes postnatally. gCap, general capillary cell; aCap, alveolar capillary cell/aerocyte; data from Negretti et al., 2021. (B) Leiden clustering of single lung EC transcriptomes from 6 developmental stages (E12-P3). Left: pre-saccular EC transcriptomes (blue, yellow, green) separate from postnatal ones (purple, brown) in the uniform manifold approximation and projection (UMAP) space. Right: cluster 3 (green) is distinct from cluster 1 (blue, pre-saccular ECs) and clusters 2 and 4 (yellow and red, postnatal ECs); data from Negretti et al., 2021. (C) Cluster 3 ECs derive from the lungs at E18. Cluster identity of ECs quantified as proportion of all ECs profiled per developmental stage. Each scRNA-seq replicate is quantified independently. A majority of ECs profiled at E12, E15, and E16 are represented in cluster 1. Clusters 2 and 4 are populated by postnatal ECs. The majority of E18 ECs are in cluster 3, and a minority in clusters 1 and 2. *p* values are from empirical Bayes moderated ANOVA test. (D) High levels of *Ly6e* and *Sparcl1* expression identify cluster 3 ECs. Top 15 differentially expressed genes between EC clusters 1 and 3. (E) SPARCL1 (green) co-localizes with membranes of microvascular ECs (EMCN, magenta) and pericytes (CSPG4, magenta) in distal lungs. Immunostaining of E18 (top row) and P0 (bottom row) lung cryosections. Left: overview of ECs in distal lung regions. Scale bars: 50 μm (left), 10 μm (middle and right).

SPARCL1, also known as Hevin, is a secreted glycoprotein that associates with the extracellular matrix (ECM) (Sullivan and Sage, 2004) and plays multiple roles in cell adhesion (Girard and Springer, 1996; Gongidi et al., 2004), synaptogenesis (Gan and Südhof, 2020; Kucukdereli et al., 2011; Singh et al., 2016), and EC quiescence (Naschberger et al., 2016; Regensburger et al., 2021). SPARCL1 is expressed in various tissues including the mammalian lung (Klingler et al., 2020). To verify the cellular source of SPARCL1 in the developing lung, we analyzed published single-cell RNA sequencing (scRNA-seq) datasets from late embryonic and early postnatal stages of lung development (Negretti et al., 2021). At E16, *Sparcl1* is broadly expressed by capillary ECs, pericytes, and myofibroblasts. By E18, *Sparcl1* is highly expressed in cell types constituting the lung microvasculature, i.e., capillary ECs and pericytes (Figure 2A). At postnatal stages, high *Sparcl1* expression is maintained in lung pericytes and is reduced in capillary ECs as well as in myofibroblasts (Figure 2A). These observations suggest that lung ECs transiently increase *Sparcl1* expression during late gestation, at a stage when AT2 cells first differentiate.

We next asked whether *Sparcl1*-expressing cells constitute a transcriptionally discrete EC subset. Leiden clustering of single-EC transcriptomes differentiated prenatal and postnatal EC groups and also highlighted a cluster predominantly composed of E18 ECs (cluster 3, Figure 2B). Analysis of the proportion of cluster identities revealed that about 60% of the profiled E18 ECs belong to cluster 3, while other stages were almost absent (Figure 2C). These data indicate that a population of E18 ECs is transcriptionally distinct from E16 and postnatal lung ECs. The comparison between the transcriptomes of clusters 1 and 3 revealed *Ly6e* and *Sparcl1* as the highest upregulated genes in cluster 3 (Figure 2D), suggesting that *Sparcl1* upregulation marks the emergence of this transitional EC subset. Cluster 3 ECs also express *Gpihbp1* (Figures 2D and S3A), a marker of general capillary (gCap) cells (Gillich et al., 2020). Among the differentially expressed genes, *Sparcl1* was the highest expressed secreted protein-encoding gene, suggesting that SPARCL1 secretion may define a signaling function of cluster 3 ECs.

Confirming these findings, immunostaining co-localized SPARCL1 with membranes of both ECs (EMCN^+^) and pericytes (CSPG4^+^, also known as NG2) in the distal lung at saccular stages (Figure 2E), including in ECs facing the alveolar surface, consistent with the aerocyte (alveolar capillary) cell population (Gillich et al., 2020). Alpha smooth muscle actin^+^ (ACTA2^+^) saccular myofibroblasts were also observed in the distal lung and accounted for a minority of SPARCL1-expressing cells (Figures S3C and S3D). Altogether, these data suggest that SPARCL1 is a marker of the late embryonic lung microvasculature, that its expression is developmentally regulated, and that it marks a transitory transcriptional state in lung capillary ECs.

### SPARCL1 activates NF-κB in alveolar epithelial cells

**Figure 3:**
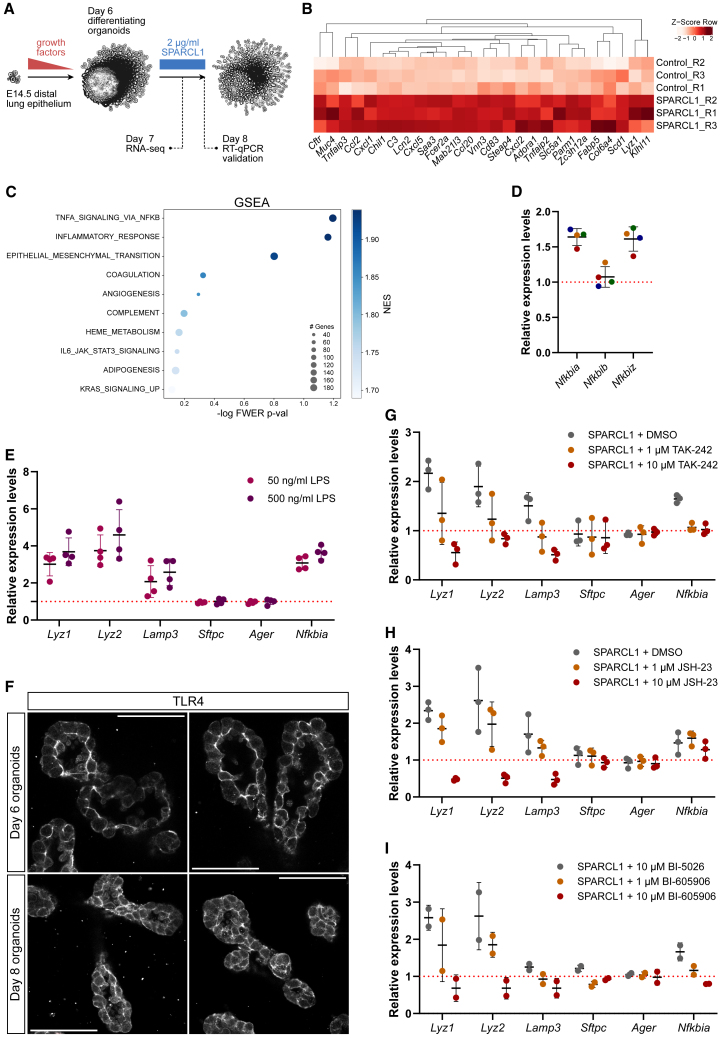
SPARCL1 triggers AT2 cell differentiation via TLR4 and NF-κB activation in lung organoids (A) Workflow schematic for RNA-seq of organoids. Control and SPARCL1-treated (2 μg/mL) organoids (*n* = 3 dams, at least 16 organoids per condition) were collected after 24 h (RNA-seq) and 48 h (RT-qPCR to validate recombinant protein activity). (B) Upregulated genes in SPARCL1-treated organoids compared with control (24 h). Heatmap showing SPARCL1-regulated genes (adj. *p* < 0.2). (C) The SPARCL1-induced gene signature is enriched in NF-κB pathway and inflammatory signaling genes. GSEA hallmark collections by high normalized enrichment score (NES): TNFA signaling via NF-κB (NES: 1.94), inflammatory response (NES: 1.94), epithelial mesenchymal transition (NES: 1.92). GSEA, gene set enrichment analysis; FWER, family-wise error rate. (D) mRNA levels for *Nfkbia* and *Nfkbiz*, but not for *Nfkbib*, are increased in SPARCL1-treated organoids (48 h, *n* = 4 dams, at least 16 organoids per condition). Data are presented as mean ± SD. (E) Bacterial LPS mimics SPARCL1 transcriptional effects in lung organoids. mRNA levels for the mature AT2 marker genes *Lyz1*, *Lyz2*, and *Lamp3*, as well as for *Nfkbia*, are increased by LPS treatment (*n* = 4 dams, at least 12 organoids per condition). *Sftpc* and *Ager* mRNA levels remained unchanged. Data are presented as mean ± SD. (F) TLR4^+^ alveolar epithelial cells in lung organoids on culture days 6 and 8, in areas of active cell differentiation. Scale bars: 50 μm. (G) Pharmacological inhibition of TLR4 blocks SPARCL1 transcriptional effects in lung organoids. 1 μM TAK-242 reduced and 10 μM TAK-242 blunted the SPARCL1-induced upregulation of *Lyz1*, *Lyz2*, and *Lamp3*, as well as of *Nfkbia* (*n* = 3 dams, at least 12 organoids per condition). *Sftpc* and *Ager* mRNA levels remained unchanged. Data are presented as mean ± SD. (H) Pharmacological inhibition of RELA nuclear translocation blocks SPARCL1 transcriptional effects in lung organoids. 10 μM JSH-23 profoundly reduced *Lyz1*, *Lyz2*, and *Lamp3* mRNA levels (*n* = 3 dams, at least 11 organoids per condition). *Sftpc* and *Ager* mRNA levels remained unchanged. *Nfkbia* expression did not change significantly upon JSH-23 treatment. Data are presented as mean ± SD. (I) Pharmacological inhibition of IKKβ blocks SPARCL1 transcriptional effects in lung organoids. 10 μM BI-605906 blunted the SPARCL1-induced upregulation of *Lyz1*, *Lyz2*, and *Lamp3*, as well as of *Nfkbia* (*n* = 2 dams, at least 12 organoids per condition). Controls were treated with the inactive and structurally similar compound BI-5026. Data are presented as mean ± SD.

To identify the molecular mechanisms leading to AT2 cell differentiation, we profiled bulk transcriptomes of organoids treated with recombinant mouse SPARCL1 (2 μg/mL) for 24 h (Figure 3A). In this experiment, recombinant SPARCL1 was biologically active, as measured by the upregulation of *Lyz1*, *Lyz2*, and *Lamp3*, but not *Sftpc* or *Ager*, after 48 h (Figure S4A). The transcriptomic comparison between SPARCL1 and control treatments did not reveal widespread differences in gene expression, as the groups did not clearly segregate by principal component analysis (Figure S4B). However, a small set of genes were significantly upregulated in SPARCL1-treated organoids, including markers of functionally mature AT2 cells (*Chil1*, *Lcn2*, *Scd1*, and *Lyz1*) (Figure 3B).

Functional enrichment by gene set enrichment analysis (Subramanian et al., 2005) showed a significant correlation with TNFA signaling via NF-κB, inflammatory response, and epithelial-mesenchymal transition (Figure 3C), suggesting that the cellular response to SPARCL1 is mediated by TNFA/NF-κB signaling. To determine the responsiveness of known NF-κB target genes to SPARCL1, we measured the expression level of *Nfkbia*, *Nfkbib*, and *Nfkbiz*, all NF-κB transcriptional targets, and all encoding IkB-family negative feedback regulators of NF-κB activity (Oeckinghaus and Ghosh, 2009). These genes are all widely expressed in the mouse lung between E16 and P0 (Figure S4C, Negretti et al., 2021). *Nfkbia* and *Nfkbiz* expression levels increase progressively in the lung at late gestation, whereas *Nfkbib* does not (Figure S4D, Beauchemin et al., 2016). Congruent with these observations, we observed an increase in *Nfkbia* and *Nfkbiz* expression levels upon SPARCL1 treatment (Figure 3D), whereas *Nfkbib* was not regulated in organoids, suggesting that SPARCL1 activates NF-κB-mediated inflammatory signaling.

To test whether activation of NF-κB signaling was sufficient to stimulate the transcription of AT2 genes in alveolar organoids, we used bacterial lipopolysaccharide (LPS) at 50 and 500 ng/mL. LPS treatments did not lead to the upregulation of the alveolar epithelial marker genes that are also expressed in IP cells, such as *Sftpc* and *Ager*. However, the expression levels of *Lyz1*, *Lyz2*, and *Lamp3* were strongly increased, as well as those of a transcriptional target of NF-κB, *Nfkbia* (Figure 3E). These data suggest that NF-κB activation induces a specific transcriptional response in alveolar epithelial cells, similar to that observed in SPARCL1-treated organoids.

The main receptor for extracellular LPS is a protein complex formed by cluster of differentiation 14 (CD14), Toll-like receptor 4 (TLR4), and lymphocyte antigen 96 (LY96, also known as MD-2). We observed TLR4 expression on the plasma membrane of the majority of alveolar cells in organoids at days 6 and 8 of culture (Figure 3F), suggesting that TLR4 may act as a SPARCL1 receptor in alveolar epithelial cells, possibly similar to its role in hepatocytes (Liu et al., 2021). To test whether SPARCL1 signals are transduced through the TLR4-dependent LPS-sensing pathway, we stimulated organoids using SPARCL1 and simultaneously pharmacologically inhibited TLR4 receptor activation using TAK-242. TAK-242 is a competitive inhibitor of TLR4 downstream signaling and disrupts the interactions between the intracellular domain of TLR4 and adapter molecules (Ii et al., 2006). Similar to previous experiments, SPARCL1 treatments led to increased levels of *Lyz1*, *Lyz2*, and *Lamp3* mRNAs. This effect was reduced by TLR4 inhibition (1 μM), and 10 μM TAK-242 abolished SPARCL1 effects. 1 and 10 μM TAK-242 also prevented the increase in *Nfkbia* mRNA levels induced by SPARCL1 (Figure 3G).

To address the role of NF-κB as a transcriptional mediator of the response to extracellular SPARCL1, we treated organoids with SPARCL1 and simultaneously inhibited the nuclear translocation of the NF-κB transcription factor subunit RELA (also known as P65) by using the small molecule JSH-23 (Shin et al., 2004). Whereas treatments using 1 μM JSH-23 only mildly reduced the SPARCL1 effect on *Lyz1*, *Lyz2*, and *Lamp3* mRNA levels, a 10 μM dose downregulated these genes to 50% of control levels (Figure 3H). However, JSH-23 treatments failed to normalize *Nfkbia* mRNA levels, suggesting a complex regulation of this pathway in epithelial cells. Altogether, these results suggest that transcription of a subset of mature AT2 cell marker genes is NF-κB dependent. This interpretation was further supported by experiments interfering with NF-κB activation using BI-605906, an IKKβ inhibitor, which led to similar results to TAK-242 and JSH-23 (Figure 3I).

Thus, we propose that SPARCL1 signal transduction in alveolar epithelial cells depends on TLR4 receptor activation and RELA nuclear translocation and transcriptional activity.

### Expression of SPARCL1/NF-κB targets marks mature AT2 cells, and NF-κB activation is sufficient for their differentiation *in vitro*

**Figure 4:**
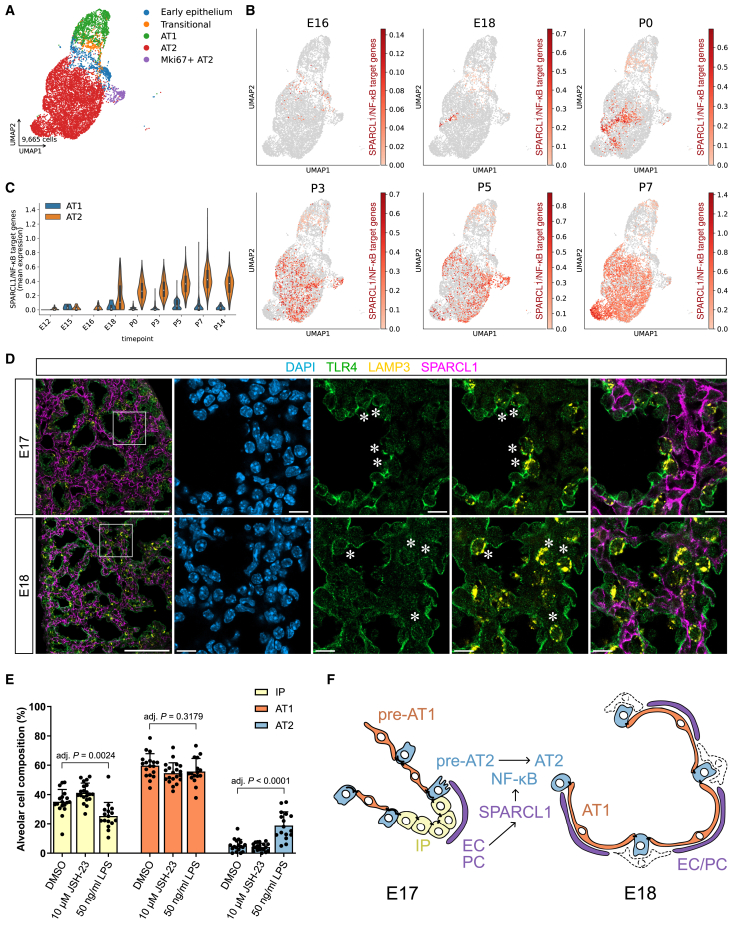
NF-κB target gene transcription correlates with AT2 cell maturity, and NF-κB activation is sufficient for AT2 cell differentiation in organoids (A) Transcriptional diversity of alveolar epithelial cells in developing mouse lungs (E12-P14). scRNA-seq data and cell type annotation from Negretti et al., 2021. Red: AT2 cells; green: AT1 cells. (B) SPARCL1/NF-κB target gene expression maps to AT2 cells beginning at E18. Mean expression levels for the SPARCL1/NF-κB target genes in alveolar epithelial cells profiled at E16-P7. (C) Stage-resolved comparison of the mean expression level for SPARCL1/NF-κB target genes between AT1 and AT2 cells. (D) TLR4 (green) is localized on the plasma membrane of distal airway epithelial cells at early saccular stages and marks the basolateral membrane of a subset of AT2 cells (asterisks; LAMP3, yellow). TLR4^+^/LAMP3^+^ cells are in contact with SPARCL1^+^ membranes (magenta). Immunostaining of E17 and E18 lung cryosections. Left: low-magnification overviews. Right: single channel and merged views. Scale bars: 100 μm (left), 10 μm (right). (E) NF-κB signaling activation in organoids is sufficient for AT2 cell differentiation. LPS stimulation of organoids increased the proportion of AT2 cells (blue bars) and decreased the number of IP cells (yellow bars). JSH-23 treatment alone did not prevent AT2 cell differentiation (*n* = 2 dams, at least 6 organoids per condition). Data are presented as mean ± SD. *p* values are from one-way ANOVA, Tukey’s multiple comparison testing. (F) Proposed signaling model. SPARCL1 is secreted by lung endothelial cells (ECs, purple) and pericytes (PCs, purple) and promotes AT2 cell differentiation (blue) via TLR4 and NF-κB. Cells marked by dashed outlines represent FGF-expressing fibroblasts.

To determine whether lung epithelial cells transcribe SPARCL1/NF-κB target genes at saccular stages, we interrogated available scRNA-seq data from the developing mouse lung (Negretti et al., 2021; Figure 4A). E16 alveolar epithelial cells only weakly express SPARCL1-responsive genes (Figure 4B, Table S2). Starting from E18, however, an increasing number of epithelial cells express this gene signature, culminating at P7 (Figures 4B and S5D). The identified cells belong to the AT2 cell cluster, and the mean expression level of the query signature of SPARCL/NF-κB targets increased in AT2 cells starting at E18, compared with low expression in AT1 cells at corresponding developmental stages (Figure 4C). These data suggest that AT2 cells express NF-κB target genes beginning at E18, correlating with high levels of vascular SPARCL1 expression. The expression of SPARCL1/NF-κB targets is maintained in AT2 cells from 15-week-old mice (Figures S5G–S5J; Hassan and Chen, 2024), suggesting that SPARCL1 might enhance AT2 cell maturation. At all stages examined, AT1 cells lowly express SPARCL1/NF-κB target genes, indicating that NF-κB activation is specific to AT2 cells.

To determine whether NF-κB pathway components are expressed in the lung epithelium, we performed immunostaining at stages when early saccular morphogenesis and alveolar cell differentiation take place. At E17 and E18, TLR4 is broadly expressed in the lung and enriched in epithelial cells. In particular, TLR4 expression marks the membrane of numerous differentiating AT2 cells (LAMP3^+^), although not exclusively. We observed basolateral expression of TLR4, in notable proximity to SPARCL1^+^ cellular membranes (Figure 4D), confirming the physical proximity of differentiating AT2 cells with the SPARCL1 expression domain, and indicate that AT2 cells have the ability to transduce NF-κB-activating extracellular signals through TLR4.

JSH-23 treatments led to a profound reduction of *Lyz1*, *Lyz2*, and *Lamp3* mRNA levels compared with controls (Figure 3H), suggesting that NF-κB inhibition might dominantly block AT2 cell differentiation in organoids. To determine whether NF-κB signaling via RELA is required for AT2 cell differentiation, we treated organoids using JSH-23 at a time point (culture day 6) when only about 2%–3% of the cells in culture are SFTPC^+^/RAGE^−^ and therefore identified as AT2 cells (Gkatzis et al., 2021). JSH-23 treatment alone did not block AT2 cell differentiation (Figure 4E). In contrast, LPS treatment of lung organoids significantly increased the proportion of AT2 cells at the expense of IP cells (Figure 4E), altogether suggesting that NF-κB activation is sufficient but not necessary for AT2 cell differentiation *in vitro*.

In summary, we identified SPARCL1 as a lung microvascular-secreted factor promoting AT2 cell differentiation in organoids. Transcriptomic and pharmacological evidence supports a role for SPARCL1-TLR4-NF-κB in the alveolar epithelium at late gestation. NF-κB-dependent transcription in alveolar cells, in turn, promotes the differentiation of AT2 cells and the acquisition of their immune physiological function.

## Discussion

A critical and unresolved question in the control of alveolar cell differentiation concerns the identity of the key signals as well as the underlying cellular and molecular mechanisms (including the crosstalk with niche cells) (Hogan, 2018). It remains unclear whether the process of alveolar cell differentiation depends on extrinsic signals besides FGFs, and organoids can help tease out intercellular communication in the lung (Gkatzis et al., 2018). Here, we provide evidence that signals from interstitial cells, and in particular ECs and pericytes, promote AT2 cell differentiation from the intermediate cell state.

In our recombinant protein screen, FGF7 treatments promoted an undifferentiated/intermediate cell state in organoids generated from E14.5 distal lung progenitor cells. In contrast, FGF7 induces AT1/AT2 cell differentiation of E16.5 progenitors (Brownfield et al., 2022). This discrepancy may arise from the different stage of tissue isolation (possibly reflecting a differential commitment status (Frank et al., 2019)), the different dose of FGF7 (10 and 50 ng/mL), and/or differential cell-non-autonomous effects from the ECM used.

Next, we identify SPARCL1 as an EC and pericyte-secreted factor that activates NF-κB in alveolar epithelial cells, in a TLR4 and RELA-dependent manner. SPARCL1-treated alveolar organoids increased the expression level of genes including *Lcn2*, *Lyz1*, *Scd1*, and *Fabp5*, all of which are markers of mature AT2 cells (Treutlein et al., 2014). Longer treatments led to upregulation of *Lyz2* and to a minor extent *Lamp3*, both also expressed in AT2 cells. These genes are involved in a set of immune functions that AT2 cells acquire before birth and maintain in the lung, including the production of surfactant and tubular myelin, and antimicrobial defense (Whitsett and Alenghat, 2015).

SPARCL1 induced AT2 cell differentiation in organoids without simultaneous FGF stimulation, suggesting that the SPARCL1 effect may be independent from an FGFR2-mediated fate selection mechanism (Brownfield et al., 2022). Together with our finding that NF-κB activation is sufficient but not necessary for AT2 cell differentiation in organoids, these observations support the co-existence of multiple pathways independently controlling alveolar cell differentiation.

In lung epithelial organoids, the early (24 h) transcriptional response to SPARCL1 was related to NF-κB signaling. These findings coincide with a described function of SPARCL1 as an extracellular NF-κB activator in the adult mouse liver (Liu et al., 2021) and lung (Zhao et al., 2024). SPARCL1 gain of function promoted inflammation in both organs. In the liver, SPARCL1 stimulated the expression of the cytokine genes *Ccl2*, *Cxcl1*, *Cxcl2*, and *Cxcl5* in hepatocytes, and, similarly, we identified these genes as SPARCL1 targets in our lung organoid model. Next, SPARCL1 co-immunoprecipitated with TLR4, suggesting that together they form a signal recognition complex at the plasma membrane (Liu et al., 2021). Consistent with this observation, we found that the epithelial response to SPARCL1 is TLR4 dependent. In adult mouse and human lungs, SPARCL1 is constitutively expressed in ECs and pericytes (Tabula Muris Consortium et al., 2018; The Tabula Sapiens Consortium, 2022), and *Sparcl1* mRNA levels increase in ECs following bleomycin administration in mice (Strunz et al., 2020). *Sparcl1* expression has been shown to increase in gCap cells upon influenza infection. SPARCL1 drives TLR4-mediated NF-κB activation in macrophages, inducing a phenotypic switch into the pro-inflammatory M1 type (Zhao et al., 2024). Beyond these data, the functional consequences of SPARCL1 activity on the lung alveolar epithelium had not been investigated.

Besides its major functions in immune cells, the NF-κB pathway has been shown to be activated in epithelial cells lining organ barriers with the external environment. In epithelial tissues, NF-κB has a conserved role in maintaining homeostasis and controlling inflammation (Pasparakis, 2012). In the epidermis, genetic data suggest a role for NF-κB in controlling epidermal cell proliferation (Seitz et al., 1998, 2000) and the propensity to differentiate (Kaufman and Fuchs, 2000; Hu et al., 2001; Pasparakis, 2012). In the gut epithelium, NF-κB activity is observed in Paneth cells, and IkBaΔN transgenic mice—in which NF-κB activity is dominantly suppressed (Schmidt-Ullrich et al., 2001)—display a reduction in LYZ2^+^ Paneth cell numbers and an increase in goblet cell numbers (Brischetto et al., 2021), supporting a role for NF-κB in intestinal epithelial cell fate establishment. However, lung phenotypes in IkBaΔN mice have not been reported.

In the human lung, alveolar pneumocytes express functional TLR2 and TLR4 (Armstrong et al., 2004), two receptors that can activate NF-κB signaling. In mouse, intra-amniotic administration of LPS at E15 led to increased AT2 cell numbers at E18 (Prince et al., 2004), an effect that was not observed in *Tlr4*^*Lps-d*^ mice, which display an attenuated response to LPS (Vogel et al., 1994). Altered numbers of AT2 cells have been identified in genetic manipulations of NF-κB signaling including the constitutive overexpression of RELA (Londhe et al., 2008) and the conditional deletion of the NF-κB-activating kinase IKKβ in lung epithelial cells (Londhe et al., 2011). These reports are aligned with our findings that NF-κB plays a direct role in AT2 cell differentiation.

We observed *Sparcl1* expression in ECs, pericytes, and myofibroblasts in the lung alveolar compartment of fetal mice. *Sparcl1* mRNA levels transiently increase in ECs between E16 and E18, consistent with reports of *Sparcl1* expression in E18.5 gCap ECs and its downregulation at postnatal stages (Zanini et al., 2023). Myofibroblasts are central players during secondary septation in the postnatal lung, but were found in low proportions in the distal region of E16-E18 lungs, supporting a primary role for microvascular ECs, pericytes, or both in alveolar cell differentiation, at least in part by secreting SPARCL1.

AT2 cell differentiation can be incomplete in the lung of very preterm neonates (Whitsett et al., 2015). Conditions of intra-amniotic infection can correlate with fetal lung damage and arrested development, as well as with increased lung maturity (Kramer et al., 2009). Similarly, the effects of manipulating NF-κB signaling in the developing lung vary (Alvira, 2014). In rhesus macaques, intra-amniotic administration of LPS at the end of the canalicular stage promoted fetal lung maturation and increased the number of AT2 cells. Transcriptomic analyses showed a negative regulation of cell proliferation and growth and an increase in genes associated with blood vessel development but also lamellar body formation (Schmidt et al., 2020). Combined with these data, our findings suggest that growth and specification of the mammalian lung vascular network is linked with AT2 cell differentiation and may be promoted by inflammatory stimuli.

In conclusion, by identifying SPARCL1 as an EC and pericyte-derived NF-κB activator promoting AT2 cell differentiation, as well as an NF-κB-dependent transcriptional program in AT2 cells, our study advances the understanding of the roles of NF-κB in lung development, with significant implications for both neonatal and chronic lung diseases.

## Methods

### Fetal lung organoid cultures

All animal care and experimental procedures in this study were approved by the local animal ethics committee at the Regierungspräsidium Darmstadt, Hessen, Germany. Isolation and culture of freshly isolated fetal distal lung epithelial tissue were carried out as described in the study by Gkatzis et al. (2021). Replicates (n) correspond to tissue isolations from different pregnant dams.

### Recombinant protein screen and chemical treatments

Purchased recombinant proteins (human EDN1: Reliatech 200-017S, mouse HGF: BioLegend 771604, human PLTP: Sino Biological 11171-H08H, mouse SPARCL1: Sino Biological 50544-M08H, human SRGN: Sino Biological 13648-H08H, mouse TCN2: Sino Biological 50693-M08H, and human FGF7: PeproTech 100-19) were diluted in sterile PBS containing 0.1% BSA (Sigma A1595). Recombinant proteins were used for screening at a concentration of 1 μg/mL in organoid medium without growth factors. Control conditions refer to treatments using isovolumes of 0.1% BSA in PBS. TAK-242/Resatorvid (Hycultec HY-11109), JSH-23 (Hycultec HY-13982), BI-605906, and BI-5026 (https://www.opnme.com/) were diluted in DMSO (Sigma D2650). LPS (Sigma L2630) was diluted in double distilled water.

### Whole-mount organoid immunostaining

Organoid immunostaining was performed according to Gkatzis et al. (2021). At least 6 organoids from 3 wells per condition were quantified. Confocal images of distal regions in immunostained and live organoids were collected on a Zeiss CellDiscoverer 7 microscope, using a 50x Plan-APOCHROMAT 1.2× NA objective and 0.5× magnification.

### Quantification of cell composition in organoids

Alveolar cell type composition was quantified according to methods described in Gkatzis et al. (2021). Cell counts are included in Data S3.

### Quantification and statistical analysis

RT-qPCR data were normalized to values from control organoids (0.1% BSA). All results are expressed as mean values ± SD. *p* values are from one-way ANOVA, Tukey’s multiple comparison testing.

## Resource availability

### Lead contact

Requests for further information and resources should be directed to and will be fulfilled by the lead contact, Paolo Panza (paolo.panza@mpi-bn.mpg.de).

### Materials availability

This study did not generate new unique reagents.

### Data and code availability

The accession number for the RNA-seq data reported in this paper is GEO: GSE279892. RNA-seq data from sorted KDR^+^ cells (Daniel et al., 2018) were kindly made available by the Cleaver lab. scRNA-seq data published by the Sucre lab (Negretti et al., 2021) were obtained from https://lungcells.app.vumc.org/ and from GEO: GSE165063. scRNA-seq data published by the Chen lab (Hassan and Chen, 2024) were obtained from GEO: GSE158192.

## Acknowledgments

We thank Jennifer Sucre for sharing the scRNA-seq datasets and for suggestions on data analysis, David Frank, Andrew Vaughan, Gan Zhao, Elisabeth Naschberger, and Michael Stürzl for sharing reagents, Lienhard Schmitz, Saverio Bellusci, and Chi-Chung Wu for discussions, Felix Gunawan and João Cardeira-da-Silva for critical reading of the manuscript, Kenny Mattonet and Radhan Ramadass for microscopy technical advice, Petra Neeb for technical help, Nouha Ritschel and the animal facility staff for excellent animal care, Petra Prückl for advice on animal welfare, and Simon Perathoner for scientific administrative support. BI-605906 and BI-5026 were kindly provided by Boehringer Ingelheim via its open innovation platform opnMe (https://www.opnme.com). This work was supported by a CPI flexible outbreak project grant awarded to P.P. and D.Y.R.S. and by funds from the 10.13039/501100004189Max Planck Society to D.Y.R.S.

## Author contributions

P.P. and D.Y.R.S. designed research; P.P., H.-T.K., T.L., J.P., and S.G. performed research; P.P., H.-T.K., T.L., J.P., S.G., Y.A., O.C., and M.L. analyzed the data; and P.P. and D.Y.R.S. wrote the paper.

## Declaration of interests

The authors declare no competing interests.

## Supplemental information

Document S1. Figures S1–S5 and Tables S1–S3

Data S1. RNA-seq dataset: organoids treated with SPARCL1 vs. 0.1% BSA (24 h)

Data S2. Mean Ct values for RT-qPCR experiments in this study

Data S3. Cell type quantification data from experiments in this study

Document S2. Article plus supplemental information

## References

[bib1] Alvira C.M. (2014). Nuclear factor-kappa-B signaling in lung development and disease: One pathway, numerous functions. Birth Defects Res. A Clin. Mol. Teratol..

[bib2] Armstrong L., Medford A.R.L., Uppington K.M., Robertson J., Witherden I.R., Tetley T.D., Millar A.B. (2004). Expression of Functional Toll-Like Receptor-2 and -4 on Alveolar Epithelial Cells. Am. J. Respir. Cell Mol. Biol..

[bib3] Beauchemin K.J., Wells J.M., Kho A.T., Philip V.M., Kamir D., Kohane I.S., Graber J.H., Bult C.J. (2016). Temporal dynamics of the developing lung transcriptome in three common inbred strains of laboratory mice reveals multiple stages of postnatal alveolar development. PeerJ.

[bib4] Brischetto C., Krieger K., Klotz C., Krahn I., Kunz S., Kolesnichenko M., Mucka P., Heuberger J., Scheidereit C., Schmidt-Ullrich R. (2021). NF-κB determines Paneth versus goblet cell fate decision in the small intestine. Development.

[bib5] Brownfield D.G., De Arce A.D., Ghelfi E., Gillich A., Desai T.J., Krasnow M.A. (2022). Alveolar cell fate selection and lifelong maintenance of AT2 cells by FGF signaling. Nat. Commun..

[bib6] Calvi C., Podowski M., Lopez-Mercado A., Metzger S., Misono K., Malinina A., Dikeman D., Poonyagariyon H., Ynalvez L., Derakhshandeh R. (2013). Hepatocyte Growth Factor, a Determinant of Airspace Homeostasis in the Murine Lung. PLoS Genet..

[bib7] Chiu M.C., Li C., Liu X., Yu Y., Huang J., Wan Z., Xiao D., Chu H., Cai J.-P., Zhou B. (2022). A bipotential organoid model of respiratory epithelium recapitulates high infectivity of SARS-CoV-2 Omicron variant. Cell Discov..

[bib8] Daniel E., Azizoglu D.B., Ryan A.R., Walji T.A., Chaney C.P., Sutton G.I., Carroll T.J., Marciano D.K., Cleaver O. (2018). Spatiotemporal heterogeneity and patterning of developing renal blood vessels. Angiogenesis.

[bib9] Ding B.S., Nolan D.J., Guo P., Babazadeh A.O., Cao Z., Rosenwaks Z., Crystal R.G., Simons M., Sato T.N., Worgall S. (2011). Endothelial-derived angiocrine signals induce and sustain regenerative lung alveolarization. Cell.

[bib10] Frank D.B., Penkala I.J., Zepp J.A., Sivakumar A., Linares-Saldana R., Zacharias W.J., Stolz K.G., Pankin J., Lu M., Wang Q. (2019). Early lineage specification defines alveolar epithelial ontogeny in the murine lung. Proc. Natl. Acad. Sci. USA.

[bib11] Gan K.J., Südhof T.C. (2020). SPARCL1 Promotes Excitatory But Not Inhibitory Synapse Formation and Function Independent of Neurexins and Neuroligins. J. Neurosci..

[bib12] Gillich A., Zhang F., Farmer C.G., Travaglini K.J., Tan S.Y., Gu M., Zhou B., Feinstein J.A., Krasnow M.A., Metzger R.J. (2020). Capillary cell-type specialization in the alveolus. Nature.

[bib13] Girard J.-P., Springer T.A. (1996). Modulation of Endothelial Cell Adhesion by Hevin, an Acidic Protein Associated with High Endothelial Venules (∗). J. Biol. Chem..

[bib14] Gkatzis K., Taghizadeh S., Huh D., Stainier D.Y.R., Bellusci S. (2018). Use of three-dimensional organoids and lung-on-a-chip methods to study lung development, regeneration and disease. Eur. Respir. J..

[bib15] Gkatzis K., Panza P., Peruzzo S., Stainier D.Y. (2021). Differentiation of mouse fetal lung alveolar progenitors in serum-free organotypic cultures. Elife.

[bib16] Gomez-Salinero J.M., Itkin T., Rafii S. (2021). Developmental angiocrine diversification of endothelial cells for organotypic regeneration. Dev. Cell.

[bib17] Gongidi V., Ring C., Moody M., Brekken R., Sage E.H., Rakic P., Anton E.S. (2004). SPARC-like 1 Regulates the Terminal Phase of Radial Glia-Guided Migration in the Cerebral Cortex. Neuron.

[bib18] Hassan D., Chen J. (2024). CEBPA restricts alveolar type 2 cell plasticity during development and injury-repair. Nat. Commun..

[bib19] Hogan B.L.M. (2018). Integrating Mechanical Force into Lung Development. Dev. Cell.

[bib20] Hu Y., Baud V., Oga T., Kim K.I., Yoshida K., Karin M. (2001). IKKα controls formation of the epidermis independently of NF-κB. Nature.

[bib21] Ii M., Matsunaga N., Hazeki K., Nakamura K., Takashima K., Seya T., Hazeki O., Kitazaki T., Iizawa Y. (2006). A Novel Cyclohexene Derivative, Ethyl (6R)-6-[N-(2-Chloro-4-fluorophenyl)sulfamoyl]cyclohex-1-ene-1-carboxylate (TAK-242), Selectively Inhibits Toll-Like Receptor 4-Mediated Cytokine Production through Suppression of Intracellular Signaling. Mol. Pharmacol..

[bib59] Kato K., Diéguez-Hurtado R., Park D.Y., Hong S.P., Kato-Azuma S., Adams S., Stehling M., Trappmann B., Wrana J.L., Koh G.Y. (2018). Pulmonary pericytes regulate lung morphogenesis. Nature Communications.

[bib22] Kaufman C.K., Fuchs E. (2000). It’s Got You Covered: Nf-κb in the Epidermis. J. Cell Biol..

[bib23] Klingler A., Regensburger D., Tenkerian C., Britzen-Laurent N., Hartmann A., Stürzl M., Naschberger E. (2020). Species-organ- And cell-type-dependent expression of SPARCL1 in human and mouse tissues. PLoS One.

[bib24] Kramer B.W., Kallapur S., Newnham J., Jobe A.H. (2009). Prenatal inflammation and lung development. Semin. Fetal Neonatal Med..

[bib25] Kucukdereli H., Allen N.J., Lee A.T., Feng A., Ozlu M.I., Conatser L.M., Chakraborty C., Workman G., Weaver M., Sage E.H. (2011). Control of excitatory CNS synaptogenesis by astrocyte-secreted proteins Hevin and SPARC. Proc. Natl. Acad. Sci. USA.

[bib26] Lazarus A., Del-Moral P.M., Ilovich O., Mishani E., Warburton D., Keshet E. (2011). A perfusion-independent role of blood vessels in determining branching stereotypy of lung airways. Development.

[bib27] Lee J.H., Bhang D.H., Beede A., Huang T.L., Stripp B.R., Bloch K.D., Wagers A.J., Tseng Y.H., Ryeom S., Kim C.F. (2014). Lung stem cell differentiation in mice directed by endothelial cells via a BMP4-NFATc1-thrombospondin-1 axis. Cell.

[bib28] Li J., Wang Z., Chu Q., Jiang K., Li J., Tang N. (2018). The Strength of Mechanical Forces Determines the Differentiation of Alveolar Epithelial Cells. Dev. Cell.

[bib29] Liu B., Xiang L., Ji J., Liu W., Chen Y., Xia M., Liu Y., Liu W., Zhu P., Jin Y. (2021). Sparcl1 promotes nonalcoholic steatohepatitis progression in mice through upregulation of CCL2. J. Clin. Investig..

[bib30] Londhe V.A., Nguyen H.T., Jeng J.-M., Li X., Li C., Tiozzo C., Zhu N., Minoo P. (2008). NF-kB induces lung maturation during mouse lung morphogenesis. Dev. Dyn..

[bib31] Londhe V.A., Maisonet T.M., Lopez B., Jeng J.-M., Xiao J., Li C., Minoo P. (2011). Conditional deletion of epithelial IKKβ impairs alveolar formation through apoptosis and decreased VEGF expression during early mouse lung morphogenesis. Respir. Res..

[bib32] Naschberger E., Liebl A., Schellerer V.S., Schütz M., Britzen-Laurent N., Kölbel P., Schaal U., Haep L., Regensburger D., Wittmann T. (2016). Matricellular protein SPARCL1 regulates tumor microenvironment–dependent endothelial cell heterogeneity in colorectal carcinoma. J. Clin. Investig..

[bib33] Negretti N.M., Plosa E.J., Benjamin J.T., Schuler B.A., Habermann A.C., Jetter C.S., Gulleman P., Bunn C., Hackett A.N., Ransom M. (2021). A single-cell atlas of mouse lung development. Development.

[bib34] Oeckinghaus A., Ghosh S. (2009). The NF-κB Family of Transcription Factors and Its Regulation. Cold Spring Harb. Perspect. Biol..

[bib35] Pasparakis M. (2012). Role of NF-κB in epithelial biology: NF-κB in epithelial biology. Immunol. Rev..

[bib36] Prince L.S., Okoh V.O., Moninger T.O., Matalon S. (2004). Lipopolysaccharide increases alveolar type II cell number in fetal mouse lungs through Toll-like receptor 4 and NF-κB. Am. J. Physiol. Lung Cell. Mol. Physiol..

[bib37] Rafii S., Cao Z., Lis R., Siempos I.I., Chavez D., Shido K., Rabbany S.Y., Ding B.S. (2015). Platelet-derived SDF-1 primes the pulmonary capillary vascular niche to drive lung alveolar regeneration. Nat. Cell Biol..

[bib38] Ramasamy S.K., Kusumbe A.P., Adams R.H. (2015). Regulation of tissue morphogenesis by endothelial cell-derived signals. Trends Cell Biol..

[bib39] Regensburger D., Tenkerian C., Pürzer V., Schmid B., Wohlfahrt T., Stolzer I., López-Posadas R., Günther C., Waldner M.J., Becker C. (2021). Matricellular Protein SPARCL1 Regulates Blood Vessel Integrity and Antagonizes Inflammatory Bowel Disease. Inflamm. Bowel Dis..

[bib40] Tabula Muris Consortium, Overall coordination, Logistical coordination, Organ collection and processing, Library preparation and sequencing, Computational data analysis, Cell type annotation, Writing group, Supplemental text writing group, Principal investigators (2018). Single-cell transcriptomics of 20 mouse organs creates a Tabula Muris. Nature.

[bib41] Schmidt A.F., Kannan P.S., Bridges J., Presicce P., Jackson C.M., Miller L.A., Kallapur S.G., Chougnet C.A., Jobe A.H. (2020). Prenatal inflammation enhances antenatal corticosteroid–induced fetal lung maturation. JCI Insight.

[bib42] Schmidt-Ullrich R., Aebischer T., Hülsken J., Birchmeier W., Klemm U., Scheidereit C. (2001). Requirement of NF-κB/Rel for the development of hair follicles and other epidermal appendices. Development.

[bib43] Seitz C.S., Lin Q., Deng H., Khavari P.A. (1998). Alterations in NF-κB function in transgenic epithelial tissue demonstrate a growth inhibitory role for NF-κB. Proc. Natl. Acad. Sci. USA.

[bib44] Seitz C.S., Deng H., Hinata K., Lin Q., Khavari P.A. (2000). Nuclear Factor κB Subunits Induce Epithelial Cell Growth Arrest1. Cancer Res..

[bib45] Shin H.-M., Kim M.-H., Kim B.H., Jung S.-H., Kim Y.S., Park H.J., Hong J.T., Min K.R., Kim Y. (2004). Inhibitory action of novel aromatic diamine compound on lipopolysaccharide-induced nuclear translocation of NF-κB without affecting IκB degradation. FEBS Lett..

[bib46] Singh S.K., Stogsdill J.A., Pulimood N.S., Dingsdale H., Kim Y.H., Pilaz L.-J., Kim I.H., Manhaes A.C., Rodrigues W.S., Pamukcu A. (2016). Astrocytes Assemble Thalamocortical Synapses by Bridging NRX1α and NL1 via Hevin. Cell.

[bib47] Strunz M., Simon L.M., Ansari M., Kathiriya J.J., Angelidis I., Mayr C.H., Tsidiridis G., Lange M., Mattner L.F., Yee M. (2020). Alveolar regeneration through a Krt8+ transitional stem cell state that persists in human lung fibrosis. Nat. Commun..

[bib48] Subramanian A., Tamayo P., Mootha V.K., Mukherjee S., Ebert B.L., Gillette M.A., Paulovich A., Pomeroy S.L., Golub T.R., Lander E.S., Mesirov J.P. (2005). Gene set enrichment analysis: A knowledge-based approach for interpreting genome-wide expression profiles. Proc. Natl. Acad. Sci. USA.

[bib49] Sullivan M.M., Sage E.H. (2004). Hevin/SC1, a matricellular glycoprotein and potential tumor-suppressor of the SPARC/BM-40/Osteonectin family. Int. J. Biochem. Cell Biol..

[bib50] Tabula Sapiens Consortium, Jones R.C., Karkanias J., Krasnow M.A., Pisco A.O., Quake S.R., Salzman J., Yosef N., Bulthaup B., Brown P. (2022). The Tabula Sapiens: A multiple-organ, single-cell transcriptomic atlas of humans. Science.

[bib51] Treutlein B., Brownfield D.G., Wu A.R., Neff N.F., Mantalas G.L., Espinoza F.H., Desai T.J., Krasnow M.A., Quake S.R. (2014). Reconstructing lineage hierarchies of the distal lung epithelium using single-cell RNA-seq. Nature.

[bib52] Vogel S.N., Wax J.S., Perera P.Y., Padlan C., Potter M., Mock B.A. (1994). Construction of a BALB/c congenic mouse, C.C3H-Lpsd, that expresses the Lpsd allele: analysis of chromosome 4 markers surrounding the Lps gene. Infect. Immun..

[bib53] Weibel E.R. (1984).

[bib54] Whitsett J.A., Alenghat T. (2015). Respiratory epithelial cells orchestrate pulmonary innate immunity. Nat. Immunol..

[bib55] Whitsett J.A., Wert S.E., Weaver T.E. (2015). Diseases of pulmonary surfactant homeostasis. Annu. Rev. Pathol..

[bib56] Yamamoto Y., Shiraishi I., Dai P., Hamaoka K., Takamatsu T. (2007). Regulation of embryonic lung vascular development by vascular endothelial growth factor receptors, Flk-1 and Flt-1. Anat. Rec..

[bib57] Zanini F., Che X., Knutsen C., Liu M., Suresh N.E., Domingo-Gonzalez R., Dou S.H., Zhang D., Pryhuber G.S., Jones R.C. (2023). Developmental diversity and unique sensitivity to injury of lung endothelial subtypes during postnatal growth. iScience.

[bib58] Zhao G., Gentile M.E., Xue L., Cosgriff C.V., Weiner A.I., Adams-Tzivelekidis S., Wong J., Li X., Kass-Gergi S., Holcomb N.P. (2024). Vascular endothelial-derived SPARCL1 exacerbates viral pneumonia through pro-inflammatory macrophage activation. Nat. Commun..

