# Supplementary material for: The lung microvasculature promotes alveolar type 2 cell differentiation via secreted SPARCL1
Source: Stem Cell Reports. 2025 Mar 20;20(4):102451. doi: 10.1016/j.stemcr.2025.102451 (PMC12069885; doi:10.1016/j.stemcr.2025.102451)
Supplement: Document S1. Figures S1–S5 and Tables S1–S3 [file mmc1.pdf]

Stem Cell Reports, Volume *20*

Supplemental Information

The lung microvasculature promotes alveolar type 2 cell differentiation via secreted SPARCL1

Paolo Panza, Hyun-Taek Kim, Till Lautenschläger, Janett Piesker, Stefan Günther, Yousef Alayoubi, Ondine Cleaver, Mario Looso, and Didier Y.R. Stainier

SUPPLEMENTAL FIGURES

Figure S1

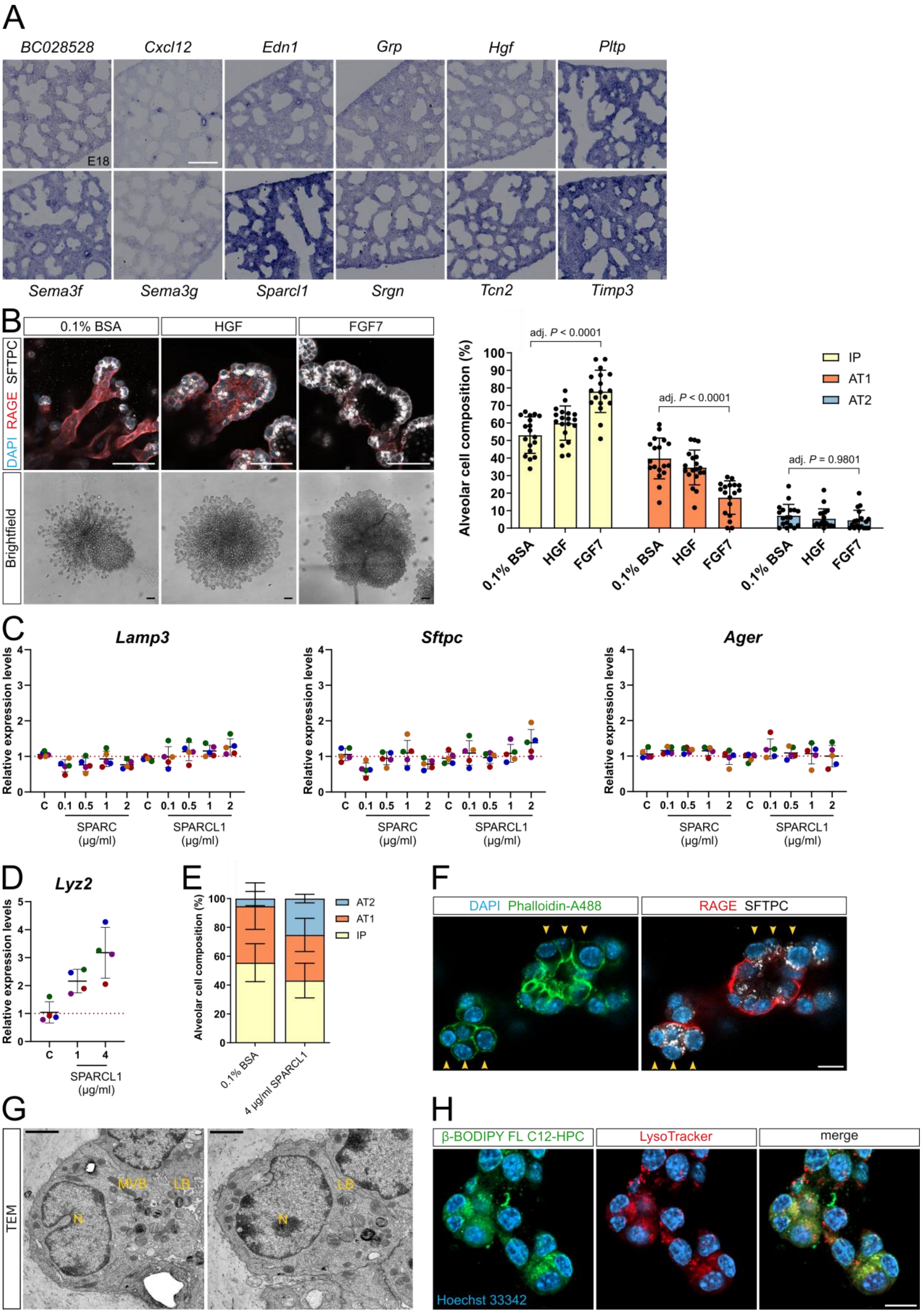

Figure S2

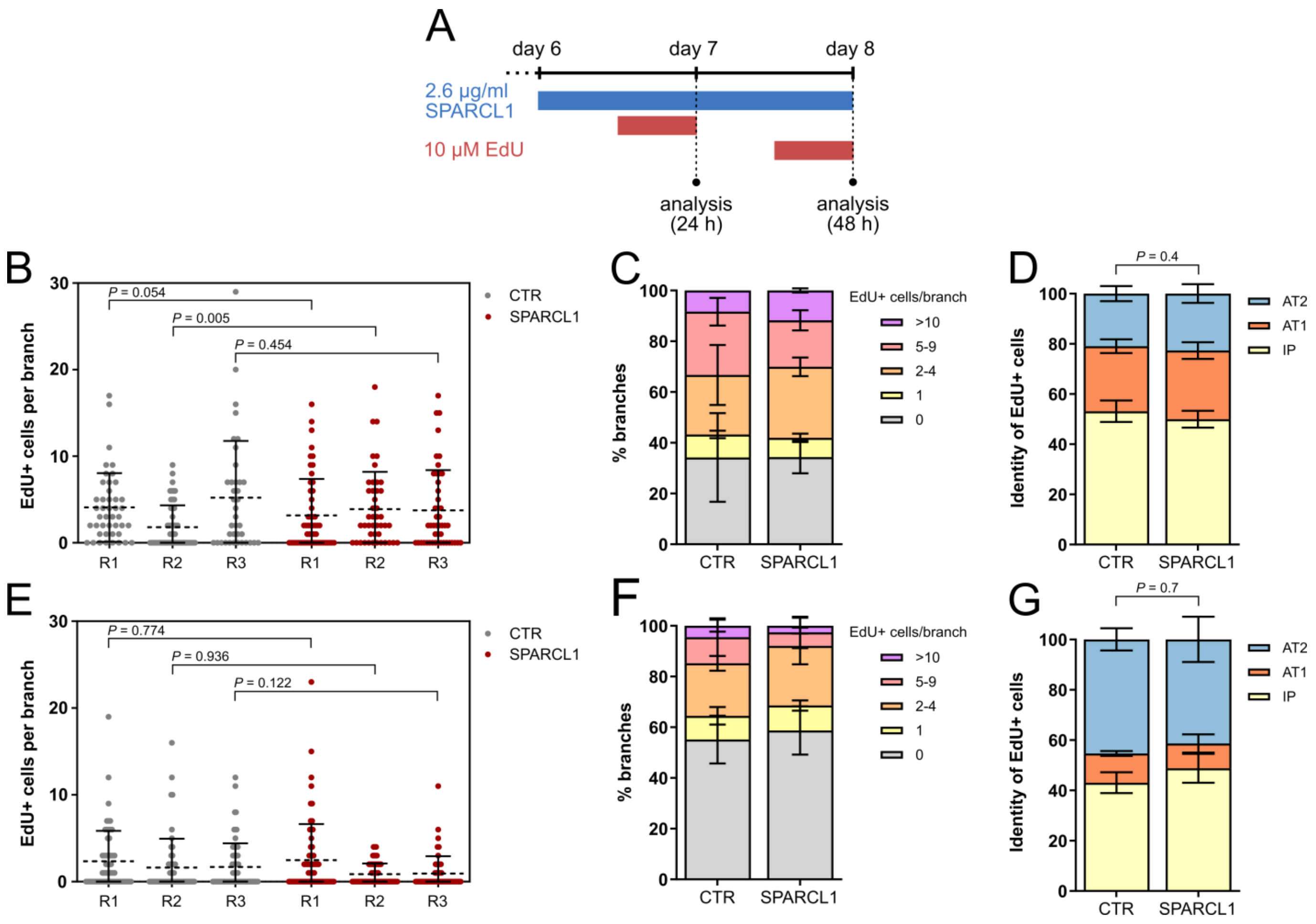

A
day 6
day 7
day 8
2.6 µg/ml SPARCL1
10 µM EdU
analysis (24 h)
analysis (48 h)
B
EdU+ cells per branch
P = 0.054
P = 0.005
P = 0.454
CTR
SPARCL1
R1
R2
R3
R1
R2
R3
C
% branches
EdU+ cells/branch
>10
5-9
2-4
1
0
CTR
SPARCL1
D
Identity of EdU+ cells
P = 0.4
AT2
AT1
IP
CTR
SPARCL1
E
EdU+ cells per branch
P = 0.774
P = 0.936
P = 0.122
CTR
SPARCL1
R1
R2
R3
R1
R2
R3
F
% branches
EdU+ cells/branch
>10
5-9
2-4
1
0
CTR
SPARCL1
G
Identity of EdU+ cells
P = 0.7
AT2
AT1
IP
CTR
SPARCL1

Figure S3

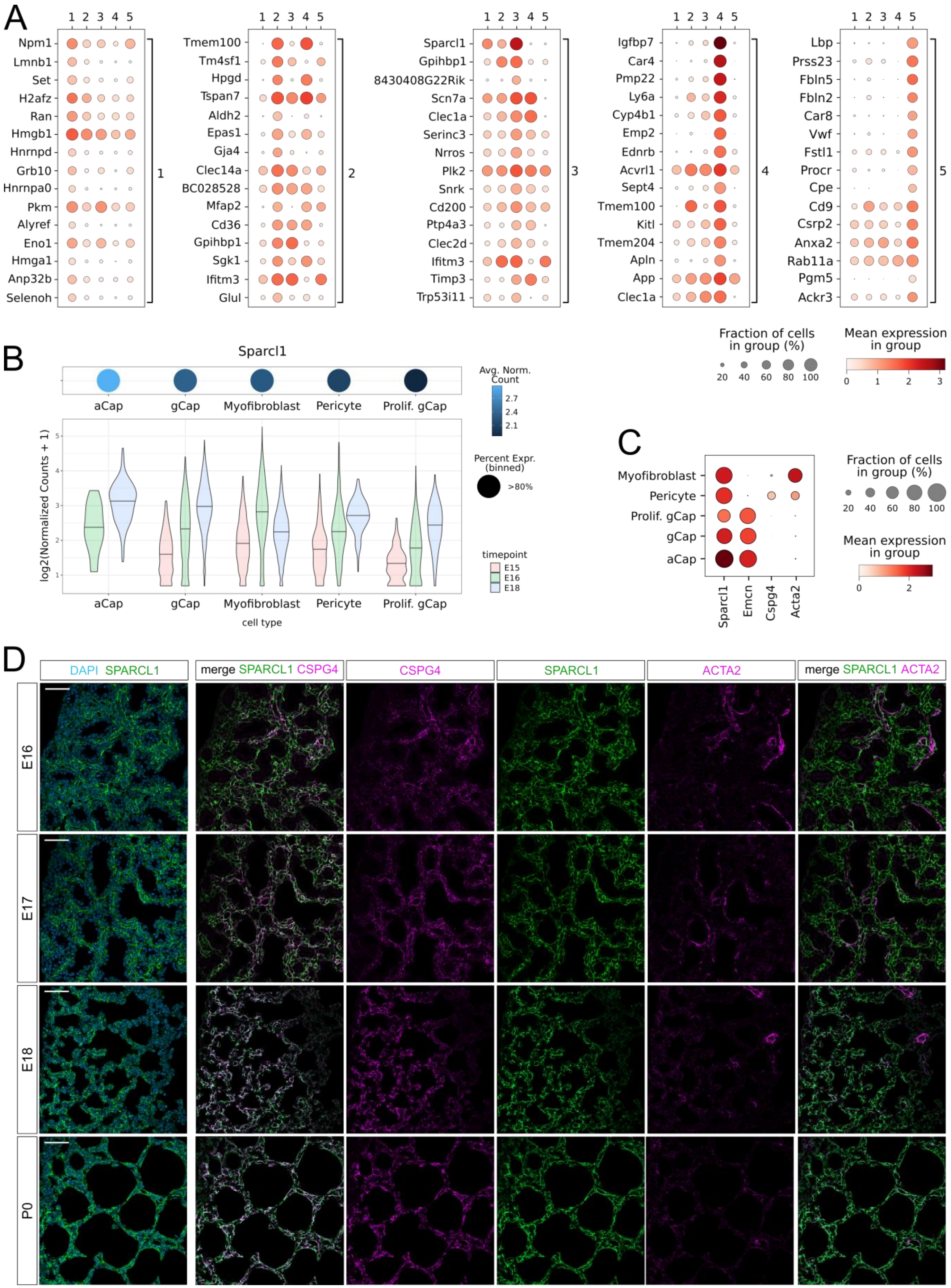
A
1 2 3 4 5
Npm1
Lmnb1
Set
H2afz
Ran
Hmgb1
Hnrnpd
Grb10
Hnrnpa0
Pkm
Alyref
Eno1
Hmga1
Anp32b
Selenoh
1
Tmem100
Tm4sf1
Hpgd
Tspan7
Aldh2
Epas1
Gja4
Clec14a
BC028528
Mfap2
Cd36
Gpihbp1
Sgk1
Ifitm3
Glul
2
Sparcl1
Gpihbp1
8430408G22Rik
Scn7a
Clec1a
Serinc3
Nrros
Plk2
Snrk
Cd200
Ptp4a3
Clec2d
Ifitm3
Timp3
Trp53i11
3
Igfbp7
Car4
Pmp22
Ly6a
Cyp4b1
Emp2
Ednrb
Acvrl1
Sept4
Tmem100
Kitl
Tmem204
Apln
App
Clec1a
4
Lbp
Prss23
Fbln5
Fbln2
Car8
Vwf
Fstl1
Procr
Cpe
Cd9
Csrp2
Anxa2
Rab11a
Pgm5
Ackr3
5
Fraction of cells in group (%)
20 40 60 80 100
Mean expression in group
0 1 2 3
B
Sparcl1
aCap gCap Myofibroblast Pericyte Prolif. gCap
Avg. Norm. Count
2.7
2.4
2.1
log2(Normalized Counts + 1)
Percent Expr. (binned)
>80%
timepoint
E15
E16
E18
cell type
C
Myofibroblast
Pericyte
Prolif. gCap
gCap
aCap
Sparcl1
Emcn
Cspg4
Acta2
Fraction of cells in group (%)
20 40 60 80 100
Mean expression in group
0 2
D
DAPI SPARCL1
merge SPARCL1 CSPG4
CSPG4
SPARCL1
ACTA2
merge SPARCL1 ACTA2
E16
E17
E18
P0

Figure S4

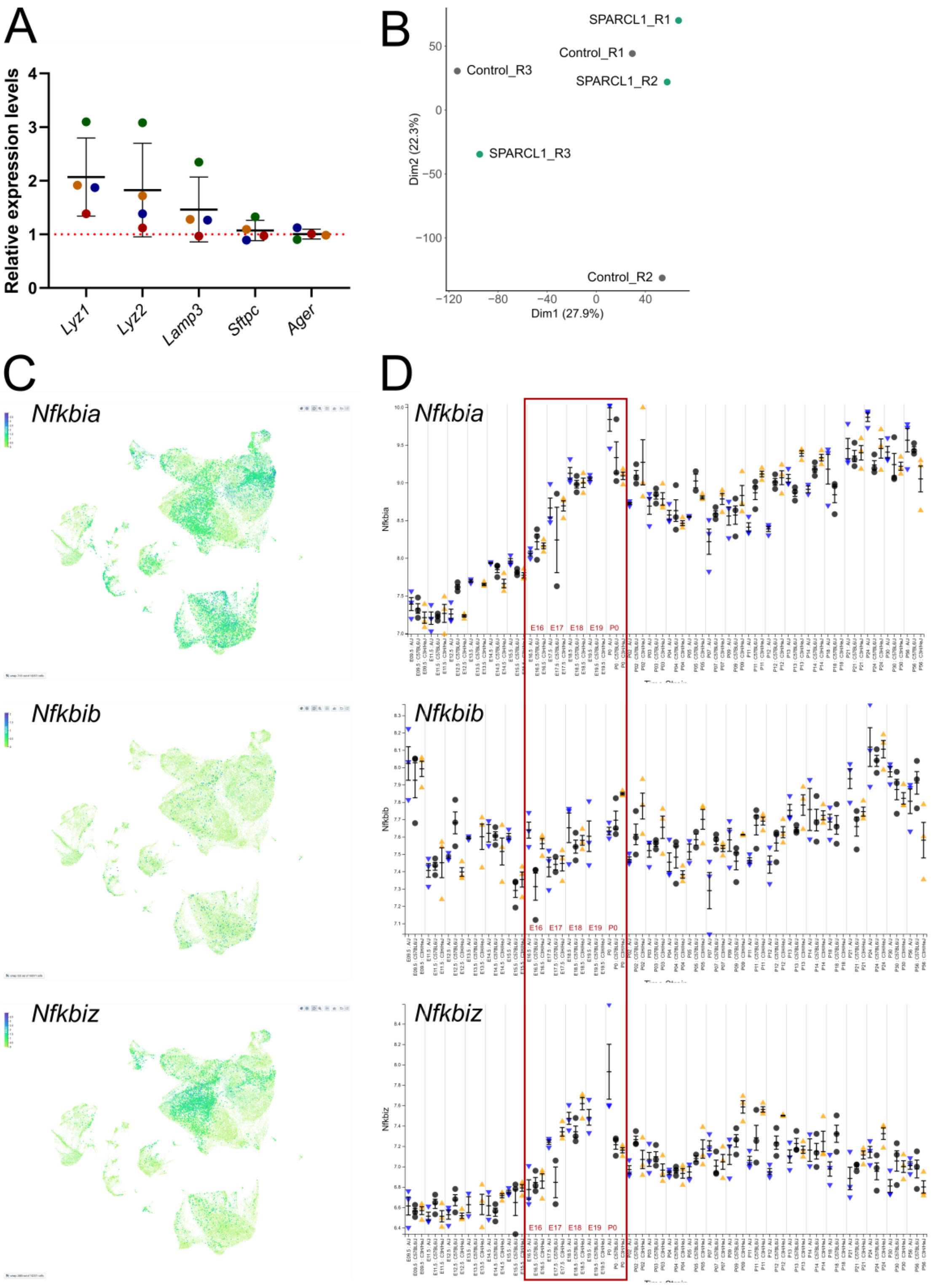
A
Relative expression levels
Lyz1
Lyz2
Lamp3
Sftpc
Ager
B
SPARCL1_R1
Control_R1
Control_R3
SPARCL1_R2
SPARCL1_R3
Control_R2
Dim2 (22.3%)
Dim1 (27.9%)
C
Nfkbia
Nfkbib
Nfkbiz
D
Nfkbia
Nfkbib
Nfkbiz
E16
E17
E18
E19
P0

Figure S5

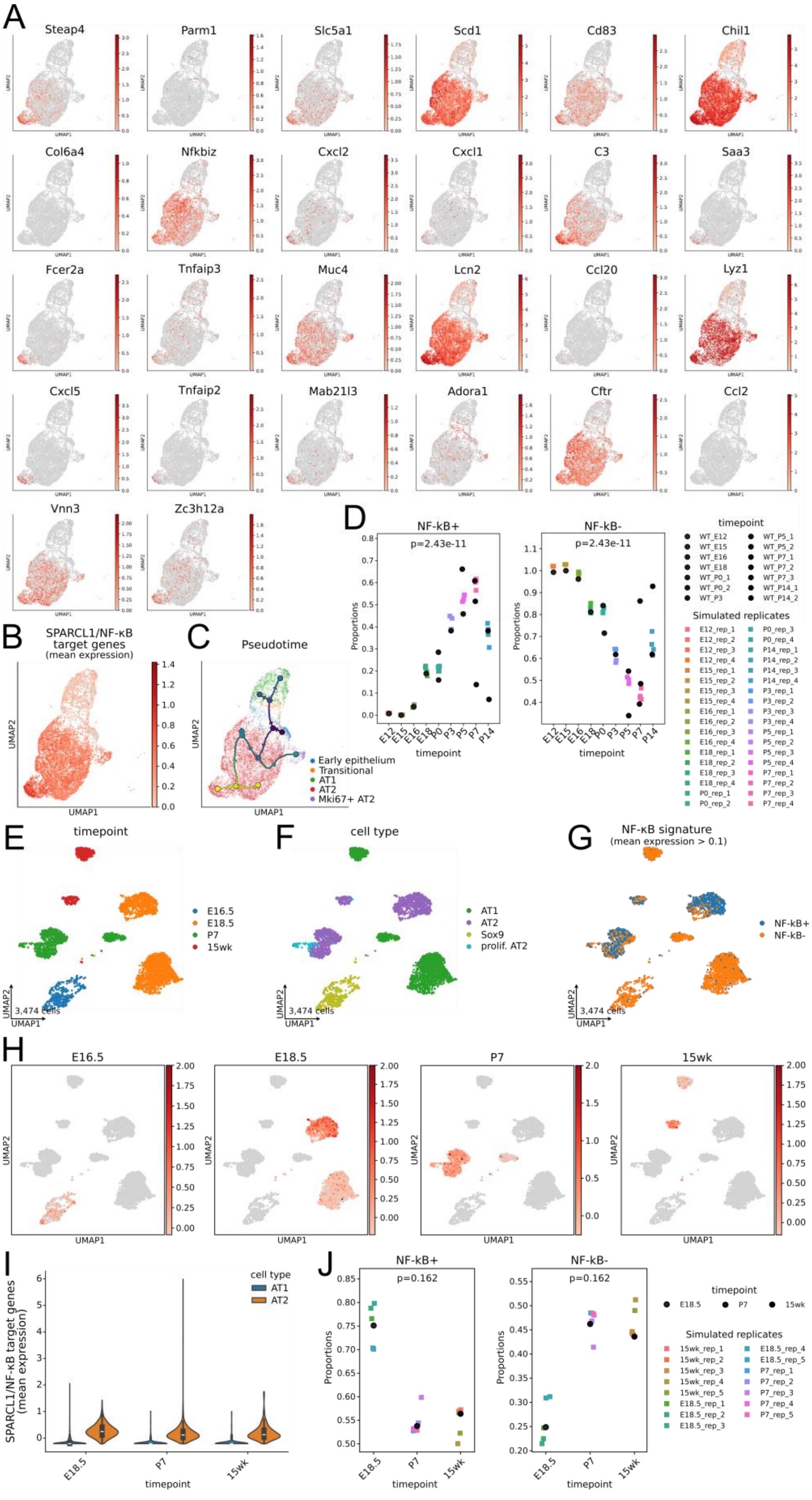

A
Steap4
Parm1
Slc5a1
Scd1
Cd83
Chil1
Col6a4
Nfkbiz
Cxcl2
Cxcl1
C3
Saa3
Fcer2a
Tnfaip3
Muc4
Lcn2
Ccl20
Lyz1
Cxcl5
Tnfaip2
Mab21l3
Adora1
Cftr
Ccl2
Vnn3
Zc3h12a
B
SPARCL1/NF-κB target genes (mean expression)
C
Pseudotime
Early epithelium
Transitional
AT1
AT2
Mki67+ AT2
D
NF-kB+
p=2.43e-11
NF-kB-
p=2.43e-11
Proportions
timepoint
Simulated replicates
E
timepoint
E16.5
E18.5
P7
15wk
3,474 cells
F
cell type
AT1
AT2
Sox9
prolif. AT2
G
NF-κB signature (mean expression > 0.1)
NF-kB+
NF-kB-
H
E16.5
E18.5
P7
15wk
UMAP1
UMAP2
I
SPARCL1/NF-κB target genes (mean expression)
cell type
AT1
AT2
J
NF-kB+
p=0.162
NF-kB-
p=0.162
Proportions
timepoint
Simulated replicates

SUPPLEMENTAL FIGURE LEGENDS

Figure S1. (A) *In situ* hybridization for genes encoding candidate EC secreted proteins on E18 lung cryosections. *Cxcl12* and *Edn1* are expressed in proximal blood vessels; *Sparcl1* and *Pltp* are expressed in a reticular pattern within the distal lung interstitium, consistent with their expression in blood capillary and/or mural cells. *Timp3* is highly expressed in AT1 cells. Scale bar: 100 µm. **(B)** Growth factor treated organoids display a morphological phenotype and an increased proportion of IP cells. Left, top row: whole-mount organoid immunostaining for SFTPC (IP and AT2 cells) and RAGE (IP and AT1 cells). Organoids treated with HGF (1 µg/mL) and FGF7 (10 ng/mL) contain more IP cells ($SFTPC^{+}/RAGE^{+}$) and fewer AT1 cells ($SFTPC^{-}/RAGE^{+}$). Left, bottom row: brightfield images of representative organoids treated with 0.1% BSA, HGF, and FGF7. Right: quantification of cell identities in growth factor-treated organoids. FGF7 treated organoids display an increased proportion of IP cells at the expense of AT1 cells (adj. $p < 0.0001$, $n = 3$ dams, at least 6 organoids per condition). Scale bars: 50 µm (top row); 100 µm (bottom row). Data are presented as mean ± SD. *p* values are from one-way ANOVA, Tukey's multiple comparison testing. **(C)** Minor dose dependent increase in *Lamp3* and *Sftpc* mRNA levels in organoids treated with SPARCL1, but not with SPARC. *Ager* mRNA levels did not change upon treatment with recombinant SPARCL1 or SPARC ($n = 5$ dams, at least 12 organoids per condition). Data are presented as mean ± SD. **(D)** High dose SPARCL1 (4 µg/mL) further increases *Lyz2* mRNA levels in organoids, compared with the lower dose (1 µg/mL) used for functional screening ($n = 4$ dams, at least 12 organoids per condition). Data are presented as mean ± SD. **(E)** Increased proportion of AT2 cells in organoids treated with SPARCL1 at 4 µg/mL ($n = 3$ dams, at least 4 organoids per condition). AT2: alveolar type 2 cell ($SFTPC^{+}/RAGE^{-}$); AT1: alveolar type 1 cell ($SFTPC^{-}/RAGE^{+}$); IP: intermediate progenitor cell ($SFTPC^{+}/RAGE^{+}$). Data are presented as mean ± SD. **(F)** High magnification detail of AT2 cells in SPARCL1-treated organoids. AT2 cells contain SFTPC-positive organelles (white) and are devoid of RAGE signal (red) on their basal membrane. Phalloidin staining of cortical actin (green) marks the cell boundaries. Scale bar: 10 µm. **(G)** Transmission electron microscopy (TEM) images of representative epithelial cells in distal regions of organoid outgrowths. Panels show examples of AT2 cells, as identified by the presence of characteristic lamellar bodies (LBs) and multivesicular bodies (MVBs). N: nucleus. Scale bars: 2 µm. **(H)** Distal tip cells in organoids take up labeled phosphatidylcholine into acidic organelles; live fluorescence in whole-mount organoids. Hoechst 33342 (DNA, blue); β-BODIPY FL C12-HPC (fluorescent phosphatidylcholine, green); LysoTracker Deep Red (lamellar bodies/lysosomes, red). Distal tip cells consistent with AT2 cell morphology and characteristics display co-localizing green and red signals. Scale bar: 10 µm.

Figure S2. (A) Workflow schematic for the analysis of cell proliferation in organoids. Control and SPARCL1-treated (2.6 µg/mL) organoids ($n = 3$ dams, at least 12 organoids per condition) were terminally labeled by 10 µM EdU for 12 h and samples were collected after 24 and 48 h. **(B)** Distribution of EdU^{+} cell counts per branch in control (grey) and SPARCL1-treated (red) organoids (day 7). One replicate (R2) out of three displayed a significant increase in EdU^{+} cell number in SPARCL1-treated organoids ($p = 0.005$). **(C)** Relative distribution of EdU^{+} cell counts per organoid branch (day 7). **(D)** Proportion (percentage) of EdU^{+} cell identities observed on day 7. The proportion of EdU^{+} AT2 cells did not change significantly in SPARCL1-treated organoids. **(E)** Distribution of EdU^{+} cell counts per branch in control (grey) and SPARCL1-treated (red) organoids (day 8). The EdU^{+} cell number did not change significantly in SPARCL1-treated organoids. **(F)** Relative distribution of EdU^{+} cell counts per organoid branch (day 8). **(G)** Proportion (percentage) of

EdU⁺ cell identities observed on day 8. The proportion of EdU⁺ AT2 cells did not change significantly in SPARCL1-treated organoids. All data are presented as mean ± SD. All *p* values are from Mann-Whitney U test.

Figure S3. (A) Marker gene expression analysis of EC clusters identified (E12-P3). Cells in clusters 2 and 3 are identified as general capillary (gCap) cells because of the exclusive expression of *Gpihbp1* (Gillich et al., 2020). *Tmem100* expression identifies clusters 2 and 4 as predominantly postnatal (Liu et al., 2022). *Car4*, *Kitl* and *Apln* expression marks cluster 4 cells, identifying them as alveolar capillary (aCap) cells (Gillich et al., 2020). **(B)** The mRNA expression levels of *Sparcl1* are comparable between lung ECs and pericytes at stages E15 to E18. Normalized *Sparcl1* read counts from single lung cells profiled at E15, E16, and E18. Data and cell type annotation from Negretti et al., 2021. **(C)** Expression of *Emcn*, *Cspg4*, and *Acta2* distinguish ECs, pericytes, and myofibroblasts in the E15-E18 lung. Data and cell type annotation from Negretti et al., 2021. **(D)** SPARCL1 expression prevails in distal lung pericytes compared with myofibroblasts between E16-P0. Immunostaining of E16-P0 (top to bottom rows) lung cryosections, single optical sections. Left: overview of SPARCL1 expression (green) in distal lung regions. Other panels (left to right): merge between SPARCL1 (green) and CSPG4 (magenta, pericytes); single channel CSPG expression (magenta); single channel SPARCL1 expression (green); single channel ACTA2 expression (magenta, myofibroblasts); merge between SPARCL1 (green) and ACTA2 (magenta, myofibroblasts). Scale bars: 50 µm.

Figure S4. (A) The recombinant SPARCL1 protein used for the RNA-seq experiments is biologically active. Recombinant SPARCL1 induced *Lyz1*, *Lyz2* and *Lamp3* upregulation (48 h treatments) in organoids from the same primary tissue source as those profiled by RNA-seq (24 h treatments). $n = 4$ dams, at least 16 organoids per condition. Replicates represented in green, orange and blue were selected for RNA-seq. Data are presented as mean ± SD. **(B)** Principal component analysis (PCA) plot for sequenced RNA samples shows intermixing between conditions (control and SPARCL1-treated). **(C)** Broad expression and perinatal increase in whole-lung expression levels for *Nfkbia* and *Nfkbiz*. Lower and stable expression level for *Nfkbib* in the developing lung. Gene expression levels (green-to-blue color scale) in a single cell atlas comprising mouse lung development (E12-P14, Negretti et al., 2021). **(D)** Gene expression level dynamics in whole lungs, data for 3 inbred mouse strains (Beauchemin et al., 2016). Blue: A/J; black: C57BL6/J, yellow: C3H/HeJ.

Figure S5. (A) Split-view of expression levels in single epithelial cells for genes identified as SPARCL1-responsive. Plots include single alveolar epithelial cell transcriptomes from E12 to P7. Data from Negretti et al., 2021. **(B)** Mean expression level for all identified SPARCL1/NF-κB target genes in alveolar epithelial cells profiled from E12 to P7. A subset of epithelial cells express SPARCL1-responsive genes starting at E18 and through postnatal stages P0-P7. **(C)** Similarity-based pseudotime analysis correlates increasing SPARCL1/NF-κB target gene expression with maturing AT2 cells. Lineage progression: purple to yellow. **(D)** The proportion of epithelial cells expressing SPARCL1/NF-κB target genes (NF-kB+) increases significantly from E18 in developing lungs. Conversely, epithelial cells characterized as NF-κB negative (NF-kB-) decrease in prevalence from E18. *p* values empirical Bayes moderated ANOVA test.
(E) Leiden clustering of single lung alveolar epithelial cell transcriptomes from 4 stages (E16.5, E18.5, P7, 15 weeks). Data from Hassan and Chen, 2024. **(F-G)** Cells categorized to express the SPARCL1/NF-κB target genes (blue, NF-kB+) are found in higher proportion within clusters annotated as AT2 cells (purple) at all analyzed stages. **(H)** Expression levels in single epithelial cells for genes identified as SPARCL1-responsive. Plots display single alveolar

epithelial cell transcriptomes from each stage analyzed. **(I)** Stage-resolved comparison of the mean expression level for SPARCL1/NF-κB target genes between AT1 and AT2 cells, showing consistently higher expression in AT2 cells independent from the time point, at comparable levels across the stages analyzed. **(J)** In mouse lungs, the proportion of AT2 cells expressing SPARCL1-responsive NF-κB genes (NF-kB+) decreases from E18.5 to 15 weeks, albeit not significantly. *p* values are from empirical Bayes moderated ANOVA test.

.

Table S1

Ensembl gene ID	Ensembl gene	Condition mean E18.5 lung	E18.5 lung vs. E15.5 lung log2 fold change	Signal peptide (found or predicted)	TM Keyword found (UniProt)	GPI anchor	Matrisome cell source	Matrisome type	Matrisome level	Matrisome secreted	Kidney Pancreas Log2FC E18.5 > E15.5	Exp. Level E18.5 > E15.5 (15)	scRNA-seq expression (LGEA, E18)	scRNA-seq marker gene (LGEA, E18)
ENSMUSG00000029375	Cxcl15	6649	8.55	TRUE	FALSE		AT2	Secreted Factors	Matrisome-associated	TRUE	FALSE	Y	AT2	Y
ENSMUSG00000026874	Hc	578	6.82	TRUE	FALSE					FALSE	FALSE	Y	AT2	Y
ENSMUSG00000031722	Hp	1133	6.81	TRUE	FALSE					FALSE	FALSE	Y	Myeloid; Epi	
ENSMUSG00000063011	Msln	1298	6.76	TRUE	FALSE	Y				FALSE	FALSE	Y	AT1	Y
ENSMUSG00000026822	Lcn2	268	6.04	TRUE	FALSE					FALSE	FALSE	Y	AT2	Y
ENSMUSG00000069516	Lyz2	5166	5.91	TRUE	FALSE					FALSE	TRUE	Y	Myeloid; AT2	
ENSMUSG00000000805	Car4	655	5.38	TRUE	FALSE	Y				FALSE	FALSE	Y	EC; Myeloid	
ENSMUSG00000002204	Napsa	1264	5.36	TRUE	FALSE					FALSE	FALSE	Y	Epithelium	Y
ENSMUSG00000033880	Lgals3bp	673	4.78	TRUE	FALSE					FALSE	TRUE	Y	Myeloid, EC	
ENSMUSG00000060962	Dmkn	61	3.71	TRUE	FALSE					FALSE	FALSE	Y low	Epithelium	
ENSMUSG00000030093	Wnt7a	27	3.32	TRUE	FALSE		Other	Secreted Factors	Matrisome-associated	TRUE	FALSE		AT1	Y
ENSMUSG00000007279	Scube2	56	2.54	TRUE	FALSE		EC	Secreted Factors	Matrisome-associated	TRUE	TRUE	Y	Matrix FB	Y
ENSMUSG00000028864	Hgf	96	2.46	TRUE	FALSE		EC	Secreted Factors	Matrisome-associated	TRUE	FALSE	Y low	Myeloid; EC	
ENSMUSG00000030790	Adm	136	2.43	TRUE	FALSE					FALSE	FALSE	Y	EC; FB	
ENSMUSG00000021367	Edn1	2251	2.32	TRUE	FALSE		EC			FALSE	FALSE	Y	EC	Y
ENSMUSG00000020044	Timp3	16922	2.27	TRUE	FALSE			ECM Regulators	Matrisome-associated	FALSE	FALSE	Y	AT1; EC; FB	
ENSMUSG00000017754	Pltp	3914	2.17	TRUE	FALSE					FALSE	FALSE	Y low	EC; FB	
ENSMUSG00000020432	Tcn2	7172	2.17	TRUE	FALSE					FALSE	FALSE	Y	Ubi	
ENSMUSG00000038264	Sema7a	1750	2.02	TRUE	FALSE	Y		ECM-affiliated Proteins	Matrisome-associated	FALSE	TRUE	Y	EC	Y
ENSMUSG00000039899	Fgl2	313	2.02	TRUE	FALSE			ECM Glycoproteins	Core matrisome	FALSE	FALSE	Y	EC; Myeloid	
ENSMUSG00000034684	Sema3f	3472	1.98	TRUE	FALSE			ECM-affiliated Proteins	Matrisome-associated	FALSE	FALSE	Y	EC	
ENSMUSG00000029309	Sparcl1	54062	1.73	TRUE	FALSE			ECM Glycoproteins	Core matrisome	FALSE	FALSE	Y	EC; PC; FB	
ENSMUSG00000024517	Grp	273	1.50	TRUE	FALSE					FALSE	FALSE	Y low	EC; PC	
ENSMUSG00000026365	Cfh	96	1.46	TRUE	FALSE					FALSE	FALSE	Y	Myeloid, FB	
ENSMUSG00000060802	B2m	7019	1.40	TRUE	FALSE					FALSE	FALSE	Y	Ubi	
ENSMUSG00000021242	Npc2	5784	1.35	TRUE	FALSE					FALSE	FALSE	Y	AT2	Y
ENSMUSG00000021186	Fbln5	3173	1.29	TRUE	FALSE			ECM Glycoproteins	Core matrisome	FALSE	FALSE	Y	AT1; FB; EC	
ENSMUSG00000038543	BC028528	1381	1.28	TRUE	FALSE					FALSE	FALSE	Y low	EC; PC	
ENSMUSG00000046618	Olfml2a	378	1.27	TRUE	FALSE					FALSE	FALSE		EC; PC	
ENSMUSG00000020077	Srgn	3599	1.24	TRUE	FALSE			Proteoglycans	Core matrisome	FALSE	FALSE	Y	EC; Myeloid	
ENSMUSG00000020902	Ntn1	320	1.17	TRUE	FALSE			ECM Glycoproteins	Core matrisome	FALSE	FALSE			
ENSMUSG00000061353	Cxcl12	3047	1.09	TRUE	FALSE		EC	Secreted Factors	Matrisome-associated	TRUE	FALSE		EC; PC	
ENSMUSG00000021904	Sema3g	609	1.08	TRUE	FALSE			ECM-affiliated Proteins	Matrisome-associated	FALSE	FALSE	Y	EC	
ENSMUSG00000031503	Col4a2	16698	1.05	TRUE	FALSE			Collagens	Core matrisome	FALSE	FALSE	Y	EC; PC; FB	
ENSMUSG00000064080	Fbln2	752	1.03	TRUE	FALSE			ECM Glycoproteins	Core matrisome	FALSE	FALSE		EC; PC; FB	

Table S2

Ensembl gene id	Ensembl gene	baseMean	baseMeanA CONTROL	baseMeanB TREATED	log2FoldChange TREATED/CONTROL	pvalue	padj
ENSMUSG00000040026	Saa3	97	4	190	5.16	0.00	0.00
ENSMUSG00000029371	Cxcl5	116	15	217	3.74	0.00	0.00
ENSMUSG00000026822	Lcn2	211	48	374	2.94	0.00	0.00
ENSMUSG00000024164	C3	329	110	548	2.31	0.00	0.00
ENSMUSG00000026166	Ccl20	30	11	48	2.05	0.00	0.00
ENSMUSG00000044313	Mab21l3	82	33	132	1.96	0.00	0.00
ENSMUSG00000058427	Cxcl2	38	17	60	1.77	0.00	0.00
ENSMUSG00000005540	Fcer2a	81	39	124	1.65	0.00	0.00
ENSMUSG00000029380	Cxcl1	78	38	117	1.60	0.00	0.00
ENSMUSG00000012428	Steap4	301	156	446	1.51	0.00	0.04
ENSMUSG00000035385	Ccl2	81	42	119	1.49	0.00	0.00
ENSMUSG00000064246	Chil1	683	359	1007	1.48	0.00	0.00
ENSMUSG00000021281	Tnfaip2	66	38	94	1.29	0.00	0.01
ENSMUSG00000015396	Cd83	164	101	228	1.17	0.00	0.00
ENSMUSG00000020010	Vnn3	273	171	374	1.12	0.00	0.00
ENSMUSG00000042429	Adora1	375	299	451	0.59	0.00	0.00
ENSMUSG00000019850	Tnfaip3	594	490	697	0.51	0.00	0.00
ENSMUSG00000041301	Cftr	653	546	760	0.48	0.00	0.02
ENSMUSG00000037071	Scd1	1332	1128	1535	0.44	0.00	0.00
ENSMUSG00000011034	Slc5a1	53	33	74	1.16	0.00	0.06
ENSMUSG00000042677	Zc3h12a	197	156	238	0.60	0.00	0.07
ENSMUSG00000069515	Lyz1	101	70	132	0.91	0.00	0.07
ENSMUSG00000032572	Col6a4	338	262	415	0.66	0.00	0.10
ENSMUSG00000034981	Parm1	230	187	273	0.54	0.00	0.10
ENSMUSG00000118672	Muc4	414	345	482	0.48	0.00	0.10
ENSMUSG00000027533	Fabp5	182	141	223	0.65	0.00	0.13
ENSMUSG00000048732	Klhl11	135	100	170	0.76	0.00	0.18

Table S3

Gene	Forward Sequence (5’-3’)	Reverse Sequence (5’-3’)
Actb	CTCTGGCTCCTAGCACCATGAAGA	GTAAAACGCAGCTCAGTAACAGT
Maea	CACTGAACAAACGCTTCCGAG	GGCAACTACTCAAGGTCTTCTC
Ager	ACGGGACTCTTTACACTGCG	CAACCAACAGCTGAATGCCC
Sftpc	AGCAGACACCATCGCTACCT	GCAGTAGGTTCCTGGAGCTG
Lamp3	TCCAAAAGCCAGAGGCTATCT	ACTGGGGTTACTGTTTTCATTGT
Lyz1	GAGACCGAAGCACCGACTATG	CGGTTTTGACATTGTGTTCGC
Lyz2 (pair 1)	GATGGCAAAACCCCAAGAGC	CAGACTCCGCAGTTCCGAAT
Lyz2 (pair 2)	ATGGAATGGCTGGCTACTATGG	ACCAGTATCGGCTATTGATCTGA
Nfkbia	TGAAGGACGAGGAGTACGAGC	TTCGTGGATGATTGCCAAGTG
Nfkbib	GCGGATGCCGATGAATGGT	TGACGTAGCCAAAGACTAAGGG
Nfkbiz	GCTCCGACTCCTCCGATTTC	GAGTTCTTCACGCGAACACC

Table S1. Candidate endothelial cell (EC) secreted factors. Genes were prioritized based on their higher expression at E18.5 compared with E15.5 in lung ECs (log2FC > 1). Only genes encoding secreted proteins (with predicted signal peptide, without transmembrane domain (TM)) were included in the analysis. Source transcriptomic data from (Daniel et al., 2018).

Table S2. SPARCL1-responsive gene set. Genes upregulated upon SPARCL1 treatment of mouse lung organoids (24 h) are shown (adj. $p < 0.2$; $p = 0$).

Table S3. RT-qPCR primer sequences used in the study.

Data S1 (separate file). RNA-seq dataset: organoids treated with SPARCL1 vs. 0.1% BSA (24 h).

Data S2 (separate file). Mean Ct values for RT-qPCR experiments in this study.

Data S3 (separate file). Cell type quantification data from experiments in this study.

SUPPLEMENTAL METHODS

cDNA preparation and RT-qPCR

To isolate total RNA, pooled organoids (10–20 organoids, from at least 6 wells per condition) were lysed in TRIzol Reagent (Invitrogen 15596018), followed by aqueous phase purification using the RNA Clean and Concentrator-5 kit (Zymo Research R1016) and DNAse digestion on column (Qiagen 79254). 500 ng total RNA were used for cDNA synthesis using the Maxima First Strand cDNA synthesis kit (Thermo Scientific K1672). qPCR was performed using the DyNAmo ColorFlash SYBR green qPCR kit (Thermo Scientific F416XL) on a CFX Connect Real-Time System (Bio-Rad). qPCR reactions were set up in technical duplicates and data from at least three biological replicates (different dams) were collected, except for experiments in Figure 3I where $n = 2$ dams. Ct values were normalized to the mouse *Actb* (beta actin) and *Maea* (Macrophage erythroblast attacher) genes (Shin et al., 2022). The qPCR primer sequences for *Actb*, *Maea*, *Ager* (Advanced glycosylation end product-specific receptor), *Sftpc* (Surfactant protein C), *Lamp3* (Lysosome associated membrane glycoprotein 3), *Lyz1*, *Lyz2* (Lysozyme 1 and 2), *Nfkbia*, *Nfkbib*, *Nfkbiz* (Nuclear factor of kappa light polypeptide gene enhancer in B cells inhibitor, alpha, beta, and zeta) are shown in Table S3. The Ct values are shown in Data S2.

Lung tissue immunostaining

Fetal mouse lungs were perfused with PBS and fixed overnight at 4°C in 4% PFA. After PBS washes, the lungs were brought in 10 and 30% sucrose in PBS for cryoprotection. After OCT embedding (Tissue-Tek 4583), 10 µm cryosections were cut from frozen tissue blocks using a Leica CM1950 cryotome.

Antibodies

Primary antibodies used are: anti-CDH1 (E-cadherin, 1:500, Santa Cruz sc-59778), anti-EMCN (Endomucin, 1:250, Santa Cruz sc-65495, R&D AF4666), anti-RAGE (Advanced glycation end product-specific receptor, 1:250, R&D MAB1179), anti-Pro-SFTPC (Pro-surfactant protein C, 1:500, Millipore AB3786), anti-CSPG4/NG2 (Chondroitin sulfate proteoglycan 4, 1:500, Millipore AB5320), anti-ACTA2-Cy3 (Alpha smooth muscle actin, 1:1000, Sigma C6198), anti-SPARCL1 (Secreted protein acidic and rich in cysteine-like protein 1, 1:250, R&D AF2836), anti-TLR4 (Toll-like receptor 4, 1:250, Proteintech 19811-1-AP), and anti-LAMP3 (Lysosome associated membrane glycoprotein 3, 1:250, Dendritics DDX0192).

Cell proliferation analysis

Terminal labeling of proliferating cells was performed by incubating organoids with 10 µM EdU for 12 h. After fixation, organoids were immunostained and EdU incorporation was revealed using the Click-iT EdU Cell Proliferation Kit (Thermo Scientific C10340). Between 38 and 67 branching structures per condition per replicate were blindly selected for imaging. A total of 465 (control) and 508 (SPARCL1) cells were identified on day 7. A total of 329 (control) and 247 (SPARCL1) cells were identified on day 8. Statistical significance was computed by Mann-Whitney U test.

Library preparation

For RNA-seq analysis, total RNA was isolated from control and SPARCL-treated organoids using the miRNeasy micro Kit (Qiagen) combined with on-column DNase digestion (RNase-Free DNase Set, Qiagen) to avoid contamination by genomic DNA. RNA and library preparation integrity were verified with LabChip Gx Touch 24 (Perkin Elmer). 2 µg of total RNA was used as input for VAHTS Stranded mRNAseq V6 Library preparation following manufacturer's protocol (Vazyme). Sequencing was performed on NextSeq2000 instrument (Illumina) with 1x72bp single end setup.

RNA-seq analysis

Trimmomatic version 0.39 was employed to trim reads after a quality drop below a mean of Q15 in a window of 5 nucleotides and keeping only filtered reads longer than 15 nucleotides (Bolger et al., 2014). Reads were aligned versus Ensembl mouse genome version mm39 (Ensembl release 109) with STAR 2.7.10a (Dobin et al., 2013). Alignments were filtered to remove: duplicates with Picard 3.0.0 (Picard: A set of tools (in Java) for working with next generation sequencing data in the BAM format; http://broadinstitute.github.io/picard/), multi-mapping, ribosomal, or mitochondrial reads. Gene counts were established with featureCounts 2.0.4 by aggregating reads overlapping exons on the correct strand excluding those overlapping multiple genes (Liao et al., 2014). The raw count matrix was normalized with DESeq2 version 1.36.0 (Love et al., 2014). Contrasts were created with DESeq2 based on the raw count matrix. Genes were classified as significantly differentially expressed at average count > 5, multiple testing adjusted *p*-value < 0.05, and -0.585 < log2FC > 0.585. The Ensemble annotation was enriched with UniProt data (Activities at the Universal Protein Resource (UniProt)).

GSEA analysis

For gene set enrichment analysis, we used the GSEA tool (https://www.gsea-msigdb.org/gsea/index.jsp) and the Msigdb mouse H collection. We ran 1000 permutations on the ranked bulk RNA dataset (*FC* x (log10(*pvalue*) x (-1))). We excluded gene sets larger than 800 genes and small than 10 genes. Plotting was performed by python matplotlib.pyplot.scatter function.

scRNA-seq analyses

We used the public dataset (Negretti et al., 2021) provided by the Sucre Lab for our analysis. For the analysis of the endothelial cells, we extracted cells from the time points E12, E15, E16, E18, P0, and P3. Normalization, PCA, dimensionality reduction and clustering was done using the Scanpy (Wolf et al., 2018) framework and custom scripts. Of note, we did not perform a batch correction on individual samples. We isolated cluster 1 (containing the majority of E16 cells) and cluster 3 (containing the majority of E18 cells) and utilized Scanpy's (*rank_genes_groups*) function to determine the differentially expressed genes between the two clusters.
For epithelial analysis, we excluded Ciliated, Secretory, and Neuroendocrine clusters from the analysis. To score for the SPARCL1-induced NF-κB signature genes, we used Scanpy's (*score_genes*) function. The resulting score was used to split cells into "NF-kB+" and "NF-kB-" subpopulations and plotting (Figures 4B, S4A, S4B, S4D). Trajectory and pseudo-time analysis in Figure S4C was performed using scFates (Faure et al., 2023) with (*method="ppt", Nodes=50, ppt_lambda=200, ppt_sigma=0.6*). The proportion analysis for both datasets was done

using the tool Scanpro (Alayoubi et al., 2024), utilizing real and pseudo-replicates. *p* values from Scanpro are from empirical Bayes moderated ANOVA test.

For Figures S4E-S4J, the raw data were downloaded from GEO: GSE158192. We only used control samples for our analysis (Aggregate12files_control) (Hassan and Chen, 2024). The subsequent analysis was carried out using the sc_framework environment (Schultheis et al. (2024), https://zenodo.org/doi/10.5281/zenodo.11065517). We sub-setted the dataset to only include time points E16.5, E18.5, P7 and 15wk. QC included doublet detection and filtering by Scrublet (Wolock et al., 2019). All cells with less than 200 and more than 4000 genes, as well as cells with more than 35% ribosomal gene counts were filtered out. Further, cells with a high mitochondrial content (>10%) were discarded. This initial cell filtering resulted in a dataset with 20374 cells and 18619 genes. Counts per cell were normalized to the median count over all cells and transformed into log space to stabilize variance. The cell cycle for each cell was predicted based on predefined marker gene lists.

For (sub)clustering of *Cdh1* lineage cells, we used principal component analysis (PCA) for dimensionality reduction and included the first 35 components. We then calculated a neighbor graph (15 neighbors) and used UMAP for two dimensional embedding. To annotate cells, we used the "lineage" information provided by the original publication (Hassan and Chen, 2024), which divided the cells into epithelial, immune, mesenchymal, and endothelial clusters based on the expression of *Cdh1*, *Ptprc*, *Col3a1*, and *Icam2* respectively. After lineage annotation, we isolated epithelial (*Cdh1*+) cells and ran PCA, UMAP embedding and clustering separately as described before. To remove possible contamination, we retained only cells that express *Nkx2-1* (marker for epithelial cells) and removed all cells expressing *Vim* (marker for mesenchymal cells). We further subsetted the dataset to AT1, AT2, Sox9 progenitors and prolif. AT2 cells using the labels from the original publication, resulting in a dataset with 3474 cells. We calculated the mean expression of SPARCL1-induced NF-κB signature genes (*Ccl2*, *Tnfaip2*, *Cxcl5*, *Cxcl1*, *Lcn2*, *Adora1*, *Slc5a1*, *Ccl20*, *Mab21l3*, *Vnn3*, *Tnfaip3*, *Chil1*, *Cxcl2*, *Steap4*, *Muc4*, *Fcer2a*, *Lyz1*, *Scd1*, *Nfkbiz*, *Col6a4*, *Parm1*, *Saa3*, *Cftr*, *Zc3h12a*, *C3*, *Cd83*) in each time point and plotted them in the UMAP embedding as well as a violin plot, comparing the mean expression in AT1 and AT2 cells.

Phosphatidylcholine uptake and acidic organelle staining

Assays for the uptake of neutral lipids and acidic organelle staining were performed as described in (Chiu et al., 2022). Briefly, organoids were cultured for 24 h in the presence of 1 μM β-BODIPY FL C12-HPC (Invitrogen D3792). After washing with culture medium, organoids were stained using 100 nM LysoTracker Deep Red (Invitrogen L12492) and Hoechst 33342 (Invitrogen H3570) for 30 min at room temperature immediately followed by imaging.

Transmission Electron Microscopy

The organoids were fixed in 2% PFA and 2.5% glutaraldehyde in 0.1 M sodium cacodylate buffer (pH 7.4) for 2 h at room temperature, and then stored at 4°C overnight. Samples were washed in 0.1 M sodium cacodylate buffer and postfixed in 2% (w/v) OsO_4, followed by *en bloc* staining with 2% uranyl acetate. Samples were dehydrated with a graded series of washes in ethanol, transferred to ethanol/Epon solutions, and eventually embedded in Epon. Ultra-thin sections (approximately 60 nm thick) obtained with a Leica UC7 microtome were collected on copper slot grids with a pioloform support film. Sections were post-stained with uranyl acetate and lead citrate. Sections were examined with a Jeol JEM-1400 Plus transmission electron microscope (Jeol, Japan), operated at an accelerating

voltage of 120 kV. Digital images were recorded with an EM-14800 Ruby Digital CCD camera unit (3296 px x 2472 px).

SUPPLEMENTAL REFERENCES

Alayoubi, Y., Bentsen, M., and Looso, M. (2024). Scanpro is a tool for robust proportion analysis of single-cell resolution data. Sci Rep *14*, 15581. https://doi.org/10.1038/s41598-024-66381-7.

Beauchemin, K.J., Wells, J.M., Kho, A.T., Philip, V.M., Kamir, D., Kohane, I.S., Graber, J.H., and Bult, C.J. (2016). Temporal dynamics of the developing lung transcriptome in three common inbred strains of laboratory mice reveals multiple stages of postnatal alveolar development. PeerJ *4*, e2318. https://doi.org/10.7717/peerj.2318.

Bolger, A.M., Lohse, M., and Usadel, B. (2014). Trimmomatic: a flexible trimmer for Illumina sequence data. Bioinformatics *30*, 2114–2120. https://doi.org/10.1093/bioinformatics/btu170.

Chiu, M.C., Li, C., Liu, X., Yu, Y., Huang, J., Wan, Z., Xiao, D., Chu, H., Cai, J.-P., Zhou, B., et al. (2022). A bipotential organoid model of respiratory epithelium recapitulates high infectivity of SARS-CoV-2 Omicron variant. Cell Discov *8*, 1–15. https://doi.org/10.1038/s41421-022-00422-1.

Daniel, E., Azizoglu, D.B., Ryan, A.R., Walji, T.A., Chaney, C.P., Sutton, G.I., Carroll, T.J., Marciano, D.K., and Cleaver, O. (2018). Spatiotemporal heterogeneity and patterning of developing renal blood vessels. Angiogenesis *21*, 617–634. https://doi.org/10.1007/s10456-018-9612-y.

Dobin, A., Davis, C.A., Schlesinger, F., Drenkow, J., Zaleski, C., Jha, S., Batut, P., Chaisson, M., and Gingeras, T.R. (2013). STAR: Ultrafast universal RNA-seq aligner. Bioinformatics *29*, 15–21. https://doi.org/10.1093/bioinformatics/bts635.

Faure, L., Soldatov, R., Kharchenko, P.V., and Adameyko, I. (2023). scFates: a scalable python package for advanced pseudotime and bifurcation analysis from single-cell data. Bioinformatics *39*, btac746. https://doi.org/10.1093/bioinformatics/btac746.

Gillich, A., Zhang, F., Farmer, C.G., Travaglini, K.J., Tan, S.Y., Gu, M., Zhou, B., Feinstein, J.A., Krasnow, M.A., and Metzger, R.J. (2020). Capillary cell-type specialization in the alveolus. Nature *586*, 785–789. https://doi.org/10.1038/s41586-020-2822-7.

Hassan, D., and Chen, J. (2024). CEBPA restricts alveolar type 2 cell plasticity during development and injury-repair. Nat Commun *15*, 4148. https://doi.org/10.1038/s41467-024-48632-3.

Liao, Y., Smyth, G.K., and Shi, W. (2014). featureCounts: an efficient general purpose program for assigning sequence reads to genomic features. Bioinformatics *30*, 923–930. https://doi.org/10.1093/bioinformatics/btt656.

Liu, B., Yi, D., Yu, Z., Pan, J., Ramirez, K., Li, S., Wang, T., Glembotski, C.C., Fallon, M.B., Oh, S.P., et al. (2022). TMEM100, a Lung-Specific Endothelium Gene. Arteriosclerosis, Thrombosis, and Vascular Biology *42*, 1495–1497. https://doi.org/10.1161/ATVBAHA.122.317683.

Love, M.I., Huber, W., and Anders, S. (2014). Moderated estimation of fold change and dispersion for RNA-seq data with DESeq2. Genome Biology *15*, 550. https://doi.org/10.1186/s13059-014-0550-8.

Negretti, N.M., Plosa, E.J., Benjamin, J.T., Schuler, B.A., Habermann, A.C., Jetter, C.S., Gulleman, P., Bunn, C., Hackett, A.N., Ransom, M., et al. (2021). A single-cell atlas of mouse lung development. Development *148*, dev199512. https://doi.org/10.1242/dev.199512.

Schultheis, H., Detleffsen, J., Wiegandt, R., Bentsen, M., Alayoubi, Y., Valente, G., Keßler, M.F., Heger, V., Khassafi, F., Talyan, S., et al. (2024). loosolab/SC-Framework: Version 0.7 (Zenodo).

Shin, H., Morty, R.E., Sucre, J.M., Negretti, N.M., Markmann, M., Hossain, H., Krauss-Etschmann, S., Dehmel, S., and Hilgendorff, A. (2022). Reference genes for the developing mouse lung under consideration of biological, technical and experimental confounders. Sci Rep *12*, 17679. https://doi.org/10.1038/s41598-022-19071-1.

Wolf, F.A., Angerer, P., and Theis, F.J. (2018). SCANPY: large-scale single-cell gene expression data analysis. Genome Biology *19*, 15. https://doi.org/10.1186/s13059-017-1382-0.

Wolock, S.L., Lopez, R., and Klein, A.M. (2019). Scrublet: Computational Identification of Cell Doublets in Single-Cell Transcriptomic Data. Cell Systems *8*, 281-291.e9. https://doi.org/10.1016/j.cels.2018.11.005.
