# Supplementary material for: The lung microvasculature promotes alveolar type 2 cell differentiation via secreted SPARCL1
Source: Stem Cell Reports. 2025 Mar 20;20(4):102451. doi: 10.1016/j.stemcr.2025.102451 (PMC12069885; doi:10.1016/j.stemcr.2025.102451)
Supplement: Document S2. Article plus supplemental information [file mmc5.pdf]

Stem Cell Reports
Article
OPEN ACCESS

The lung microvasculature promotes alveolar type 2 cell differentiation via secreted SPARCL1

Paolo Panza,[1,2,7,8,12,*] Hyun-Taek Kim,[1,9,11] Till Lautenschläger,[1,10,11] Janett Piesker,[3] Stefan Günther,[4] Yousef Alayoubi,[5] Ondine Cleaver,[6] Mario Looso,[5] and Didier Y.R. Stainier[1,7,8,*]
[1]Department of Developmental Genetics, Max Planck Institute for Heart and Lung Research, Bad Nauheim, Germany
[2]Department of Medicine V, Internal Medicine, Infectious Diseases and Infection Control, Justus-Liebig University Giessen, Giessen, Germany
[3]Scientific Service Group Microscopy, Max Planck Institute for Heart and Lung Research, Bad Nauheim, Germany
[4]Deep Sequencing Platform, Max Planck Institute for Heart and Lung Research, Bad Nauheim, Germany
[5]Bioinformatics Core Unit, Max Planck Institute for Heart and Lung Research, Bad Nauheim, Germany
[6]UT Southwestern Medical Center, Dallas, TX, USA
[7]Member of the German Center for Lung Research, DZL-UGMLC
[8]Member of the Excellence Cluster Cardio-Pulmonary Institute, CPI
[9]Present address: Soonchunhyang Institute of Medi-bio Science, Cheonan, Republic of Korea
[10]Present address: Institute for Cardiovascular Regeneration, Goethe University Frankfurt, Frankfurt am Main, Germany
[11]These authors contributed equally
[12]Lead contact
*Correspondence: paolo.panza@mpi-bn.mpg.de (P.P.), didier.stainier@mpi-bn.mpg.de (D.Y.R.S.)
https://doi.org/10.1016/j.stemcr.2025.102451

SUMMARY

Lung endothelial cells (ECs) and pericytes are closely juxtaposed with the respiratory epithelium before birth and thus may have instructive roles during development. To test this hypothesis, we screened EC-secreted proteins for their ability to alter cell differentiation in alveolar organoids. We identified secreted protein acidic and rich in cysteine-like protein 1 (SPARCL1) as an extracellular matrix molecule that can promote alveolar type 2 (AT2) cell differentiation *in vitro*. SPARCL1-treated organoids display *lysozyme* upregulation and a doubling in the number of AT2 cells at the expense of intermediate progenitors. SPARCL1 also induces the upregulation of nuclear factor κB (NF-κB) target genes, and suppression of NF-κB activation in lung organoids blocked SPARCL1 effects. NF-κB activation by lipopolysaccharide (LPS) was sufficient to induce AT2 cell differentiation; however, pharmacological inhibition of the pathway alone did not prevent it. These data support a role for SPARCL1 and NF-κB in alveolar cell differentiation and suggest a potential value in targeting this signaling axis to promote alveolar maturation and regeneration.

INTRODUCTION

At late embryonic stages in the mouse (E16-E18), the branched fetal lung undergoes a dramatic morphogenetic transition into a meshwork of alveolar sacs. At the same time, alveolar epithelial progenitors differentiate into morphologically, functionally, and molecularly distinct respiratory cell types. Flat, thin, and elongated alveolar type 1 (AT1) cells cover the surface of pre-alveolar structures, while cuboidal alveolar type 2 (AT2) cells secrete surfactant.

Several models have been proposed to explain the molecular and mechanical diversification of alveolar epithelial cells (Treutlein et al., 2014; Li et al., 2018; Frank et al., 2019). However, it remains unclear how the differentiation of alveolar progenitor cells is controlled in space and time, and in particular whether and how specific interstitial cues influence the alveolar fate (Hogan, 2018).

AT2 cell differentiation is critical for surfactant production and immune protection, preventing respiratory distress at birth. Despite the physiological importance of AT2 cells, only a few extracellular molecules have proposed roles in inducing their differentiation. Mesenchymal cell-derived fibroblast growth factor (FGF) 10 acts via epithelial FGF receptor 2 (FGFR2) and drives the apical constriction of differentiating AT2 cells from the alveolar lumen (Li et al., 2018). Consistent with these data, FGF7 stimulation together with FGFR2 overexpression induces the AT2 fate in cultured E16.5 epithelial progenitors (Brownfield et al., 2022). Hepatocyte growth factor (HGF)/c-Met signaling has also been implicated in saccular morphogenesis (Yamamoto et al., 2007) and alveolar cell proliferation (Calvi et al., 2013; Kato et al., 2018). Besides these players, however, little is known about the molecular microenvironment provided by interstitial cells to drive alveolar cell differentiation.

The mature lung in both humans and mice is highly vascularized, with approximately 30% of all cells being endothelial cells (ECs) (Weibel, 1984). While the high density of blood vessels in the lung reflects its physiological role in gas exchange, it also suggests that vascular cells have an additional role(s) beyond blood circulation and gas exchange (Ramasamy et al., 2015).

In adult stages, the lung endothelium responds to injury by activating pathways for alveolar re-epithelialization, for example, by secreting MMP14 (Ding et al., 2011; Rafii et al., 2015), and thrombospondin-1 (Lee et al., 2014). During embryonic development, disrupting vascularization

© 2025 The Author(s).
Published by Elsevier Inc. on behalf of International Society for Stem Cell Research.
This is an open access article under the CC BY license (http://creativecommons.org/licenses/by/4.0/).

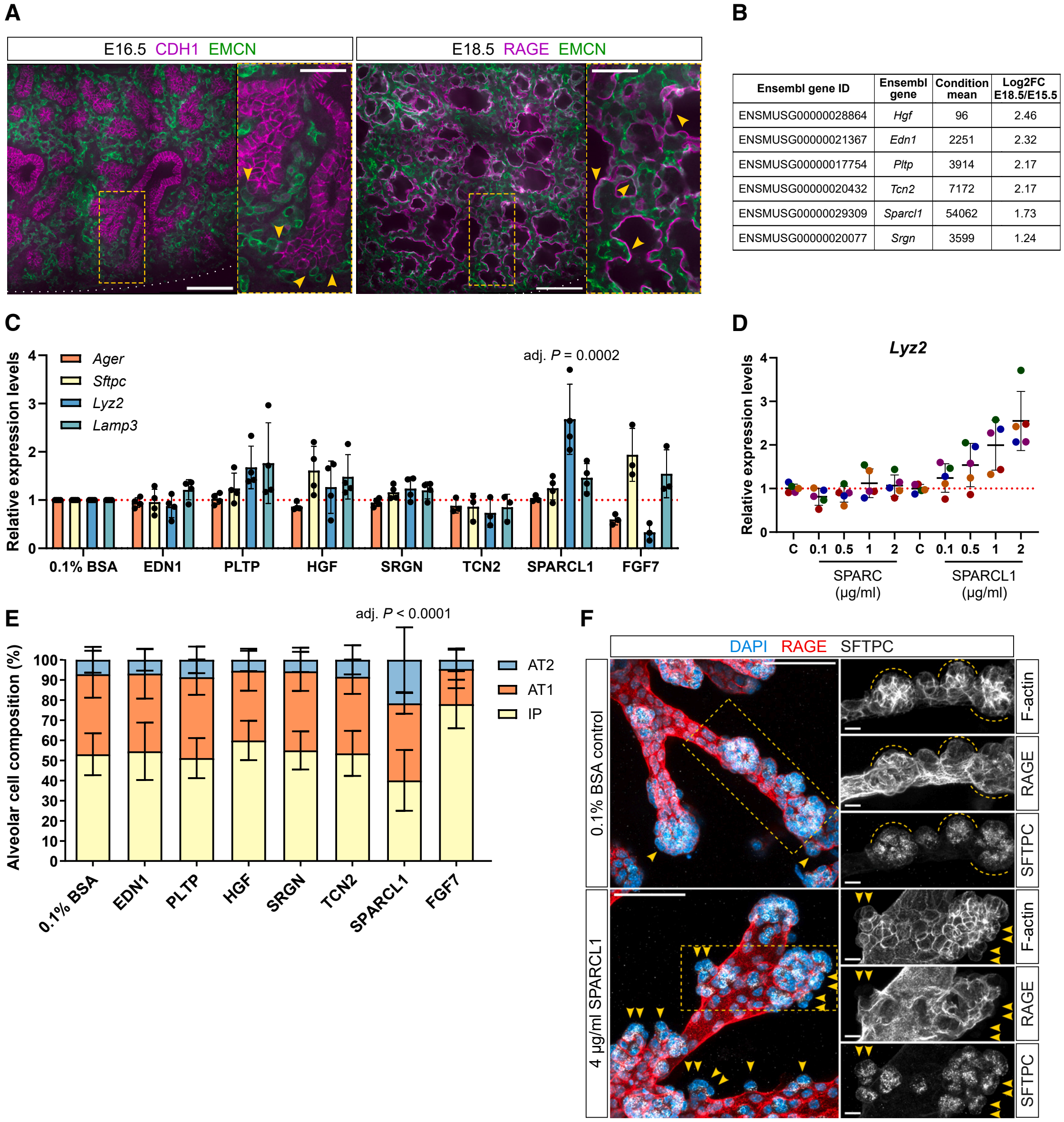

Ensembl gene ID	Ensembl gene	Condition mean	Log2FC E18.5/E15.5
ENSMUSG00000028864	*Hgf*	96	2.46
ENSMUSG00000021367	*Edn1*	2251	2.32
ENSMUSG00000017754	*Pltp*	3914	2.17
ENSMUSG00000020432	*Tcn2*	7172	2.17
ENSMUSG00000029309	*Sparcl1*	54062	1.73
ENSMUSG00000020077	*Srgn*	3599	1.24

Figure 1. SPARCL1 promotes AT2 cell differentiation in fetal lung organoids

(A) Endothelial cells (ECs) are closely juxtaposed with the alveolar epithelium in saccular lungs. Immunostaining of 150 μm precision-cut lung slices from E16.5 and E18.5 mouse embryos. CDH1 (magenta, epithelium), RAGE (magenta, basolateral membranes of AT1 cells), EMCN (green, ECs). Insets: high magnification of endothelial-epithelial contacts (yellow arrowheads). Scale bars: 100 μm, 50 μm (inset).

(B) Shortlist of candidate genes encoding secreted proteins. Candidates' expression levels at E18.5 compared with E15.5 in sorted (KDR^+) lung ECs (Daniel et al., 2018).

(C) Recombinant protein screen ($n \geq 3$). SPARCL1 treatments lead to an upregulation of *Lyz2*. FGF7 control treatments lead to an up-regulation of *Sftpc* (adj. $p = 0.0371$) and a downregulation of *Ager* and *Lyz2* (adj. $p = 0.0002$). Relative mRNA levels for markers of alveolar epithelial cell identity: *Ager* (AT1 and intermediate progenitor [IP] cells), *Sftpc* (AT2 and IP), *Lyz2* (AT2), and *Lamp3* (AT2). Data are presented as mean ± SD. p values are from one-way ANOVA, Tukey's multiple comparison testing.

(D) *Lyz2* mRNA levels increase dose dependently in organoids treated with SPARCL1, but not SPARC ($n = 5$ dams, at least 12 organoids per condition). Data are presented as mean ± SD.

(E) Quantification of alveolar cell proportions in recombinant protein-treated organoids ($n = 3$ dams, at least 6 organoids per condition). SPARCL1 induces an increase in AT2 cell counts. FGF7 control treatments lead to an increase in IP cell counts (adj. $p < 0.0001$). Data are presented as mean ± SD. p values are from one-way ANOVA, Tukey's multiple comparison testing.

(legend continued on next page)

ex vivo affects the stereotypical pattern of airway branching, consistent with a perfusion-independent crosstalk between the endothelium and epithelium (Lazarus et al., 2011). During late gestation and coinciding with lung sacculation, ECs begin to acquire organ-specific heterogeneity and start expressing distinct angiocrine factors (Gomez-Salinero et al., 2021).

Here, we address the molecular contribution of ECs and pericytes to a key event in alveologenesis, namely, the differentiation of distal airway progenitor cells into mature alveolar epithelial cells. By investigating the expression landscape of microvascular ECs in the mouse lung at late gestation, we focus on 6 candidate secreted molecules. Using an organoid model of alveolar cell differentiation (Gkatzis et al., 2021), we identify secreted protein acidic and rich in cysteine-like protein 1 (SPARCL1) as a factor that promotes AT2 cell differentiation via nuclear factor κB (NF-κB) signaling. SPARCL1/NF-κB promotes the expression of a subset of immune-related transcripts that are markers of mature AT2 cells, thereby completing their differentiation.

RESULTS

SPARCL1 promotes AT2 cell differentiation in lung organoids

During lung sacculation (E17-P4 in mouse), a first wave of cell differentiation takes place in the distal airway. To identify extra-epithelial modulators of AT1 and AT2 cell differentiation, we focused on ECs as a potential signal-producing cell population. ECs surround the terminal airway at E16, and starting at saccular stages, they come in close proximity with the alveolar lining (Figure 1A). We analyzed a multi-organ EC expression dataset of the fetal mouse (Daniel et al., 2018) and listed genes that (1) are expressed by lung ECs, (2) encode secreted proteins, and (3) are expressed at increasing levels between E15.5 and E18.5, correlating with alveolar morphogenesis (Table S1). Shortlisted candidates include *endothelin 1* (*Edn1*), *Hgf* (both previously described as angiocrine factor genes), *phospholipid transfer protein* (*Pltp*), *transcobalamin* 2 (*Tcn2*), *Sparcl1*, and *serglycin* (*Srgn*) (Figure 1B), and their expression pattern within the distal lung interstitium was verified by *in situ* hybridization (Figure S1A).

Next, to test whether the candidate proteins could affect alveolar epithelial cell differentiation, we generated alveolar organoids from freshly isolated E14.5 distal lung epithelial tips (Gkatzis et al., 2021) and screened recombinant proteins at a dose of 1 μg/mL from days 6 to 8 of culture. During this phase, in the absence of growth factors, 10%–20% of distally located cells in the organoids acquire differentiated AT1 or AT2 cell characteristics (Gkatzis et al., 2021). To identify which proteins could alter the number of differentiated cells in organoids, we first measured the mRNA levels of the alveolar cell marker genes *Ager*, *Sftpc*, *Lyz2*, and *Lamp3* by quantitative reverse-transcription PCR (RT-qPCR) (see supplemental information for details about these markers). Next, to determine the proportion of differentiated cells in the treated organoids, we performed immunostaining for pro-surfactant protein C (Pro-SFTPC, hereafter referred to as SFTPC) and advanced glycosylation end product-specific receptor (RAGE), which can distinguish between undifferentiated intermediate progenitor (IP: SFTPC$^+$/RAGE$^+$) and differentiated cell types (AT1: SFTPC$^-$/RAGE$^+$; AT2: SFTPC$^+$/RAGE$^-$) in the organoids (Gkatzis et al., 2021). Control treatments using 10 ng/μL FGF7 led to increased *Sftpc* and decreased *Ager* and *Lyz2* mRNA levels (Figure 1C). At the cellular level, FGF7-treated organoids were composed mostly of IP cells at the expense of AT1 cells, an effect that can be explained by the growth-promoting activity of FGF7, or/and by its ability to induce an intermediate state in AT1 cells (Figures 1E and S1B). These data, together with previous studies (Gkatzis et al., 2021), indicate that *Sftpc* mRNA levels correlate with the number of IP cells (about 50% in control treatments) in these alveolar organoids, whereas *Ager* and *Lyz2* mRNA levels correlate with the number of AT1 and AT2 cells (40% and 10%), respectively.

Upon HGF stimulation, the branching structures of organoids appeared shorter and stumpier compared with control (Figure S1B). This morphology corresponded with an increased proportion of IP cells, which however was not statistically significant (Figures 1E and S1B). Similar to FGF7, HGF treatments also led to a slight upregulation of *Sftpc* and *Lamp3*, but no change in *Ager* or *Lyz2* mRNA levels, together reflecting the higher proportion of IP cells (Figures 1C, 1E, and S1B). Taken together, FGF7 and HGF treatments lead to the expansion of the IP cell population, likely as a result of the growth-promoting activity of these factors. The high number of cells in an intermediate state, in turn, correlates with the poor elongation of epithelial branches (Figure S1B). These data further indicate that the fetal lung organoids offer a reliable readout of alveolar cell differentiation.

Among the screened factors, we found that recombinant SPARCL1 promoted AT2-like gene expression as shown by the strong increase in *Lyz2* mRNA levels, while no change

(F) Increased AT2 cell counts in SPARCL1-treated organoids. Maximum intensity projections of representative control and SPARCL1-treated organoids (4 μg/mL) immunostained for RAGE (red, AT1 cells) and SFTPC (white, AT2 cells). Increased numbers of SFTPC$^+$/RAGE$^-$ cells (yellow arrowheads) in SPARCL1-treated organoids. Insets: details of organoid branches. Scale bars: 50 μm, 10 μm (insets).

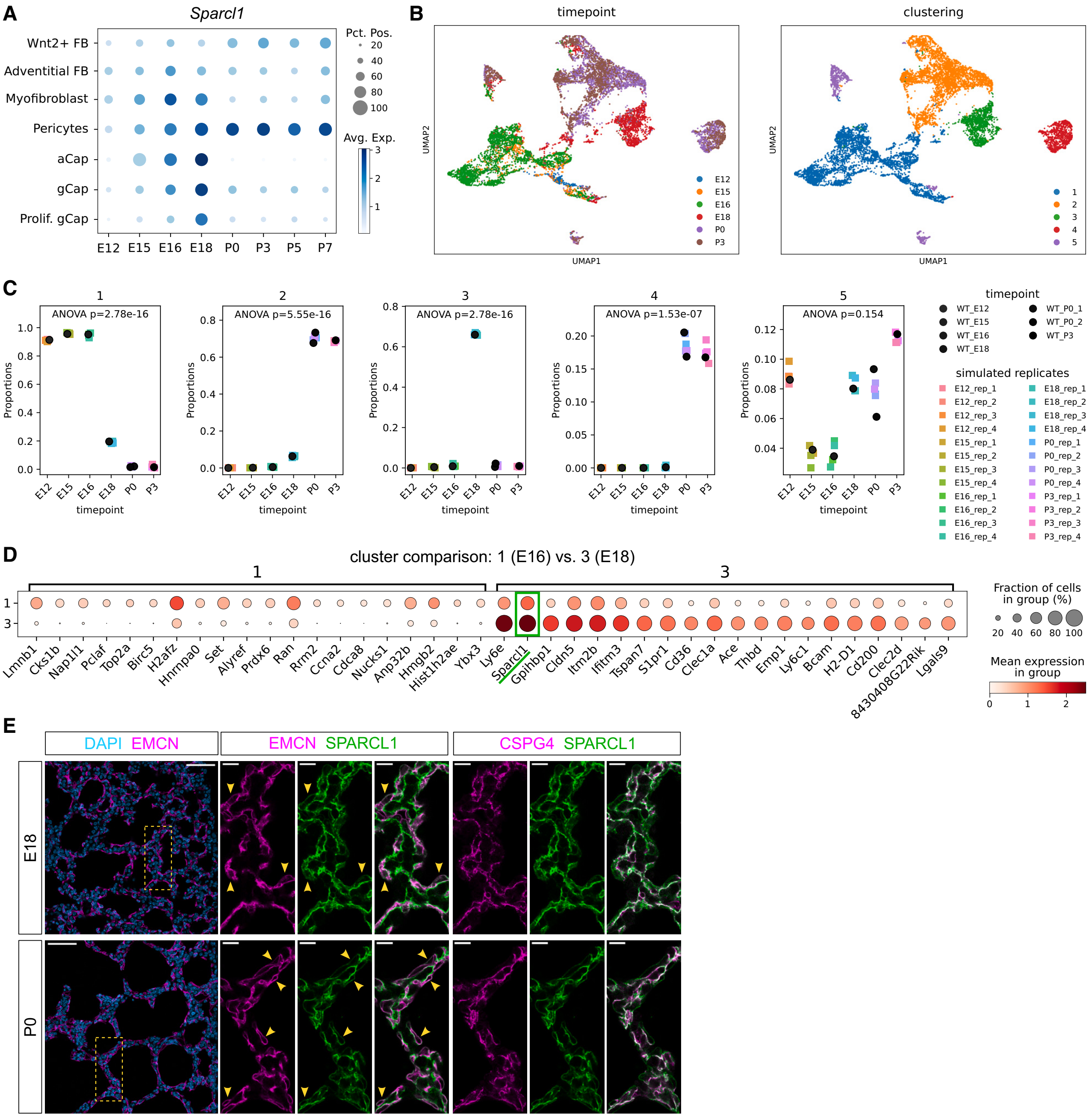

Figure 2. SPARCL1 is a marker of the E18 microvasculature

(A) *Sparcl1* expression peaks in lung capillary ECs at E18 and is maintained in pericytes postnatally. gCap, general capillary cell; aCap, alveolar capillary cell/aerocyte; data from Negretti et al., 2021.

(B) Leiden clustering of single lung EC transcriptomes from 6 developmental stages (E12-P3). Left: pre-saccular EC transcriptomes (blue, yellow, green) separate from postnatal ones (purple, brown) in the uniform manifold approximation and projection (UMAP) space. Right: cluster 3 (green) is distinct from cluster 1 (blue, pre-saccular ECs) and clusters 2 and 4 (yellow and red, postnatal ECs); data from Negretti et al., 2021.

(C) Cluster 3 ECs derive from the lungs at E18. Cluster identity of ECs quantified as proportion of all ECs profiled per developmental stage. Each scRNA-seq replicate is quantified independently. A majority of ECs profiled at E12, E15, and E16 are represented in cluster 1. Clusters 2 and 4 are populated by postnatal ECs. The majority of E18 ECs are in cluster 3, and a minority in clusters 1 and 2. *p* values are from empirical Bayes moderated ANOVA test.

(legend continued on next page)

was observed in *Sftpc* or *Ager* mRNA levels (Figure 1C). This transcriptional effect was dose dependent and was not observed when organoids were treated with equal concentrations of SPARC, a protein encoded by a *Sparcl1* paralog (Figures 1D and S1C).

Organoids treated with SPARCL1 exhibited a doubling of the number of AT2 cells at the expense of IP cells, while the proportion of AT1 cells remained comparable with controls (Figure 1E). Confirming these results, high-dose SPARCL1 treatments (4 μg/mL) led to higher numbers of AT2 cells in distal branches in lung organoids, as well as increased mRNA levels for mature AT2 marker genes (Figures 1F, S1D, and S1E). Most AT2 cells in these lung organoids bud outward and maintain limited access with the lumen, similar to observations *in vivo* (Li et al., 2018). They express abundant SFTPC within cytoplasmic organelles that are predominantly apical in localization. The basolateral expression of RAGE is absent in these cells compared with neighboring ones, indicating differentiation from the intermediate cell state (Figures 1F and S1F). These cells display features of functionally mature AT2 cells, including lamellar bodies (Figure S1G), and uptake labeled phosphatidylcholine (Figure S1H) consistent with previous observations (Chiu et al., 2022).

To determine whether SPARCL1 treatments influence alveolar cell proliferation in these lung organoids, we performed EdU incorporation in the presence of recombinant SPARCL1 for 24 and 48 h. We did not observe significant differences in the number or identity of the EdU^+ cells (Figure S2), suggesting that the increased number of AT2 cells is not due to increased proliferation.

In summary, our recombinant protein screen revealed that activation of growth factor signaling by FGF7 or HGF can inhibit organoid branch outgrowth and cell differentiation. Among the 6 secreted proteins tested, only SPARCL1 led to an increased number of mature AT2 cells, apparently by promoting their differentiation from IP cells and without affecting AT1 cell numbers.

SPARCL1 is a marker of the E18 lung microvasculature

SPARCL1, also known as Hevin, is a secreted glycoprotein that associates with the extracellular matrix (ECM) (Sullivan and Sage, 2004) and plays multiple roles in cell adhesion (Girard and Springer, 1996; Gongidi et al., 2004), synaptogenesis (Gan and Südhof, 2020; Kucukdereli et al., 2011; Singh et al., 2016), and EC quiescence (Naschberger et al., 2016; Regensburger et al., 2021). SPARCL1 is expressed in various tissues including the mammalian lung (Klingler et al., 2020). To verify the cellular source of SPARCL1 in the developing lung, we analyzed published single-cell RNA sequencing (scRNA-seq) datasets from late embryonic and early postnatal stages of lung development (Negretti et al., 2021). At E16, *Sparcl1* is broadly expressed by capillary ECs, pericytes, and myofibroblasts. By E18, *Sparcl1* is highly expressed in cell types constituting the lung microvasculature, i.e., capillary ECs and pericytes (Figure 2A). At postnatal stages, high *Sparcl1* expression is maintained in lung pericytes and is reduced in capillary ECs as well as in myofibroblasts (Figure 2A). These observations suggest that lung ECs transiently increase *Sparcl1* expression during late gestation, at a stage when AT2 cells first differentiate.

We next asked whether *Sparcl1*-expressing cells constitute a transcriptionally discrete EC subset. Leiden clustering of single-EC transcriptomes differentiated prenatal and postnatal EC groups and also highlighted a cluster predominantly composed of E18 ECs (cluster 3, Figure 2B). Analysis of the proportion of cluster identities revealed that about 60% of the profiled E18 ECs belong to cluster 3, while other stages were almost absent (Figure 2C). These data indicate that a population of E18 ECs is transcriptionally distinct from E16 and postnatal lung ECs. The comparison between the transcriptomes of clusters 1 and 3 revealed *Ly6e* and *Sparcl1* as the highest upregulated genes in cluster 3 (Figure 2D), suggesting that *Sparcl1* upregulation marks the emergence of this transitional EC subset. Cluster 3 ECs also express *Gpihbp1* (Figures 2D and S3A), a marker of general capillary (gCap) cells (Gillich et al., 2020). Among the differentially expressed genes, *Sparcl1* was the highest expressed secreted protein-encoding gene, suggesting that SPARCL1 secretion may define a signaling function of cluster 3 ECs.

Confirming these findings, immunostaining co-localized SPARCL1 with membranes of both ECs ($EMCN^+$) and pericytes ($CSPG4^+$, also known as NG2) in the distal lung at saccular stages (Figure 2E), including in ECs facing the alveolar surface, consistent with the aerocyte (alveolar capillary) cell population (Gillich et al., 2020). Alpha smooth muscle $actin^+$ ($ACTA2^+$) saccular myofibroblasts were also observed in the distal lung and accounted for a minority of SPARCL1-expressing cells (Figures S3C and S3D). Altogether, these data suggest that SPARCL1 is a marker of the late embryonic lung microvasculature, that its expression is developmentally regulated, and that it marks a transitory transcriptional state in lung capillary ECs.

(D) High levels of *Ly6e* and *Sparcl1* expression identify cluster 3 ECs. Top 15 differentially expressed genes between EC clusters 1 and 3.
(E) SPARCL1 (green) co-localizes with membranes of microvascular ECs (EMCN, magenta) and pericytes (CSPG4, magenta) in distal lungs. Immunostaining of E18 (top row) and P0 (bottom row) lung cryosections. Left: overview of ECs in distal lung regions. Scale bars: 50 μm (left), 10 μm (middle and right).

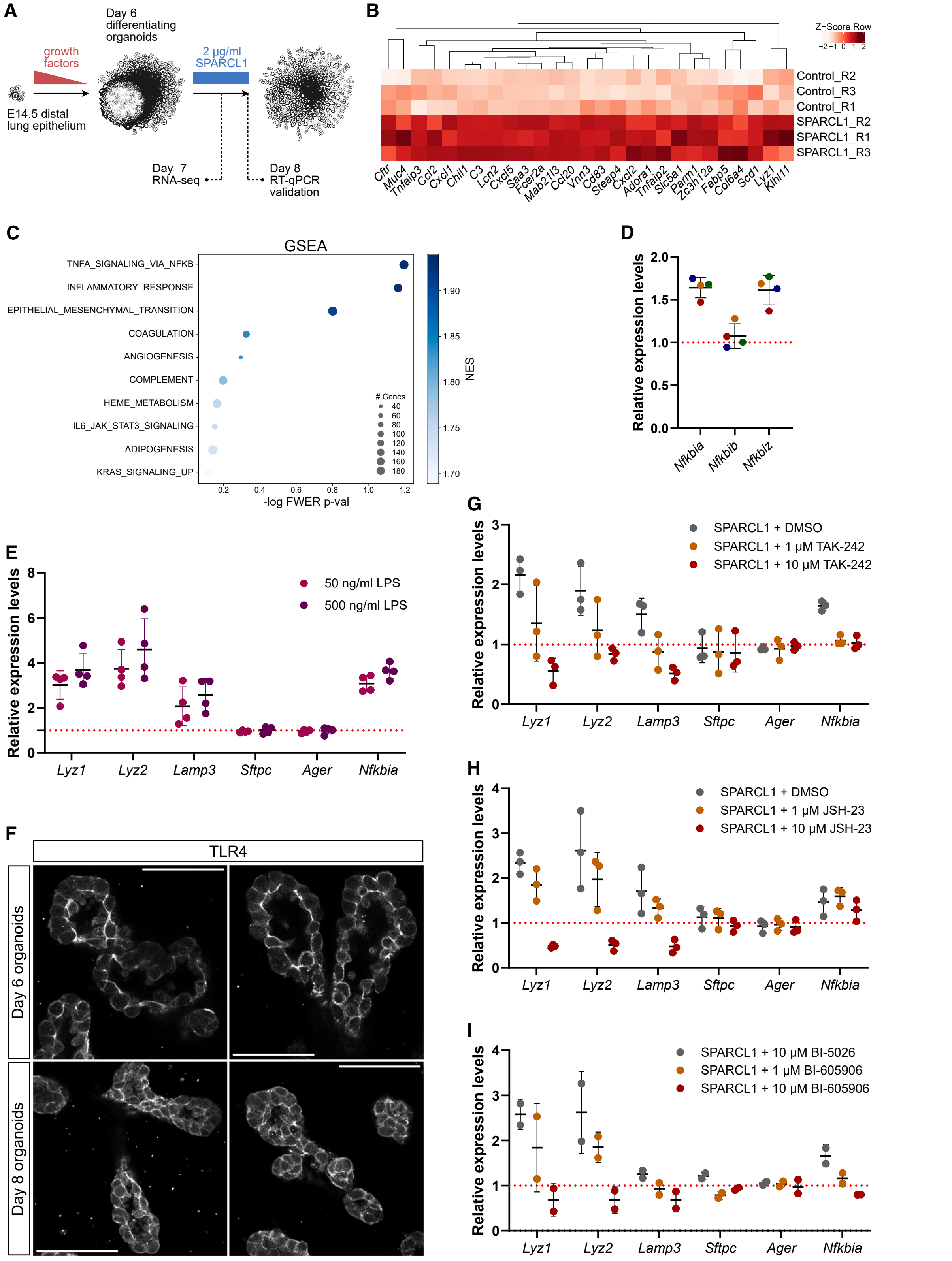

(legend on next page)

SPARCL1 activates NF-κB in alveolar epithelial cells

To identify the molecular mechanisms leading to AT2 cell differentiation, we profiled bulk transcriptomes of organoids treated with recombinant mouse SPARCL1 (2 μg/mL) for 24 h (Figure 3A). In this experiment, recombinant SPARCL1 was biologically active, as measured by the upregulation of *Lyz1*, *Lyz2*, and *Lamp3*, but not *Sftpc* or *Ager*, after 48 h (Figure S4A). The transcriptomic comparison between SPARCL1 and control treatments did not reveal widespread differences in gene expression, as the groups did not clearly segregate by principal component analysis (Figure S4B). However, a small set of genes were significantly upregulated in SPARCL1-treated organoids, including markers of functionally mature AT2 cells (*Chil1*, *Lcn2*, *Scd1*, and *Lyz1*) (Figure 3B).

Functional enrichment by gene set enrichment analysis (Subramanian et al., 2005) showed a significant correlation with TNFA signaling via NF-κB, inflammatory response, and epithelial-mesenchymal transition (Figure 3C), suggesting that the cellular response to SPARCL1 is mediated by TNFA/NF-κB signaling. To determine the responsiveness of known NF-κB target genes to SPARCL1, we measured the expression level of *Nfkbia*, *Nfkbib*, and *Nfkbiz*, all NF-κB transcriptional targets, and all encoding IkB-family negative feedback regulators of NF-κB activity (Oeckinghaus and Ghosh, 2009). These genes are all widely expressed in the mouse lung between E16 and P0 (Figure S4C, Negretti et al., 2021). *Nfkbia* and *Nfkbiz* expression levels increase progressively in the lung at late gestation, whereas *Nfkbib* does not (Figure S4D, Beauchemin et al., 2016). Congruent with these observations, we observed an increase in *Nfkbia* and *Nfkbiz* expression levels upon SPARCL1 treatment (Figure 3D), whereas *Nfkbib* was not regulated in organoids, suggesting that SPARCL1 activates NF-κB-mediated inflammatory signaling.

To test whether activation of NF-κB signaling was sufficient to stimulate the transcription of AT2 genes in alveolar organoids, we used bacterial lipopolysaccharide (LPS) at 50 and 500 ng/mL. LPS treatments did not lead to the upregulation of the alveolar epithelial marker genes that are also expressed in IP cells, such as *Sftpc* and *Ager*. However, the expression levels of *Lyz1*, *Lyz2*, and *Lamp3* were strongly increased, as well as those of a transcriptional target of NF-κB, *Nfkbia* (Figure 3E). These data suggest that NF-κB activation induces a specific transcriptional response in alveolar epithelial cells, similar to that observed in SPARCL1-treated organoids.

The main receptor for extracellular LPS is a protein complex formed by cluster of differentiation 14 (CD14), Toll-like receptor 4 (TLR4), and lymphocyte antigen 96 (LY96, also known as MD-2). We observed TLR4 expression on the plasma membrane of the majority of alveolar cells in organoids at days 6 and 8 of culture (Figure 3F), suggesting that TLR4 may act as a SPARCL1 receptor in alveolar epithelial cells, possibly similar to its role in hepatocytes (Liu et al., 2021). To test whether SPARCL1 signals are transduced through the TLR4-dependent LPS-sensing pathway, we stimulated organoids using SPARCL1 and simultaneously pharmacologically inhibited TLR4 receptor activation using TAK-242. TAK-242 is a competitive inhibitor of

Figure 3. SPARCL1 triggers AT2 cell differentiation via TLR4 and NF-κB activation in lung organoids

(A) Workflow schematic for RNA-seq of organoids. Control and SPARCL1-treated (2 μg/mL) organoids ($n = 3$ dams, at least 16 organoids per condition) were collected after 24 h (RNA-seq) and 48 h (RT-qPCR to validate recombinant protein activity).

(B) Upregulated genes in SPARCL1-treated organoids compared with control (24 h). Heatmap showing SPARCL1-regulated genes (adj. $p < 0.2$).

(C) The SPARCL1-induced gene signature is enriched in NF-κB pathway and inflammatory signaling genes. GSEA hallmark collections by high normalized enrichment score (NES): TNFA signaling via NF-κB (NES: 1.94), inflammatory response (NES: 1.94), epithelial mesenchymal transition (NES: 1.92). GSEA, gene set enrichment analysis; FWER, family-wise error rate.

(D) mRNA levels for *Nfkbia* and *Nfkbiz*, but not for *Nfkbib*, are increased in SPARCL1-treated organoids (48 h, $n = 4$ dams, at least 16 organoids per condition). Data are presented as mean ± SD.

(E) Bacterial LPS mimics SPARCL1 transcriptional effects in lung organoids. mRNA levels for the mature AT2 marker genes *Lyz1*, *Lyz2*, and *Lamp3*, as well as for *Nfkbia*, are increased by LPS treatment ($n = 4$ dams, at least 12 organoids per condition). *Sftpc* and *Ager* mRNA levels remained unchanged. Data are presented as mean ± SD.

(F) $TLR4^+$ alveolar epithelial cells in lung organoids on culture days 6 and 8, in areas of active cell differentiation. Scale bars: 50 μm.

(G) Pharmacological inhibition of TLR4 blocks SPARCL1 transcriptional effects in lung organoids. 1 μM TAK-242 reduced and 10 μM TAK-242 blunted the SPARCL1-induced upregulation of *Lyz1*, *Lyz2*, and *Lamp3*, as well as of *Nfkbia* ($n = 3$ dams, at least 12 organoids per condition). *Sftpc* and *Ager* mRNA levels remained unchanged. Data are presented as mean ± SD.

(H) Pharmacological inhibition of RELA nuclear translocation blocks SPARCL1 transcriptional effects in lung organoids. 10 μM JSH-23 profoundly reduced *Lyz1*, *Lyz2*, and *Lamp3* mRNA levels ($n = 3$ dams, at least 11 organoids per condition). *Sftpc* and *Ager* mRNA levels remained unchanged. *Nfkbia* expression did not change significantly upon JSH-23 treatment. Data are presented as mean ± SD.

(I) Pharmacological inhibition of IKKβ blocks SPARCL1 transcriptional effects in lung organoids. 10 μM BI-605906 blunted the SPARCL1-induced upregulation of *Lyz1*, *Lyz2*, and *Lamp3*, as well as of *Nfkbia* ($n = 2$ dams, at least 12 organoids per condition). Controls were treated with the inactive and structurally similar compound BI-5026. Data are presented as mean ± SD.

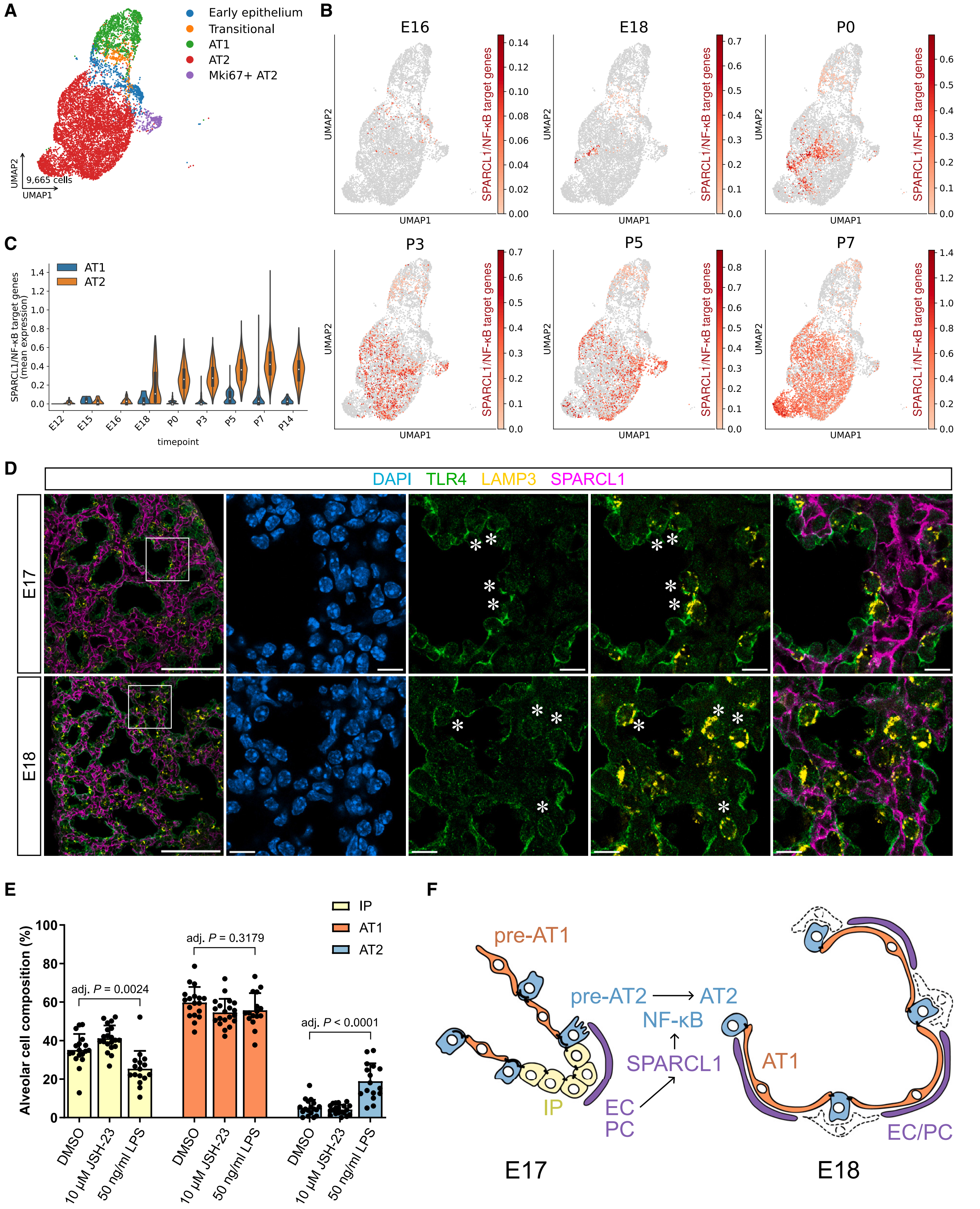

(legend on next page)

TLR4 downstream signaling and disrupts the interactions between the intracellular domain of TLR4 and adapter molecules (Ii et al., 2006). Similar to previous experiments, SPARCL1 treatments led to increased levels of *Lyz1*, *Lyz2*, and *Lamp3* mRNAs. This effect was reduced by TLR4 inhibition (1 μM), and 10 μM TAK-242 abolished SPARCL1 effects. 1 and 10 μM TAK-242 also prevented the increase in *Nfkbia* mRNA levels induced by SPARCL1 (Figure 3G).

To address the role of NF-κB as a transcriptional mediator of the response to extracellular SPARCL1, we treated organoids with SPARCL1 and simultaneously inhibited the nuclear translocation of the NF-κB transcription factor subunit RELA (also known as P65) by using the small molecule JSH-23 (Shin et al., 2004). Whereas treatments using 1 μM JSH-23 only mildly reduced the SPARCL1 effect on *Lyz1*, *Lyz2*, and *Lamp3* mRNA levels, a 10 μM dose downregulated these genes to 50% of control levels (Figure 3H). However, JSH-23 treatments failed to normalize *Nfkbia* mRNA levels, suggesting a complex regulation of this pathway in epithelial cells. Altogether, these results suggest that transcription of a subset of mature AT2 cell marker genes is NF-κB dependent. This interpretation was further supported by experiments interfering with NF-κB activation using BI-605906, an IKKβ inhibitor, which led to similar results to TAK-242 and JSH-23 (Figure 3I).

Thus, we propose that SPARCL1 signal transduction in alveolar epithelial cells depends on TLR4 receptor activation and RELA nuclear translocation and transcriptional activity.

Expression of SPARCL1/NF-κB targets marks mature AT2 cells, and NF-κB activation is sufficient for their differentiation *in vitro*

To determine whether lung epithelial cells transcribe SPARCL1/NF-κB target genes at saccular stages, we interrogated available scRNA-seq data from the developing mouse lung (Negretti et al., 2021; Figure 4A). E16 alveolar epithelial cells only weakly express SPARCL1-responsive genes (Figure 4B, Table S2). Starting from E18, however, an increasing number of epithelial cells express this gene signature, culminating at P7 (Figures 4B and S5D). The identified cells belong to the AT2 cell cluster, and the mean expression level of the query signature of SPARCL/NF-κB targets increased in AT2 cells starting at E18, compared with low expression in AT1 cells at corresponding developmental stages (Figure 4C). These data suggest that AT2 cells express NF-κB target genes beginning at E18, correlating with high levels of vascular SPARCL1 expression. The expression of SPARCL1/NF-κB targets is maintained in AT2 cells from 15-week-old mice (Figures S5G–S5J; Hassan and Chen, 2024), suggesting that SPARCL1 might enhance AT2 cell maturation. At all stages examined, AT1 cells lowly express SPARCL1/NF-κB target genes, indicating that NF-κB activation is specific to AT2 cells.

To determine whether NF-κB pathway components are expressed in the lung epithelium, we performed immunostaining at stages when early saccular morphogenesis and alveolar cell differentiation take place. At E17 and E18, TLR4 is broadly expressed in the lung and enriched in epithelial cells. In particular, TLR4 expression marks the membrane of numerous differentiating AT2 cells ($LAMP3^+$), although not exclusively. We observed basolateral expression of TLR4, in notable proximity to $SPARCL1^+$ cellular membranes (Figure 4D), confirming the physical proximity of differentiating AT2 cells with the SPARCL1 expression domain, and indicate that AT2 cells have the ability to transduce NF-κB-activating extracellular signals through TLR4.

JSH-23 treatments led to a profound reduction of *Lyz1*, *Lyz2*, and *Lamp3* mRNA levels compared with controls (Figure 3H), suggesting that NF-κB inhibition might

Figure 4. NF-κB target gene transcription correlates with AT2 cell maturity, and NF-κB activation is sufficient for AT2 cell differentiation in organoids

(A) Transcriptional diversity of alveolar epithelial cells in developing mouse lungs (E12-P14). scRNA-seq data and cell type annotation from Negretti et al., 2021. Red: AT2 cells; green: AT1 cells.

(B) SPARCL1/NF-κB target gene expression maps to AT2 cells beginning at E18. Mean expression levels for the SPARCL1/NF-κB target genes in alveolar epithelial cells profiled at E16-P7.

(C) Stage-resolved comparison of the mean expression level for SPARCL1/NF-κB target genes between AT1 and AT2 cells.

(D) TLR4 (green) is localized on the plasma membrane of distal airway epithelial cells at early saccular stages and marks the basolateral membrane of a subset of AT2 cells (asterisks; LAMP3, yellow). $TLR4^+$/$LAMP3^+$ cells are in contact with $SPARCL1^+$ membranes (magenta). Immunostaining of E17 and E18 lung cryosections. Left: low-magnification overviews. Right: single channel and merged views. Scale bars: 100 μm (left), 10 μm (right).

(E) NF-κB signaling activation in organoids is sufficient for AT2 cell differentiation. LPS stimulation of organoids increased the proportion of AT2 cells (blue bars) and decreased the number of IP cells (yellow bars). JSH-23 treatment alone did not prevent AT2 cell differentiation (n = 2 dams, at least 6 organoids per condition). Data are presented as mean ± SD. p values are from one-way ANOVA, Tukey's multiple comparison testing.

(F) Proposed signaling model. SPARCL1 is secreted by lung endothelial cells (ECs, purple) and pericytes (PCs, purple) and promotes AT2 cell differentiation (blue) via TLR4 and NF-κB. Cells marked by dashed outlines represent FGF-expressing fibroblasts.

dominantly block AT2 cell differentiation in organoids. To determine whether NF-κB signaling via RELA is required for AT2 cell differentiation, we treated organoids using JSH-23 at a time point (culture day 6) when only about 2%–3% of the cells in culture are SFTPC^{+}/RAGE^{-} and therefore identified as AT2 cells (Gkatzis et al., 2021). JSH-23 treatment alone did not block AT2 cell differentiation (Figure 4E). In contrast, LPS treatment of lung organoids significantly increased the proportion of AT2 cells at the expense of IP cells (Figure 4E), altogether suggesting that NF-κB activation is sufficient but not necessary for AT2 cell differentiation *in vitro*.

In summary, we identified SPARCL1 as a lung microvascular-secreted factor promoting AT2 cell differentiation in organoids. Transcriptomic and pharmacological evidence supports a role for SPARCL1-TLR4-NF-κB in the alveolar epithelium at late gestation. NF-κB-dependent transcription in alveolar cells, in turn, promotes the differentiation of AT2 cells and the acquisition of their immune physiological function.

DISCUSSION

A critical and unresolved question in the control of alveolar cell differentiation concerns the identity of the key signals as well as the underlying cellular and molecular mechanisms (including the crosstalk with niche cells) (Hogan, 2018). It remains unclear whether the process of alveolar cell differentiation depends on extrinsic signals besides FGFs, and organoids can help tease out intercellular communication in the lung (Gkatzis et al., 2018). Here, we provide evidence that signals from interstitial cells, and in particular ECs and pericytes, promote AT2 cell differentiation from the intermediate cell state.

In our recombinant protein screen, FGF7 treatments promoted an undifferentiated/intermediate cell state in organoids generated from E14.5 distal lung progenitor cells. In contrast, FGF7 induces AT1/AT2 cell differentiation of E16.5 progenitors (Brownfield et al., 2022). This discrepancy may arise from the different stage of tissue isolation (possibly reflecting a differential commitment status (Frank et al., 2019)), the different dose of FGF7 (10 and 50 ng/mL), and/or differential cell-non-autonomous effects from the ECM used.

Next, we identify SPARCL1 as an EC and pericyte-secreted factor that activates NF-κB in alveolar epithelial cells, in a TLR4 and RELA-dependent manner. SPARCL1-treated alveolar organoids increased the expression level of genes including *Lcn2*, *Lyz1*, *Scd1*, and *Fabp5*, all of which are markers of mature AT2 cells (Treutlein et al., 2014). Longer treatments led to upregulation of *Lyz2* and to a minor extent *Lamp3*, both also expressed in AT2 cells. These genes are involved in a set of immune functions that AT2 cells acquire before birth and maintain in the lung, including the production of surfactant and tubular myelin, and antimicrobial defense (Whitsett and Alenghat, 2015).

SPARCL1 induced AT2 cell differentiation in organoids without simultaneous FGF stimulation, suggesting that the SPARCL1 effect may be independent from an FGFR2-mediated fate selection mechanism (Brownfield et al., 2022). Together with our finding that NF-κB activation is sufficient but not necessary for AT2 cell differentiation in organoids, these observations support the co-existence of multiple pathways independently controlling alveolar cell differentiation.

In lung epithelial organoids, the early (24 h) transcriptional response to SPARCL1 was related to NF-κB signaling. These findings coincide with a described function of SPARCL1 as an extracellular NF-κB activator in the adult mouse liver (Liu et al., 2021) and lung (Zhao et al., 2024). SPARCL1 gain of function promoted inflammation in both organs. In the liver, SPARCL1 stimulated the expression of the cytokine genes *Ccl2*, *Cxcl1*, *Cxcl2*, and *Cxcl5* in hepatocytes, and, similarly, we identified these genes as SPARCL1 targets in our lung organoid model. Next, SPARCL1 co-immunoprecipitated with TLR4, suggesting that together they form a signal recognition complex at the plasma membrane (Liu et al., 2021). Consistent with this observation, we found that the epithelial response to SPARCL1 is TLR4 dependent. In adult mouse and human lungs, SPARCL1 is constitutively expressed in ECs and pericytes (Tabula Muris Consortium et al., 2018; The Tabula Sapiens Consortium, 2022), and *Sparcl1* mRNA levels increase in ECs following bleomycin administration in mice (Strunz et al., 2020). *Sparcl1* expression has been shown to increase in gCap cells upon influenza infection. SPARCL1 drives TLR4-mediated NF-κB activation in macrophages, inducing a phenotypic switch into the pro-inflammatory M1 type (Zhao et al., 2024). Beyond these data, the functional consequences of SPARCL1 activity on the lung alveolar epithelium had not been investigated.

Besides its major functions in immune cells, the NF-κB pathway has been shown to be activated in epithelial cells lining organ barriers with the external environment. In epithelial tissues, NF-κB has a conserved role in maintaining homeostasis and controlling inflammation (Pasparakis, 2012). In the epidermis, genetic data suggest a role for NF-κB in controlling epidermal cell proliferation (Seitz et al., 1998, 2000) and the propensity to differentiate (Kaufman and Fuchs, 2000; Hu et al., 2001; Pasparakis, 2012). In the gut epithelium, NF-κB activity is observed in Paneth cells, and IkBaΔN transgenic mice—in which NF-κB activity is dominantly suppressed (Schmidt-Ullrich et al., 2001)—display a reduction in LYZ2^{+} Paneth cell numbers and an

increase in goblet cell numbers (Brischetto et al., 2021), supporting a role for NF-κB in intestinal epithelial cell fate establishment. However, lung phenotypes in IkBaΔN mice have not been reported.

In the human lung, alveolar pneumocytes express functional TLR2 and TLR4 (Armstrong et al., 2004), two receptors that can activate NF-κB signaling. In mouse, intra-amniotic administration of LPS at E15 led to increased AT2 cell numbers at E18 (Prince et al., 2004), an effect that was not observed in *Tlr4*$^{Lps\text{-}d}$ mice, which display an attenuated response to LPS (Vogel et al., 1994). Altered numbers of AT2 cells have been identified in genetic manipulations of NF-κB signaling including the constitutive overexpression of RELA (Londhe et al., 2008) and the conditional deletion of the NF-κB-activating kinase IKKβ in lung epithelial cells (Londhe et al., 2011). These reports are aligned with our findings that NF-κB plays a direct role in AT2 cell differentiation.

We observed *Sparcl1* expression in ECs, pericytes, and myofibroblasts in the lung alveolar compartment of fetal mice. *Sparcl1* mRNA levels transiently increase in ECs between E16 and E18, consistent with reports of *Sparcl1* expression in E18.5 gCap ECs and its downregulation at postnatal stages (Zanini et al., 2023). Myofibroblasts are central players during secondary septation in the postnatal lung, but were found in low proportions in the distal region of E16-E18 lungs, supporting a primary role for microvascular ECs, pericytes, or both in alveolar cell differentiation, at least in part by secreting SPARCL1.

AT2 cell differentiation can be incomplete in the lung of very preterm neonates (Whitsett et al., 2015). Conditions of intra-amniotic infection can correlate with fetal lung damage and arrested development, as well as with increased lung maturity (Kramer et al., 2009). Similarly, the effects of manipulating NF-κB signaling in the developing lung vary (Alvira, 2014). In rhesus macaques, intra-amniotic administration of LPS at the end of the canalicular stage promoted fetal lung maturation and increased the number of AT2 cells. Transcriptomic analyses showed a negative regulation of cell proliferation and growth and an increase in genes associated with blood vessel development but also lamellar body formation (Schmidt et al., 2020). Combined with these data, our findings suggest that growth and specification of the mammalian lung vascular network is linked with AT2 cell differentiation and may be promoted by inflammatory stimuli.

In conclusion, by identifying SPARCL1 as an EC and pericyte-derived NF-κB activator promoting AT2 cell differentiation, as well as an NF-κB-dependent transcriptional program in AT2 cells, our study advances the understanding of the roles of NF-κB in lung development, with significant implications for both neonatal and chronic lung diseases.

METHODS

Fetal lung organoid cultures

All animal care and experimental procedures in this study were approved by the local animal ethics committee at the Regierungspräsidium Darmstadt, Hessen, Germany. Isolation and culture of freshly isolated fetal distal lung epithelial tissue were carried out as described in the study by Gkatzis et al. (2021). Replicates (n) correspond to tissue isolations from different pregnant dams.

Recombinant protein screen and chemical treatments

Purchased recombinant proteins (human EDN1: Reliatech 200-017S, mouse HGF: BioLegend 771604, human PLTP: Sino Biological 11171-H08H, mouse SPARCL1: Sino Biological 50544-M08H, human SRGN: Sino Biological 13648-H08H, mouse TCN2: Sino Biological 50693-M08H, and human FGF7: PeproTech 100-19) were diluted in sterile PBS containing 0.1% BSA (Sigma A1595). Recombinant proteins were used for screening at a concentration of 1 μg/mL in organoid medium without growth factors. Control conditions refer to treatments using isovolumes of 0.1% BSA in PBS. TAK-242/Resatorvid (Hycultec HY-11109), JSH-23 (Hycultec HY-13982), BI-605906, and BI-5026 (https://www.opnme.com/) were diluted in DMSO (Sigma D2650). LPS (Sigma L2630) was diluted in double distilled water.

Whole-mount organoid immunostaining

Organoid immunostaining was performed according to Gkatzis et al. (2021). At least 6 organoids from 3 wells per condition were quantified. Confocal images of distal regions in immunostained and live organoids were collected on a Zeiss CellDiscoverer 7 microscope, using a 50x Plan-APOCHROMAT 1.2× NA objective and 0.5× magnification.

Quantification of cell composition in organoids

Alveolar cell type composition was quantified according to methods described in Gkatzis et al. (2021). Cell counts are included in Data S3.

Quantification and statistical analysis

RT-qPCR data were normalized to values from control organoids (0.1% BSA). All results are expressed as mean values ± SD. p values are from one-way ANOVA, Tukey's multiple comparison testing.

RESOURCE AVAILABILITY

Lead contact

Requests for further information and resources should be directed to and will be fulfilled by the lead contact, Paolo Panza (paolo.panza@mpi-bn.mpg.de).

Materials availability

This study did not generate new unique reagents.

Data and code availability

The accession number for the RNA-seq data reported in this paper is GEO: GSE279892. RNA-seq data from sorted KDR$^+$ cells (Daniel et al., 2018) were kindly made available by the Cleaver lab. scRNA-seq data published by the Sucre lab (Negretti et al., 2021) were obtained from https://lungcells.app.vumc.org/ and from GEO: GSE165063. scRNA-seq data published by the Chen lab (Hassan and Chen, 2024) were obtained from GEO: GSE158192.

ACKNOWLEDGMENTS

We thank Jennifer Sucre for sharing the scRNA-seq datasets and for suggestions on data analysis, David Frank, Andrew Vaughan, Gan Zhao, Elisabeth Naschberger, and Michael Stürzl for sharing reagents, Lienhard Schmitz, Saverio Bellusci, and Chi-Chung Wu for discussions, Felix Gunawan and João Cardeira-da-Silva for critical reading of the manuscript, Kenny Mattonet and Radhan Ramadass for microscopy technical advice, Petra Neeb for technical help, Nouha Ritschel and the animal facility staff for excellent animal care, Petra Prückl for advice on animal welfare, and Simon Perathoner for scientific administrative support. BI-605906 and BI-5026 were kindly provided by Boehringer Ingelheim via its open innovation platform opnMe (https://www.opnme.com). This work was supported by a CPI flexible outbreak project grant awarded to P.P. and D.Y.R.S. and by funds from the Max Planck Society to D.Y.R.S.

AUTHOR CONTRIBUTIONS

P.P. and D.Y.R.S. designed research; P.P., H.-T.K., T.L., J.P., and S.G. performed research; P.P., H.-T.K., T.L., J.P., S.G., Y.A., O.C., and M.L. analyzed the data; and P.P. and D.Y.R.S. wrote the paper.

DECLARATION OF INTERESTS

The authors declare no competing interests.

SUPPLEMENTAL INFORMATION

Supplemental information can be found online at https://doi.org/10.1016/j.stemcr.2025.102451.

Received: May 21, 2024
Revised: February 15, 2025
Accepted: February 17, 2025
Published: March 20, 2025

REFERENCES

Alvira, C.M. (2014). Nuclear factor-kappa-B signaling in lung development and disease: One pathway, numerous functions. Birth Defects Res. A Clin. Mol. Teratol. *100*, 202–216. https://doi.org/10.1002/bdra.23233.

Armstrong, L., Medford, A.R.L., Uppington, K.M., Robertson, J., Witherden, I.R., Tetley, T.D., and Millar, A.B. (2004). Expression of Functional Toll-Like Receptor-2 and -4 on Alveolar Epithelial Cells. Am. J. Respir. Cell Mol. Biol. *31*, 241–245. https://doi.org/10.1165/rcmb.2004-0078OC.

Beauchemin, K.J., Wells, J.M., Kho, A.T., Philip, V.M., Kamir, D., Kohane, I.S., Graber, J.H., and Bult, C.J. (2016). Temporal dynamics of the developing lung transcriptome in three common inbred strains of laboratory mice reveals multiple stages of postnatal alveolar development. PeerJ *4*, e2318. https://doi.org/10.7717/peerj.2318.

Brischetto, C., Krieger, K., Klotz, C., Krahn, I., Kunz, S., Kolesnichenko, M., Mucka, P., Heuberger, J., Scheidereit, C., and Schmidt-Ullrich, R. (2021). NF-κB determines Paneth versus goblet cell fate decision in the small intestine. Development *148*, dev199683. https://doi.org/10.1242/dev.199683.

Brownfield, D.G., De Arce, A.D., Ghelfi, E., Gillich, A., Desai, T.J., and Krasnow, M.A. (2022). Alveolar cell fate selection and lifelong maintenance of AT2 cells by FGF signaling. Nat. Commun. *13*, 7137. https://doi.org/10.1038/s41467-022-34059-1.

Calvi, C., Podowski, M., Lopez-Mercado, A., Metzger, S., Misono, K., Malinina, A., Dikeman, D., Poonyagariyon, H., Ynalvez, L., Derakhshandeh, R., et al. (2013). Hepatocyte Growth Factor, a Determinant of Airspace Homeostasis in the Murine Lung. PLoS Genet. *9*, e1003228. https://doi.org/10.1371/journal.pgen.1003228.

Chiu, M.C., Li, C., Liu, X., Yu, Y., Huang, J., Wan, Z., Xiao, D., Chu, H., Cai, J.-P., Zhou, B., et al. (2022). A bipotential organoid model of respiratory epithelium recapitulates high infectivity of SARS-CoV-2 Omicron variant. Cell Discov. *8*, 57. https://doi.org/10.1038/s41421-022-00422-1.

Daniel, E., Azizoglu, D.B., Ryan, A.R., Walji, T.A., Chaney, C.P., Sutton, G.I., Carroll, T.J., Marciano, D.K., and Cleaver, O. (2018). Spatiotemporal heterogeneity and patterning of developing renal blood vessels. Angiogenesis *21*, 617–634. https://doi.org/10.1007/s10456-018-9612-y.

Ding, B.S., Nolan, D.J., Guo, P., Babazadeh, A.O., Cao, Z., Rosenwaks, Z., Crystal, R.G., Simons, M., Sato, T.N., Worgall, S., et al. (2011). Endothelial-derived angiocrine signals induce and sustain regenerative lung alveolarization. Cell *147*, 539–553. https://doi.org/10.1016/j.cell.2011.10.003.

Frank, D.B., Penkala, I.J., Zepp, J.A., Sivakumar, A., Linares-Saldana, R., Zacharias, W.J., Stolz, K.G., Pankin, J., Lu, M., Wang, Q., et al. (2019). Early lineage specification defines alveolar epithelial ontogeny in the murine lung. Proc. Natl. Acad. Sci. USA *116*, 4362–4371. https://doi.org/10.1073/pnas.1813952116.

Gan, K.J., and Südhof, T.C. (2020). SPARCL1 Promotes Excitatory But Not Inhibitory Synapse Formation and Function Independent of Neurexins and Neuroligins. J. Neurosci. *40*, 8088–8102. https://doi.org/10.1523/JNEUROSCI.0454-20.2020.

Gillich, A., Zhang, F., Farmer, C.G., Travaglini, K.J., Tan, S.Y., Gu, M., Zhou, B., Feinstein, J.A., Krasnow, M.A., and Metzger, R.J. (2020). Capillary cell-type specialization in the alveolus. Nature *586*, 785–789. https://doi.org/10.1038/s41586-020-2822-7.

Girard, J.-P., and Springer, T.A. (1996). Modulation of Endothelial Cell Adhesion by Hevin, an Acidic Protein Associated with High Endothelial Venules (*). J. Biol. Chem. *271*, 4511–4517. https://doi.org/10.1074/jbc.271.8.4511.

Gkatzis, K., Taghizadeh, S., Huh, D., Stainier, D.Y.R., and Bellusci, S. (2018). Use of three-dimensional organoids and lung-on-a-chip methods to study lung development, regeneration and disease. Eur. Respir. J. *52*, 1800876. https://doi.org/10.1183/13993003.00876-2018.

Gkatzis, K., Panza, P., Peruzzo, S., and Stainier, D.Y. (2021). Differentiation of mouse fetal lung alveolar progenitors in serum-free organotypic cultures. Elife *10*, e65811. https://doi.org/10.7554/eLife.65811.

Gomez-Salinero, J.M., Itkin, T., and Rafii, S. (2021). Developmental angiocrine diversification of endothelial cells for organotypic regeneration. Dev. Cell *56*, 3042–3051. https://doi.org/10.1016/j.devcel.2021.10.020.

Gongidi, V., Ring, C., Moody, M., Brekken, R., Sage, E.H., Rakic, P., and Anton, E.S. (2004). SPARC-like 1 Regulates the Terminal Phase of Radial Glia-Guided Migration in the Cerebral Cortex. Neuron *41*, 57–69. https://doi.org/10.1016/S0896-6273(03)00818-3.

Hassan, D., and Chen, J. (2024). CEBPA restricts alveolar type 2 cell plasticity during development and injury-repair. Nat. Commun. *15*, 4148. https://doi.org/10.1038/s41467-024-48632-3.

Hogan, B.L.M. (2018). Integrating Mechanical Force into Lung Development. Dev. Cell *44*, 273–275. https://doi.org/10.1016/j.devcel.2018.01.015.

Hu, Y., Baud, V., Oga, T., Kim, K.I., Yoshida, K., and Karin, M. (2001). IKKα controls formation of the epidermis independently of NF-κB. Nature *410*, 710–714. https://doi.org/10.1038/35070605.

Ii, M., Matsunaga, N., Hazeki, K., Nakamura, K., Takashima, K., Seya, T., Hazeki, O., Kitazaki, T., and Iizawa, Y. (2006). A Novel Cyclohexene Derivative, Ethyl (6R)-6-[N-(2-Chloro-4-fluorophenyl) sulfamoyl]cyclohex-1-ene-1-carboxylate (TAK-242), Selectively Inhibits Toll-Like Receptor 4-Mediated Cytokine Production through Suppression of Intracellular Signaling. Mol. Pharmacol. *69*, 1288–1295. https://doi.org/10.1124/mol.105.019695.

Kato, K., Diéguez-Hurtado, R., Park, D.Y., Hong, S.P., Kato-Azuma, S., Adams, S., Stehling, M., Trappmann, B., Wrana, J.L., Koh, G.Y., et al. (2018). Pulmonary pericytes regulate lung morphogenesis. Nature Communications *9*, 1–14. https://doi.org/10.1038/s41467-018-04913-2.

Kaufman, C.K., and Fuchs, E. (2000). It's Got You Covered: Nf-κb in the Epidermis. J. Cell Biol. *149*, 999–1004. https://doi.org/10.1083/jcb.149.5.999.

Klingler, A., Regensburger, D., Tenkerian, C., Britzen-Laurent, N., Hartmann, A., Stürzl, M., and Naschberger, E. (2020). Species-organ- And cell-type-dependent expression of SPARCL1 in human and mouse tissues. PLoS One *15*, e0233422. https://doi.org/10.1371/journal.pone.0233422.

Kramer, B.W., Kallapur, S., Newnham, J., and Jobe, A.H. (2009). Prenatal inflammation and lung development. Semin. Fetal Neonatal Med. *14*, 2–7. https://doi.org/10.1016/j.siny.2008.08.011.

Kucukdereli, H., Allen, N.J., Lee, A.T., Feng, A., Ozlu, M.I., Conatser, L.M., Chakraborty, C., Workman, G., Weaver, M., Sage, E.H., et al. (2011). Control of excitatory CNS synaptogenesis by astrocyte-secreted proteins Hevin and SPARC. Proc. Natl. Acad. Sci. USA *108*, E440–E449. https://doi.org/10.1073/pnas.1104977108.

Lazarus, A., Del-Moral, P.M., Ilovich, O., Mishani, E., Warburton, D., and Keshet, E. (2011). A perfusion-independent role of blood vessels in determining branching stereotypy of lung airways. Development *138*, 2359–2368. https://doi.org/10.1242/dev.060723.

Lee, J.H., Bhang, D.H., Beede, A., Huang, T.L., Stripp, B.R., Bloch, K.D., Wagers, A.J., Tseng, Y.H., Ryeom, S., and Kim, C.F. (2014). Lung stem cell differentiation in mice directed by endothelial cells via a BMP4-NFATc1-thrombospondin-1 axis. Cell *156*, 440–455. https://doi.org/10.1016/j.cell.2013.12.039.

Li, J., Wang, Z., Chu, Q., Jiang, K., Li, J., and Tang, N. (2018). The Strength of Mechanical Forces Determines the Differentiation of Alveolar Epithelial Cells. Dev. Cell *44*, 297–312.e5. https://doi.org/10.1016/j.devcel.2018.01.008.

Liu, B., Xiang, L., Ji, J., Liu, W., Chen, Y., Xia, M., Liu, Y., Liu, W., Zhu, P., Jin, Y., et al. (2021). Sparcl1 promotes nonalcoholic steatohepatitis progression in mice through upregulation of CCL2. J. Clin. Investig. *131*, e144801. https://doi.org/10.1172/JCI144801.

Londhe, V.A., Nguyen, H.T., Jeng, J.-M., Li, X., Li, C., Tiozzo, C., Zhu, N., and Minoo, P. (2008). NF-kB induces lung maturation during mouse lung morphogenesis. Dev. Dyn. *237*, 328–338. https://doi.org/10.1002/dvdy.21413.

Londhe, V.A., Maisonet, T.M., Lopez, B., Jeng, J.-M., Xiao, J., Li, C., and Minoo, P. (2011). Conditional deletion of epithelial IKKβ impairs alveolar formation through apoptosis and decreased VEGF expression during early mouse lung morphogenesis. Respir. Res. *12*, 134. https://doi.org/10.1186/1465-9921-12-134.

Naschberger, E., Liebl, A., Schellerer, V.S., Schütz, M., Britzen-Laurent, N., Kölbel, P., Schaal, U., Haep, L., Regensburger, D., Wittmann, T., et al. (2016). Matricellular protein SPARCL1 regulates tumor microenvironment–dependent endothelial cell heterogeneity in colorectal carcinoma. J. Clin. Investig. *126*, 4187–4204. https://doi.org/10.1172/JCI78260.

Negretti, N.M., Plosa, E.J., Benjamin, J.T., Schuler, B.A., Habermann, A.C., Jetter, C.S., Gulleman, P., Bunn, C., Hackett, A.N., Ransom, M., et al. (2021). A single-cell atlas of mouse lung development. Development *148*, dev199512. https://doi.org/10.1242/dev.199512.

Oeckinghaus, A., and Ghosh, S. (2009). The NF-κB Family of Transcription Factors and Its Regulation. Cold Spring Harb. Perspect. Biol. *1*, a000034. https://doi.org/10.1101/cshperspect.a000034.

Pasparakis, M. (2012). Role of NF-κB in epithelial biology: NF-κB in epithelial biology. Immunol. Rev. *246*, 346–358. https://doi.org/10.1111/j.1600-065X.2012.01109.x.

Prince, L.S., Okoh, V.O., Moninger, T.O., and Matalon, S. (2004). Lipopolysaccharide increases alveolar type II cell number in fetal mouse lungs through Toll-like receptor 4 and NF-κB. Am. J. Physiol. Lung Cell. Mol. Physiol. *287*, L999–L1006. https://doi.org/10.1152/ajplung.00111.2004.

Rafii, S., Cao, Z., Lis, R., Siempos, I.I., Chavez, D., Shido, K., Rabbany, S.Y., and Ding, B.S. (2015). Platelet-derived SDF-1 primes the pulmonary capillary vascular niche to drive lung alveolar regeneration. Nat. Cell Biol. *17*, 123–136. https://doi.org/10.1038/ncb3096.

Ramasamy, S.K., Kusumbe, A.P., and Adams, R.H. (2015). Regulation of tissue morphogenesis by endothelial cell-derived signals.

Trends Cell Biol. *25*, 148–157. https://doi.org/10.1016/j.tcb.2014.11.007.

Regensburger, D., Tenkerian, C., Pürzer, V., Schmid, B., Wohlfahrt, T., Stolzer, I., López-Posadas, R., Günther, C., Waldner, M.J., Becker, C., et al. (2021). Matricellular Protein SPARCL1 Regulates Blood Vessel Integrity and Antagonizes Inflammatory Bowel Disease. Inflamm. Bowel Dis. *27*, 1491–1502. https://doi.org/10.1093/ibd/izaa346.

Tabula Muris Consortium; Overall coordination; Logistical coordination; Organ collection and processing; Library preparation and sequencing; Computational data analysis; Cell type annotation; Writing group; Supplemental text writing group; Principal investigators (2018). Single-cell transcriptomics of 20 mouse organs creates a Tabula Muris. Nature *562*, 367–372. https://doi.org/10.1038/s41586-018-0590-4.

Schmidt, A.F., Kannan, P.S., Bridges, J., Presicce, P., Jackson, C.M., Miller, L.A., Kallapur, S.G., Chougnet, C.A., and Jobe, A.H. (2020). Prenatal inflammation enhances antenatal corticosteroid–induced fetal lung maturation. JCI Insight *5*, e139452. https://doi.org/10.1172/jci.insight.139452.

Schmidt-Ullrich, R., Aebischer, T., Hülsken, J., Birchmeier, W., Klemm, U., and Scheidereit, C. (2001). Requirement of NF-κB/Rel for the development of hair follicles and other epidermal appendices. Development *128*, 3843–3853. https://doi.org/10.1242/dev.128.19.3843.

Seitz, C.S., Lin, Q., Deng, H., and Khavari, P.A. (1998). Alterations in NF-κB function in transgenic epithelial tissue demonstrate a growth inhibitory role for NF-κB. Proc. Natl. Acad. Sci. USA *95*, 2307–2312. https://doi.org/10.1073/pnas.95.5.2307.

Seitz, C.S., Deng, H., Hinata, K., Lin, Q., and Khavari, P.A. (2000). Nuclear Factor κB Subunits Induce Epithelial Cell Growth Arrest1. Cancer Res. *60*, 4085–4092.

Shin, H.-M., Kim, M.-H., Kim, B.H., Jung, S.-H., Kim, Y.S., Park, H.J., Hong, J.T., Min, K.R., and Kim, Y. (2004). Inhibitory action of novel aromatic diamine compound on lipopolysaccharide-induced nuclear translocation of NF-κB without affecting IκB degradation. FEBS Lett. *571*, 50–54. https://doi.org/10.1016/j.febslet.2004.06.056.

Singh, S.K., Stogsdill, J.A., Pulimood, N.S., Dingsdale, H., Kim, Y.H., Pilaz, L.-J., Kim, I.H., Manhaes, A.C., Rodrigues, W.S., Pamukcu, A., et al. (2016). Astrocytes Assemble Thalamocortical Synapses by Bridging NRX1α and NL1 via Hevin. Cell *164*, 183–196. https://doi.org/10.1016/j.cell.2015.11.034.

Strunz, M., Simon, L.M., Ansari, M., Kathiriya, J.J., Angelidis, I., Mayr, C.H., Tsidiridis, G., Lange, M., Mattner, L.F., Yee, M., et al. (2020). Alveolar regeneration through a Krt8+ transitional stem cell state that persists in human lung fibrosis. Nat. Commun. *11*, 3559. https://doi.org/10.1038/s41467-020-17358-3.

Subramanian, A., Tamayo, P., Mootha, V.K., Mukherjee, S., Ebert, B.L., Gillette, M.A., Paulovich, A., Pomeroy, S.L., Golub, T.R., Lander, E.S., and Mesirov, J.P. (2005). Gene set enrichment analysis: A knowledge-based approach for interpreting genome-wide expression profiles. Proc. Natl. Acad. Sci. USA *102*, 15545–15550. https://doi.org/10.1073/pnas.0506580102.

Sullivan, M.M., and Sage, E.H. (2004). Hevin/SC1, a matricellular glycoprotein and potential tumor-suppressor of the SPARC/BM-40/Osteonectin family. Int. J. Biochem. Cell Biol. *36*, 991–996. https://doi.org/10.1016/j.biocel.2004.01.017.

Tabula Sapiens Consortium*, Jones, R.C., Karkanias, J., Krasnow, M.A., Pisco, A.O., Quake, S.R., Salzman, J., Yosef, N., Bulthaup, B., Brown, P., et al. (2022). The Tabula Sapiens: A multiple-organ, single-cell transcriptomic atlas of humans. Science *376*, eabl4896. https://doi.org/10.1126/science.abl4896.

Treutlein, B., Brownfield, D.G., Wu, A.R., Neff, N.F., Mantalas, G.L., Espinoza, F.H., Desai, T.J., Krasnow, M.A., and Quake, S.R. (2014). Reconstructing lineage hierarchies of the distal lung epithelium using single-cell RNA-seq. Nature *509*, 371–375. https://doi.org/10.1038/nature13173.

Vogel, S.N., Wax, J.S., Perera, P.Y., Padlan, C., Potter, M., and Mock, B.A. (1994). Construction of a BALB/c congenic mouse, C.C3H-Lpsd, that expresses the Lpsd allele: analysis of chromosome 4 markers surrounding the Lps gene. Infect. Immun. *62*, 4454–4459. https://doi.org/10.1128/iai.62.10.4454-4459.1994.

Weibel, E.R. (1984). The Pathway for Oxygen: Structure and Function in the Mammalian Respiratory System (Harvard Univ. Press).

Whitsett, J.A., and Alenghat, T. (2015). Respiratory epithelial cells orchestrate pulmonary innate immunity. Nat. Immunol. *16*, 27–35. https://doi.org/10.1038/ni.3045.

Whitsett, J.A., Wert, S.E., and Weaver, T.E. (2015). Diseases of pulmonary surfactant homeostasis. Annu. Rev. Pathol. *10*, 371–393. https://doi.org/10.1146/annurev-pathol-012513-104644.

Yamamoto, Y., Shiraishi, I., Dai, P., Hamaoka, K., and Takamatsu, T. (2007). Regulation of embryonic lung vascular development by vascular endothelial growth factor receptors, Flk-1 and Flt-1. Anat. Rec. *290*, 958–973. https://doi.org/10.1002/ar.20564.

Zanini, F., Che, X., Knutsen, C., Liu, M., Suresh, N.E., Domingo-Gonzalez, R., Dou, S.H., Zhang, D., Pryhuber, G.S., Jones, R.C., et al. (2023). Developmental diversity and unique sensitivity to injury of lung endothelial subtypes during postnatal growth. iScience *26*, 106097. https://doi.org/10.1016/j.isci.2023.106097.

Zhao, G., Gentile, M.E., Xue, L., Cosgriff, C.V., Weiner, A.I., Adams-Tzivelekidis, S., Wong, J., Li, X., Kass-Gergi, S., Holcomb, N.P., et al. (2024). Vascular endothelial-derived SPARCL1 exacerbates viral pneumonia through pro-inflammatory macrophage activation. Nat. Commun. *15*, 4235. https://doi.org/10.1038/s41467-024-48589-3.

Stem Cell Reports, Volume *20*

Supplemental Information

The lung microvasculature promotes alveolar type 2 cell differentiation via secreted SPARCL1

Paolo Panza, Hyun-Taek Kim, Till Lautenschläger, Janett Piesker, Stefan Günther, Yousef Alayoubi, Ondine Cleaver, Mario Looso, and Didier Y.R. Stainier

SUPPLEMENTAL FIGURES

Figure S1

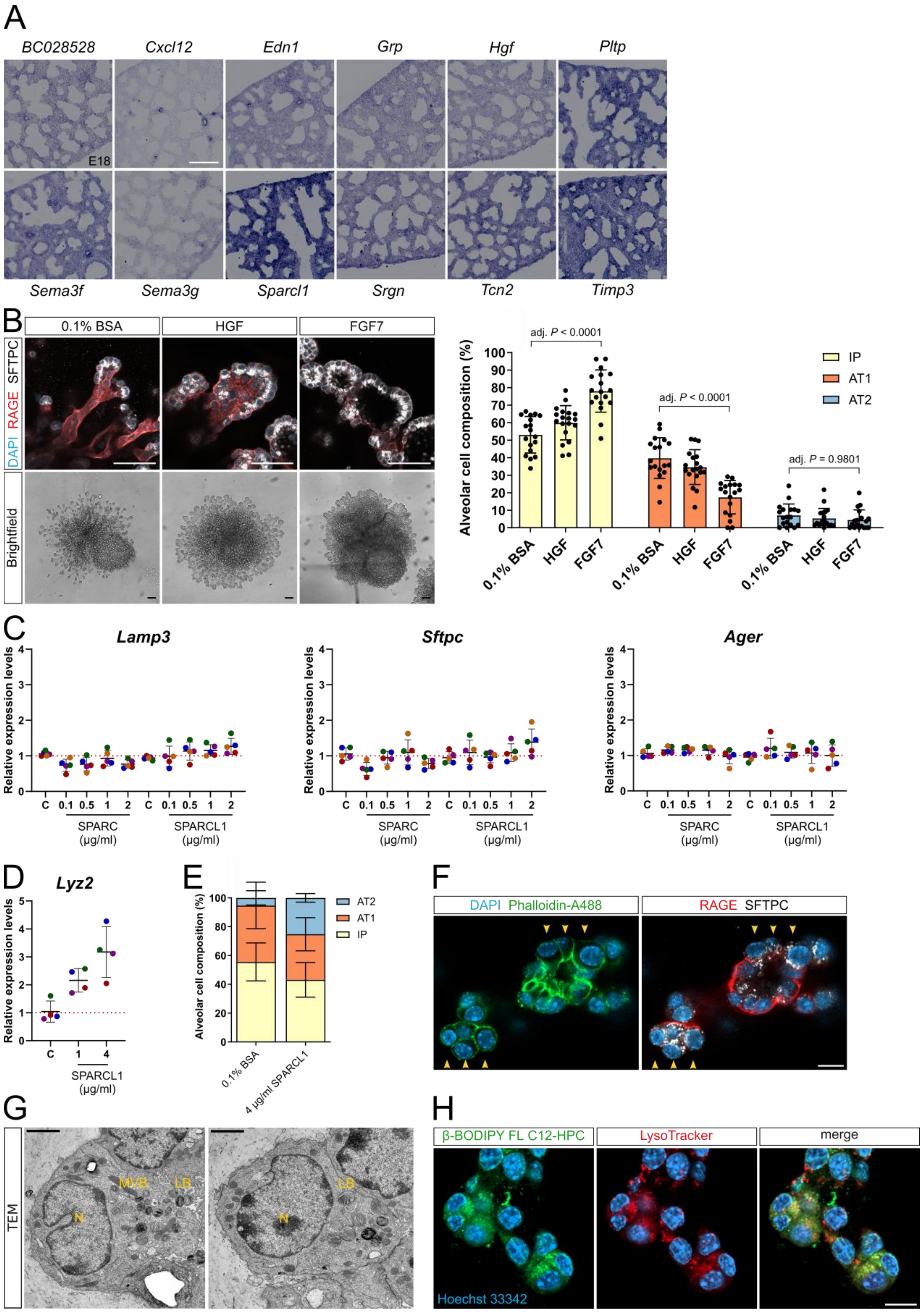

Figure S2

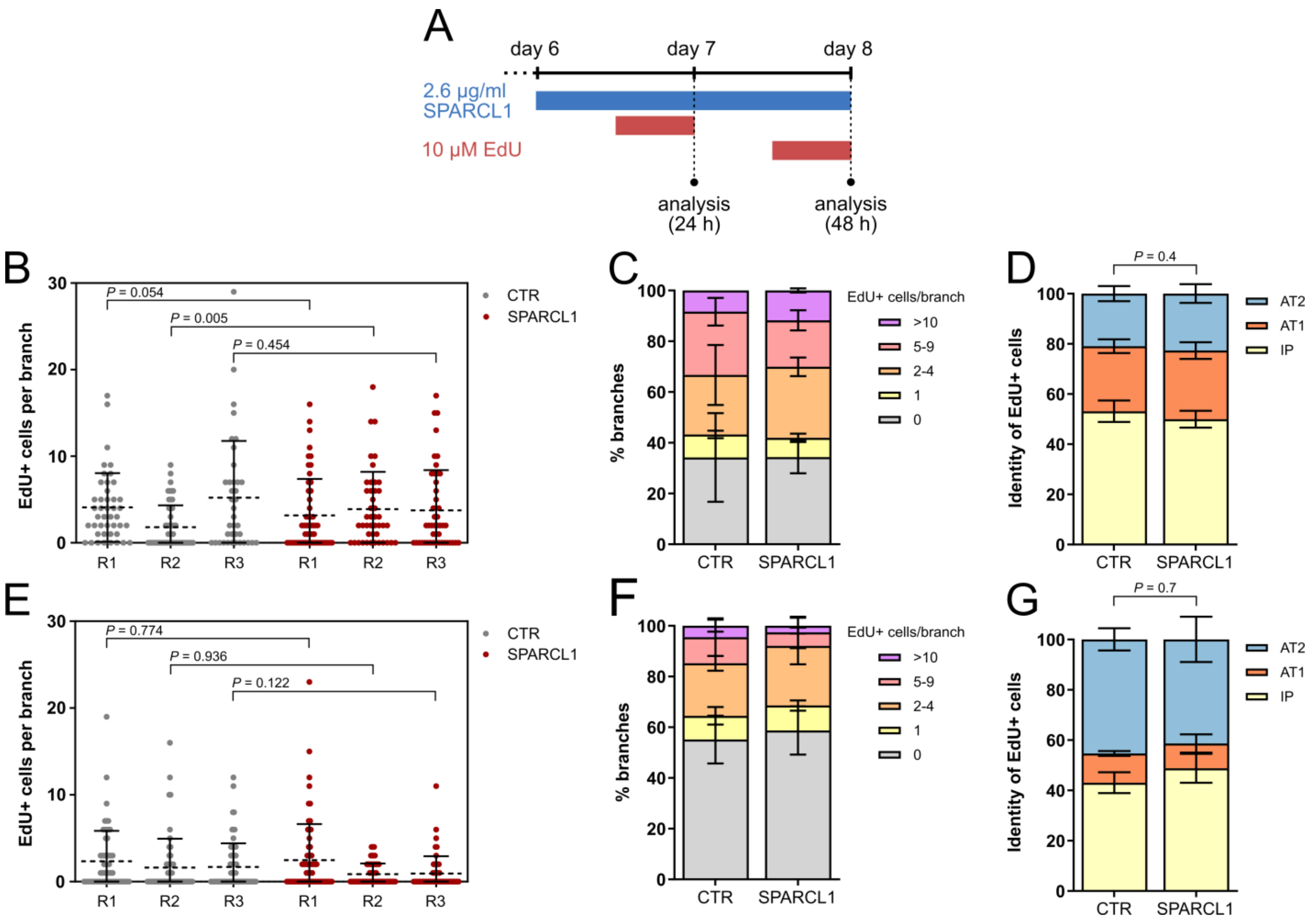
A
day 6
day 7
day 8
2.6 µg/ml SPARCL1
10 µM EdU
analysis (24 h)
analysis (48 h)
B
EdU+ cells per branch
P = 0.054
P = 0.005
P = 0.454
CTR
SPARCL1
R1
R2
R3
R1
R2
R3
C
% branches
EdU+ cells/branch
>10
5-9
2-4
1
0
CTR
SPARCL1
D
P = 0.4
Identity of EdU+ cells
AT2
AT1
IP
CTR
SPARCL1
E
EdU+ cells per branch
P = 0.774
P = 0.936
P = 0.122
CTR
SPARCL1
R1
R2
R3
R1
R2
R3
F
% branches
EdU+ cells/branch
>10
5-9
2-4
1
0
CTR
SPARCL1
G
P = 0.7
Identity of EdU+ cells
AT2
AT1
IP
CTR
SPARCL1

Figure S3

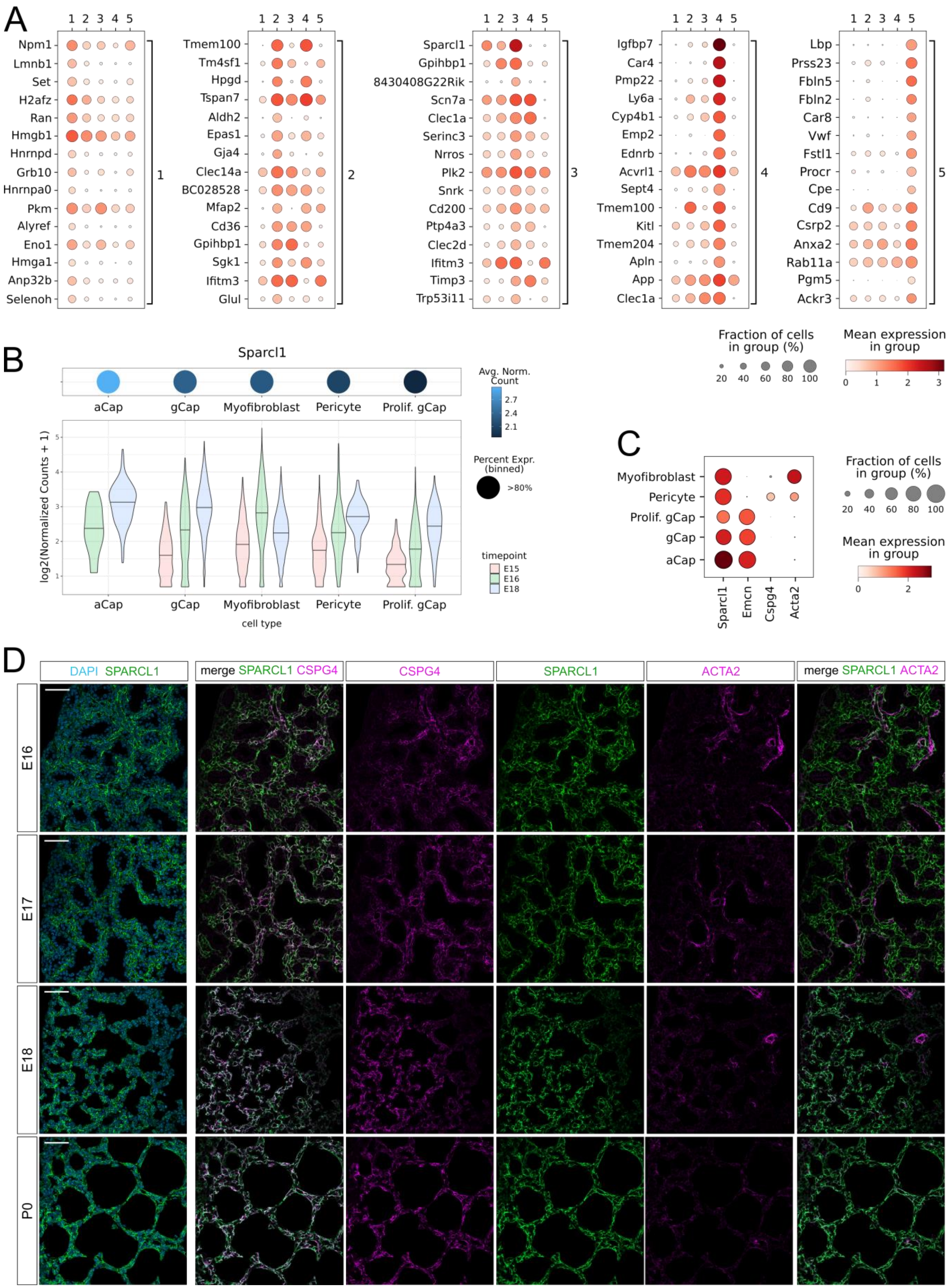
A
1 2 3 4 5
Npm1
Lmnb1
Set
H2afz
Ran
Hmgb1
Hnrnpd
Grb10
Hnrnpa0
Pkm
Alyref
Eno1
Hmga1
Anp32b
Selenoh
1
Tmem100
Tm4sf1
Hpgd
Tspan7
Aldh2
Epas1
Gja4
Clec14a
BC028528
Mfap2
Cd36
Gpihbp1
Sgk1
Ifitm3
Glul
2
Sparcl1
Gpihbp1
8430408G22Rik
Scn7a
Clec1a
Serinc3
Nrros
Plk2
Snrk
Cd200
Ptp4a3
Clec2d
Ifitm3
Timp3
Trp53i11
3
Igfbp7
Car4
Pmp22
Ly6a
Cyp4b1
Emp2
Ednrb
Acvrl1
Sept4
Tmem100
Kitl
Tmem204
Apln
App
Clec1a
4
Lbp
Prss23
Fbln5
Fbln2
Car8
Vwf
Fstl1
Procr
Cpe
Cd9
Csrp2
Anxa2
Rab11a
Pgm5
Ackr3
5
Fraction of cells in group (%)
20 40 60 80 100
Mean expression in group
0 1 2 3
B
Sparcl1
aCap gCap Myofibroblast Pericyte Prolif. gCap
Avg. Norm. Count
2.7
2.4
2.1
log2(Normalized Counts + 1)
Percent Expr. (binned)
>80%
timepoint
E15
E16
E18
cell type
C
Myofibroblast
Pericyte
Prolif. gCap
gCap
aCap
Sparcl1 Emcn Cspg4 Acta2
Fraction of cells in group (%)
20 40 60 80 100
Mean expression in group
0 2
D
DAPI SPARCL1
merge SPARCL1 CSPG4
CSPG4
SPARCL1
ACTA2
merge SPARCL1 ACTA2
E16
E17
E18
P0

Figure S4

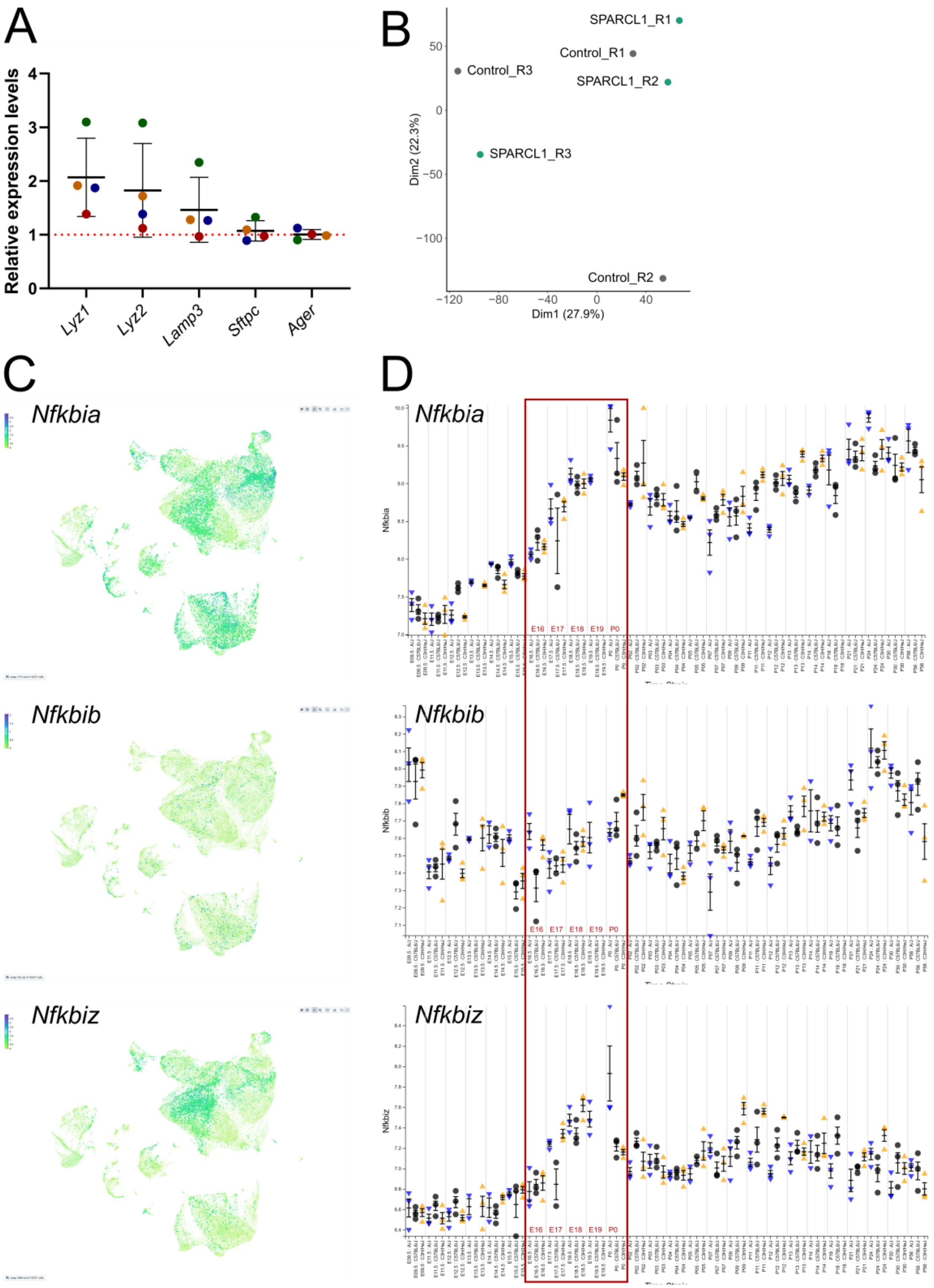
A
Relative expression levels
0
1
2
3
4
Lyz1
Lyz2
Lamp3
Sftpc
Ager
B
SPARCL1_R1
Control_R1
SPARCL1_R2
Control_R3
SPARCL1_R3
Control_R2
50
0
−50
−100
Dim2 (22.3%)
−120
−80
−40
0
40
Dim1 (27.9%)
C
Nfkbia
Nfkbib
Nfkbiz
D
Nfkbia
Nfkbib
Nfkbiz
E16
E17
E18
E19
P0

Figure S5

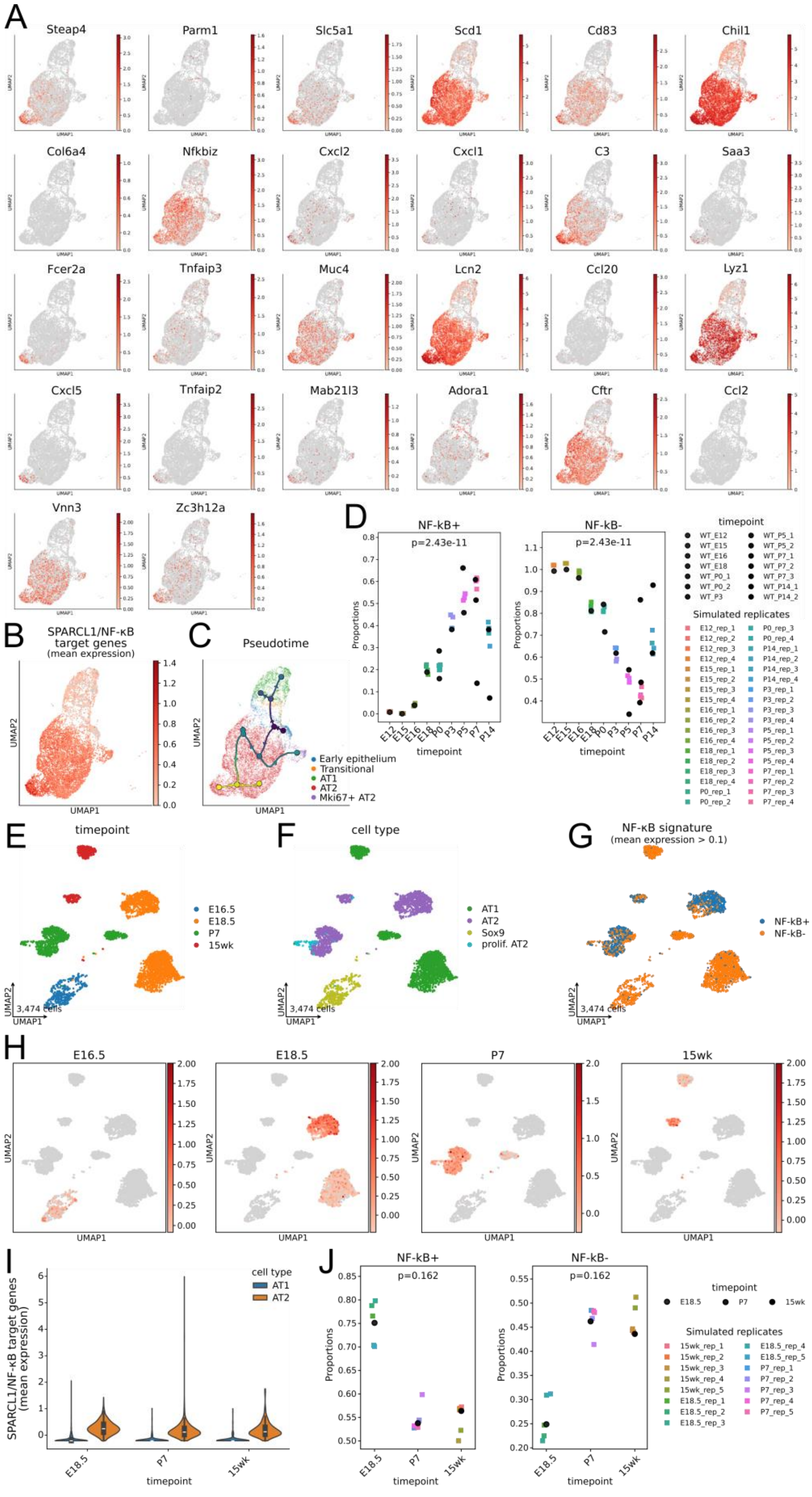

A
Steap4
Parm1
Slc5a1
Scd1
Cd83
Chil1
Col6a4
Nfkbiz
Cxcl2
Cxcl1
C3
Saa3
Fcer2a
Tnfaip3
Muc4
Lcn2
Ccl20
Lyz1
Cxcl5
Tnfaip2
Mab21l3
Adora1
Cftr
Ccl2
Vnn3
Zc3h12a
B
SPARCL1/NF-κB target genes (mean expression)
C
Pseudotime
Early epithelium
Transitional
AT1
AT2
Mki67+ AT2
D
NF-kB+
NF-kB-
p=2.43e-11
Proportions
timepoint
Simulated replicates
E
timepoint
E16.5
E18.5
P7
15wk
3,474 cells
F
cell type
AT1
AT2
Sox9
prolif. AT2
G
NF-κB signature (mean expression > 0.1)
NF-kB+
NF-kB-
H
E16.5
E18.5
P7
15wk
UMAP1
UMAP2
I
SPARCL1/NF-κB target genes (mean expression)
cell type
AT1
AT2
timepoint
J
NF-kB+
NF-kB-
p=0.162
Proportions
timepoint
Simulated replicates

SUPPLEMENTAL FIGURE LEGENDS

Figure S1. (A) *In situ* hybridization for genes encoding candidate EC secreted proteins on E18 lung cryosections. *Cxcl12* and *Edn1* are expressed in proximal blood vessels; *Sparcl1* and *Pltp* are expressed in a reticular pattern within the distal lung interstitium, consistent with their expression in blood capillary and/or mural cells. *Timp3* is highly expressed in AT1 cells. Scale bar: 100 µm. **(B)** Growth factor treated organoids display a morphological phenotype and an increased proportion of IP cells. Left, top row: whole-mount organoid immunostaining for SFTPC (IP and AT2 cells) and RAGE (IP and AT1 cells). Organoids treated with HGF (1 µg/mL) and FGF7 (10 ng/mL) contain more IP cells ($SFTPC^{+}/RAGE^{+}$) and fewer AT1 cells ($SFTPC^{-}/RAGE^{+}$). Left, bottom row: brightfield images of representative organoids treated with 0.1% BSA, HGF, and FGF7. Right: quantification of cell identities in growth factor-treated organoids. FGF7 treated organoids display an increased proportion of IP cells at the expense of AT1 cells (adj. $p < 0.0001$, $n = 3$ dams, at least 6 organoids per condition). Scale bars: 50 µm (top row); 100 µm (bottom row). Data are presented as mean ± SD. *p* values are from one-way ANOVA, Tukey's multiple comparison testing. **(C)** Minor dose dependent increase in *Lamp3* and *Sftpc* mRNA levels in organoids treated with SPARCL1, but not with SPARC. *Ager* mRNA levels did not change upon treatment with recombinant SPARCL1 or SPARC ($n = 5$ dams, at least 12 organoids per condition). Data are presented as mean ± SD. **(D)** High dose SPARCL1 (4 µg/mL) further increases *Lyz2* mRNA levels in organoids, compared with the lower dose (1 µg/mL) used for functional screening ($n = 4$ dams, at least 12 organoids per condition). Data are presented as mean ± SD. **(E)** Increased proportion of AT2 cells in organoids treated with SPARCL1 at 4 µg/mL ($n = 3$ dams, at least 4 organoids per condition). AT2: alveolar type 2 cell ($SFTPC^{+}/RAGE^{-}$); AT1: alveolar type 1 cell ($SFTPC^{-}/RAGE^{+}$); IP: intermediate progenitor cell ($SFTPC^{+}/RAGE^{+}$). Data are presented as mean ± SD. **(F)** High magnification detail of AT2 cells in SPARCL1-treated organoids. AT2 cells contain SFTPC-positive organelles (white) and are devoid of RAGE signal (red) on their basal membrane. Phalloidin staining of cortical actin (green) marks the cell boundaries. Scale bar: 10 µm. **(G)** Transmission electron microscopy (TEM) images of representative epithelial cells in distal regions of organoid outgrowths. Panels show examples of AT2 cells, as identified by the presence of characteristic lamellar bodies (LBs) and multivesicular bodies (MVBs). N: nucleus. Scale bars: 2 µm. **(H)** Distal tip cells in organoids take up labeled phosphatidylcholine into acidic organelles; live fluorescence in whole-mount organoids. Hoechst 33342 (DNA, blue); β-BODIPY FL C12-HPC (fluorescent phosphatidylcholine, green); LysoTracker Deep Red (lamellar bodies/lysosomes, red). Distal tip cells consistent with AT2 cell morphology and characteristics display co-localizing green and red signals. Scale bar: 10 µm.

Figure S2. (A) Workflow schematic for the analysis of cell proliferation in organoids. Control and SPARCL1-treated (2.6 µg/mL) organoids ($n = 3$ dams, at least 12 organoids per condition) were terminally labeled by 10 µM EdU for 12 h and samples were collected after 24 and 48 h. **(B)** Distribution of EdU^{+} cell counts per branch in control (grey) and SPARCL1-treated (red) organoids (day 7). One replicate (R2) out of three displayed a significant increase in EdU^{+} cell number in SPARCL1-treated organoids ($p = 0.005$). **(C)** Relative distribution of EdU^{+} cell counts per organoid branch (day 7). **(D)** Proportion (percentage) of EdU^{+} cell identities observed on day 7. The proportion of EdU^{+} AT2 cells did not change significantly in SPARCL1-treated organoids. **(E)** Distribution of EdU^{+} cell counts per branch in control (grey) and SPARCL1-treated (red) organoids (day 8). The EdU^{+} cell number did not change significantly in SPARCL1-treated organoids. **(F)** Relative distribution of EdU^{+} cell counts per organoid branch (day 8). **(G)** Proportion (percentage) of

EdU+ cell identities observed on day 8. The proportion of EdU+ AT2 cells did not change significantly in SPARCL1-treated organoids. All data are presented as mean ± SD. All *p* values are from Mann-Whitney U test.

Figure S3. (A) Marker gene expression analysis of EC clusters identified (E12-P3). Cells in clusters 2 and 3 are identified as general capillary (gCap) cells because of the exclusive expression of *Gpihbp1* (Gillich et al., 2020). *Tmem100* expression identifies clusters 2 and 4 as predominantly postnatal (Liu et al., 2022). *Car4*, *Kitl* and *Apln* expression marks cluster 4 cells, identifying them as alveolar capillary (aCap) cells (Gillich et al., 2020). **(B)** The mRNA expression levels of *Sparcl1* are comparable between lung ECs and pericytes at stages E15 to E18. Normalized *Sparcl1* read counts from single lung cells profiled at E15, E16, and E18. Data and cell type annotation from Negretti et al., 2021. **(C)** Expression of *Emcn*, *Cspg4*, and *Acta2* distinguish ECs, pericytes, and myofibroblasts in the E15-E18 lung. Data and cell type annotation from Negretti et al., 2021. **(D)** SPARCL1 expression prevails in distal lung pericytes compared with myofibroblasts between E16-P0. Immunostaining of E16-P0 (top to bottom rows) lung cryosections, single optical sections. Left: overview of SPARCL1 expression (green) in distal lung regions. Other panels (left to right): merge between SPARCL1 (green) and CSPG4 (magenta, pericytes); single channel CSPG expression (magenta); single channel SPARCL1 expression (green); single channel ACTA2 expression (magenta, myofibroblasts); merge between SPARCL1 (green) and ACTA2 (magenta, myofibroblasts). Scale bars: 50 µm.

Figure S4. (A) The recombinant SPARCL1 protein used for the RNA-seq experiments is biologically active. Recombinant SPARCL1 induced *Lyz1*, *Lyz2* and *Lamp3* upregulation (48 h treatments) in organoids from the same primary tissue source as those profiled by RNA-seq (24 h treatments). $n = 4$ dams, at least 16 organoids per condition. Replicates represented in green, orange and blue were selected for RNA-seq. Data are presented as mean ± SD. **(B)** Principal component analysis (PCA) plot for sequenced RNA samples shows intermixing between conditions (control and SPARCL1-treated). **(C)** Broad expression and perinatal increase in whole-lung expression levels for *Nfkbia* and *Nfkbiz*. Lower and stable expression level for *Nfkbib* in the developing lung. Gene expression levels (green-to-blue color scale) in a single cell atlas comprising mouse lung development (E12-P14, Negretti et al., 2021). **(D)** Gene expression level dynamics in whole lungs, data for 3 inbred mouse strains (Beauchemin et al., 2016). Blue: A/J; black: C57BL6/J, yellow: C3H/HeJ.

Figure S5. (A) Split-view of expression levels in single epithelial cells for genes identified as SPARCL1-responsive. Plots include single alveolar epithelial cell transcriptomes from E12 to P7. Data from Negretti et al., 2021. **(B)** Mean expression level for all identified SPARCL1/NF-κB target genes in alveolar epithelial cells profiled from E12 to P7. A subset of epithelial cells express SPARCL1-responsive genes starting at E18 and through postnatal stages P0-P7. **(C)** Similarity-based pseudotime analysis correlates increasing SPARCL1/NF-κB target gene expression with maturing AT2 cells. Lineage progression: purple to yellow. **(D)** The proportion of epithelial cells expressing SPARCL1/NF-κB target genes (NF-kB+) increases significantly from E18 in developing lungs. Conversely, epithelial cells characterized as NF-κB negative (NF-kB-) decrease in prevalence from E18. *p* values empirical Bayes moderated ANOVA test.
(E) Leiden clustering of single lung alveolar epithelial cell transcriptomes from 4 stages (E16.5, E18.5, P7, 15 weeks). Data from Hassan and Chen, 2024. **(F-G)** Cells categorized to express the SPARCL1/NF-κB target genes (blue, NF-kB+) are found in higher proportion within clusters annotated as AT2 cells (purple) at all analyzed stages. **(H)** Expression levels in single epithelial cells for genes identified as SPARCL1-responsive. Plots display single alveolar

epithelial cell transcriptomes from each stage analyzed. **(I)** Stage-resolved comparison of the mean expression level for SPARCL1/NF-κB target genes between AT1 and AT2 cells, showing consistently higher expression in AT2 cells independent from the time point, at comparable levels across the stages analyzed. **(J)** In mouse lungs, the proportion of AT2 cells expressing SPARCL1-responsive NF-κB genes (NF-kB+) decreases from E18.5 to 15 weeks, albeit not significantly. *p* values are from empirical Bayes moderated ANOVA test.

.

Table S1

Ensembl gene ID	Ensembl gene	Condition mean E18.5 lung	E18.5 lung vs. E15.5 lung log2 fold change	Signal peptide (found or predicted)	TM Keyword found (UniProt)	GPI anchor	Matrisome cell source	Matrisome type	Matrisome level	Matrisome secreted	Kidney Pancreas Log2FC E18.5 > E15.5	Exp. Level E18.5 > E15.5 (15)	scRNA-seq expression (LGEA, E18)	scRNA-seq marker gene (LGEA, E18)
ENSMUSG00000029375	Cxcl15	6649	8.55	TRUE	FALSE		AT2	Secreted Factors	Matrisome-associated	TRUE	FALSE	Y	AT2	Y
ENSMUSG00000026874	Hc	578	6.82	TRUE	FALSE					FALSE	FALSE	Y	AT2	Y
ENSMUSG00000031722	Hp	1133	6.81	TRUE	FALSE					FALSE	FALSE	Y	Myeloid; Epi	
ENSMUSG00000063011	Msln	1298	6.76	TRUE	FALSE	Y				FALSE	FALSE	Y	AT1	Y
ENSMUSG00000026822	Lcn2	268	6.04	TRUE	FALSE					FALSE	FALSE	Y	AT2	Y
ENSMUSG00000069516	Lyz2	5166	5.91	TRUE	FALSE					FALSE	TRUE	Y	Myeloid; AT2	
ENSMUSG00000000805	Car4	655	5.38	TRUE	FALSE	Y				FALSE	FALSE	Y	EC; Myeloid	
ENSMUSG00000002204	Napsa	1264	5.36	TRUE	FALSE					FALSE	FALSE	Y	Epithelium	Y
ENSMUSG00000033880	Lgals3bp	673	4.78	TRUE	FALSE					FALSE	TRUE	Y	Myeloid, EC	
ENSMUSG00000060962	Dmkn	61	3.71	TRUE	FALSE					FALSE	FALSE	Y low	Epithelium	
ENSMUSG00000030093	Wnt7a	27	3.32	TRUE	FALSE		Other	Secreted Factors	Matrisome-associated	TRUE	FALSE		AT1	Y
ENSMUSG00000007279	Scube2	56	2.54	TRUE	FALSE		EC	Secreted Factors	Matrisome-associated	TRUE	TRUE	Y	Matrix FB	Y
ENSMUSG00000028864	Hgf	96	2.46	TRUE	FALSE		EC	Secreted Factors	Matrisome-associated	TRUE	FALSE	Y low	Myeloid; EC	
ENSMUSG00000030790	Adm	136	2.43	TRUE	FALSE					FALSE	FALSE	Y	EC; FB	
ENSMUSG00000021367	Edn1	2251	2.32	TRUE	FALSE		EC			FALSE	FALSE	Y	EC	Y
ENSMUSG00000020044	Timp3	16922	2.27	TRUE	FALSE			ECM Regulators	Matrisome-associated	FALSE	FALSE	Y	AT1; EC; FB	
ENSMUSG00000017754	Pltp	3914	2.17	TRUE	FALSE					FALSE	FALSE	Y low	EC; FB	
ENSMUSG00000020432	Tcn2	7172	2.17	TRUE	FALSE					FALSE	FALSE	Y	Ubi	
ENSMUSG00000038264	Sema7a	1750	2.02	TRUE	FALSE	Y		ECM-affiliated Proteins	Matrisome-associated	FALSE	TRUE	Y	EC	Y
ENSMUSG00000039899	Fgl2	313	2.02	TRUE	FALSE			ECM Glycoproteins	Core matrisome	FALSE	FALSE	Y	EC; Myeloid	
ENSMUSG00000034684	Sema3f	3472	1.98	TRUE	FALSE			ECM-affiliated Proteins	Matrisome-associated	FALSE	FALSE	Y	EC	
ENSMUSG00000029309	Sparcl1	54062	1.73	TRUE	FALSE			ECM Glycoproteins	Core matrisome	FALSE	FALSE	Y	EC; PC; FB	
ENSMUSG00000024517	Grp	273	1.50	TRUE	FALSE					FALSE	FALSE	Y low	EC; PC	
ENSMUSG00000026365	Cfh	96	1.46	TRUE	FALSE					FALSE	FALSE	Y	Myeloid, FB	
ENSMUSG00000060802	B2m	7019	1.40	TRUE	FALSE					FALSE	FALSE	Y	Ubi	
ENSMUSG00000021242	Npc2	5784	1.35	TRUE	FALSE					FALSE	FALSE	Y	AT2	Y
ENSMUSG00000021186	Fbln5	3173	1.29	TRUE	FALSE			ECM Glycoproteins	Core matrisome	FALSE	FALSE	Y	AT1; FB; EC	
ENSMUSG00000038543	BC028528	1381	1.28	TRUE	FALSE					FALSE	FALSE	Y low	EC; PC	
ENSMUSG00000046618	Olfml2a	378	1.27	TRUE	FALSE					FALSE	FALSE		EC; PC	
ENSMUSG00000020077	Srgn	3599	1.24	TRUE	FALSE			Proteoglycans	Core matrisome	FALSE	FALSE	Y	EC; Myeloid	
ENSMUSG00000020902	Ntn1	320	1.17	TRUE	FALSE			ECM Glycoproteins	Core matrisome	FALSE	FALSE			
ENSMUSG00000061353	Cxcl12	3047	1.09	TRUE	FALSE		EC	Secreted Factors	Matrisome-associated	TRUE	FALSE		EC; PC	
ENSMUSG00000021904	Sema3g	609	1.08	TRUE	FALSE			ECM-affiliated Proteins	Matrisome-associated	FALSE	FALSE	Y	EC	
ENSMUSG00000031503	Col4a2	16698	1.05	TRUE	FALSE			Collagens	Core matrisome	FALSE	FALSE	Y	EC; PC; FB	
ENSMUSG00000064080	Fbln2	752	1.03	TRUE	FALSE			ECM Glycoproteins	Core matrisome	FALSE	FALSE		EC; PC; FB	

Table S2

Ensembl gene id	Ensembl gene	baseMean	baseMeanA CONTROL	baseMeanB TREATED	log2FoldChange TREATED/CONTROL	pvalue	padj
ENSMUSG00000040026	Saa3	97	4	190	5.16	0.00	0.00
ENSMUSG00000029371	Cxcl5	116	15	217	3.74	0.00	0.00
ENSMUSG00000026822	Lcn2	211	48	374	2.94	0.00	0.00
ENSMUSG00000024164	C3	329	110	548	2.31	0.00	0.00
ENSMUSG00000026166	Ccl20	30	11	48	2.05	0.00	0.00
ENSMUSG00000044313	Mab21l3	82	33	132	1.96	0.00	0.00
ENSMUSG00000058427	Cxcl2	38	17	60	1.77	0.00	0.00
ENSMUSG00000005540	Fcer2a	81	39	124	1.65	0.00	0.00
ENSMUSG00000029380	Cxcl1	78	38	117	1.60	0.00	0.00
ENSMUSG00000012428	Steap4	301	156	446	1.51	0.00	0.04
ENSMUSG00000035385	Ccl2	81	42	119	1.49	0.00	0.00
ENSMUSG00000064246	Chil1	683	359	1007	1.48	0.00	0.00
ENSMUSG00000021281	Tnfaip2	66	38	94	1.29	0.00	0.01
ENSMUSG00000015396	Cd83	164	101	228	1.17	0.00	0.00
ENSMUSG00000020010	Vnn3	273	171	374	1.12	0.00	0.00
ENSMUSG00000042429	Adora1	375	299	451	0.59	0.00	0.00
ENSMUSG00000019850	Tnfaip3	594	490	697	0.51	0.00	0.00
ENSMUSG00000041301	Cftr	653	546	760	0.48	0.00	0.02
ENSMUSG00000037071	Scd1	1332	1128	1535	0.44	0.00	0.00
ENSMUSG00000011034	Slc5a1	53	33	74	1.16	0.00	0.06
ENSMUSG00000042677	Zc3h12a	197	156	238	0.60	0.00	0.07
ENSMUSG00000069515	Lyz1	101	70	132	0.91	0.00	0.07
ENSMUSG00000032572	Col6a4	338	262	415	0.66	0.00	0.10
ENSMUSG00000034981	Parm1	230	187	273	0.54	0.00	0.10
ENSMUSG00000118672	Muc4	414	345	482	0.48	0.00	0.10
ENSMUSG00000027533	Fabp5	182	141	223	0.65	0.00	0.13
ENSMUSG00000048732	Klhl11	135	100	170	0.76	0.00	0.18

Table S3

Gene	Forward Sequence (5'-3')	Reverse Sequence (5'-3')
Actb	CTCTGGCTCCTAGCACCATGAAGA	GTAAAACGCAGCTCAGTAACAGT
Maea	CACTGAACAAACGCTTCCGAG	GGCAACTACTCAAGGTCTTCTC
Ager	ACGGGACTCTTTACACTGCG	CAACCAACAGCTGAATGCCC
Sftpc	AGCAGACACCATCGCTACCT	GCAGTAGGTTCCTGGAGCTG
Lamp3	TCCAAAAGCCAGAGGCTATCT	ACTGGGGTTACTGTTTTCATTGT
Lyz1	GAGACCGAAGCACCGACTATG	CGGTTTTGACATTGTGTTCGC
Lyz2 (pair 1)	GATGGCAAAACCCCAAGAGC	CAGACTCCGCAGTTCCGAAT
Lyz2 (pair 2)	ATGGAATGGCTGGCTACTATGG	ACCAGTATCGGCTATTGATCTGA
Nfkbia	TGAAGGACGAGGAGTACGAGC	TTCGTGGATGATTGCCAAGTG
Nfkbib	GCGGATGCCGATGAATGGT	TGACGTAGCCAAAGACTAAGGG
Nfkbiz	GCTCCGACTCCTCCGATTTC	GAGTTCTTCACGCGAACACC

Table S1. Candidate endothelial cell (EC) secreted factors. Genes were prioritized based on their higher expression at E18.5 compared with E15.5 in lung ECs (log2FC > 1). Only genes encoding secreted proteins (with predicted signal peptide, without transmembrane domain (TM)) were included in the analysis. Source transcriptomic data from (Daniel et al., 2018).

Table S2. SPARCL1-responsive gene set. Genes upregulated upon SPARCL1 treatment of mouse lung organoids (24 h) are shown (adj. $p < 0.2$; $p = 0$).

Table S3. RT-qPCR primer sequences used in the study.

Data S1 (separate file). RNA-seq dataset: organoids treated with SPARCL1 vs. 0.1% BSA (24 h).

Data S2 (separate file). Mean Ct values for RT-qPCR experiments in this study.

Data S3 (separate file). Cell type quantification data from experiments in this study.

SUPPLEMENTAL METHODS

cDNA preparation and RT-qPCR

To isolate total RNA, pooled organoids (10–20 organoids, from at least 6 wells per condition) were lysed in TRIzol Reagent (Invitrogen 15596018), followed by aqueous phase purification using the RNA Clean and Concentrator-5 kit (Zymo Research R1016) and DNAse digestion on column (Qiagen 79254). 500 ng total RNA were used for cDNA synthesis using the Maxima First Strand cDNA synthesis kit (Thermo Scientific K1672). qPCR was performed using the DyNAmo ColorFlash SYBR green qPCR kit (Thermo Scientific F416XL) on a CFX Connect Real-Time System (Bio-Rad). qPCR reactions were set up in technical duplicates and data from at least three biological replicates (different dams) were collected, except for experiments in Figure 3I where $n = 2$ dams. Ct values were normalized to the mouse *Actb* (beta actin) and *Maea* (Macrophage erythroblast attacher) genes (Shin et al., 2022). The qPCR primer sequences for *Actb*, *Maea*, *Ager* (Advanced glycosylation end product-specific receptor), *Sftpc* (Surfactant protein C), *Lamp3* (Lysosome associated membrane glycoprotein 3), *Lyz1*, *Lyz2* (Lysozyme 1 and 2), *Nfkbia*, *Nfkbib*, *Nfkbiz* (Nuclear factor of kappa light polypeptide gene enhancer in B cells inhibitor, alpha, beta, and zeta) are shown in Table S3. The Ct values are shown in Data S2.

Lung tissue immunostaining

Fetal mouse lungs were perfused with PBS and fixed overnight at 4°C in 4% PFA. After PBS washes, the lungs were brought in 10 and 30% sucrose in PBS for cryoprotection. After OCT embedding (Tissue-Tek 4583), 10 µm cryosections were cut from frozen tissue blocks using a Leica CM1950 cryotome.

Antibodies

Primary antibodies used are: anti-CDH1 (E-cadherin, 1:500, Santa Cruz sc-59778), anti-EMCN (Endomucin, 1:250, Santa Cruz sc-65495, R&D AF4666), anti-RAGE (Advanced glycation end product-specific receptor, 1:250, R&D MAB1179), anti-Pro-SFTPC (Pro-surfactant protein C, 1:500, Millipore AB3786), anti-CSPG4/NG2 (Chondroitin sulfate proteoglycan 4, 1:500, Millipore AB5320), anti-ACTA2-Cy3 (Alpha smooth muscle actin, 1:1000, Sigma C6198), anti-SPARCL1 (Secreted protein acidic and rich in cysteine-like protein 1, 1:250, R&D AF2836), anti-TLR4 (Toll-like receptor 4, 1:250, Proteintech 19811-1-AP), and anti-LAMP3 (Lysosome associated membrane glycoprotein 3, 1:250, Dendritics DDX0192).

Cell proliferation analysis

Terminal labeling of proliferating cells was performed by incubating organoids with 10 µM EdU for 12 h. After fixation, organoids were immunostained and EdU incorporation was revealed using the Click-iT EdU Cell Proliferation Kit (Thermo Scientific C10340). Between 38 and 67 branching structures per condition per replicate were blindly selected for imaging. A total of 465 (control) and 508 (SPARCL1) cells were identified on day 7. A total of 329 (control) and 247 (SPARCL1) cells were identified on day 8. Statistical significance was computed by Mann-Whitney U test.

Library preparation

For RNA-seq analysis, total RNA was isolated from control and SPARCL-treated organoids using the miRNeasy micro Kit (Qiagen) combined with on-column DNase digestion (RNase-Free DNase Set, Qiagen) to avoid contamination by genomic DNA. RNA and library preparation integrity were verified with LabChip Gx Touch 24 (Perkin Elmer). 2 µg of total RNA was used as input for VAHTS Stranded mRNAseq V6 Library preparation following manufacturer's protocol (Vazyme). Sequencing was performed on NextSeq2000 instrument (Illumina) with 1x72bp single end setup.

RNA-seq analysis

Trimmomatic version 0.39 was employed to trim reads after a quality drop below a mean of Q15 in a window of 5 nucleotides and keeping only filtered reads longer than 15 nucleotides (Bolger et al., 2014). Reads were aligned versus Ensembl mouse genome version mm39 (Ensembl release 109) with STAR 2.7.10a (Dobin et al., 2013). Alignments were filtered to remove: duplicates with Picard 3.0.0 (Picard: A set of tools (in Java) for working with next generation sequencing data in the BAM format; http://broadinstitute.github.io/picard/), multi-mapping, ribosomal, or mitochondrial reads. Gene counts were established with featureCounts 2.0.4 by aggregating reads overlapping exons on the correct strand excluding those overlapping multiple genes (Liao et al., 2014). The raw count matrix was normalized with DESeq2 version 1.36.0 (Love et al., 2014). Contrasts were created with DESeq2 based on the raw count matrix. Genes were classified as significantly differentially expressed at average count > 5, multiple testing adjusted *p*-value < 0.05, and -0.585 < log2FC > 0.585. The Ensemble annotation was enriched with UniProt data (Activities at the Universal Protein Resource (UniProt)).

GSEA analysis

For gene set enrichment analysis, we used the GSEA tool (https://www.gsea-msigdb.org/gsea/index.jsp) and the Msigdb mouse H collection. We ran 1000 permutations on the ranked bulk RNA dataset (*FC* x (log10(*pvalue*) x (-1))). We excluded gene sets larger than 800 genes and small than 10 genes. Plotting was performed by python matplotlib.pyplot.scatter function.

scRNA-seq analyses

We used the public dataset (Negretti et al., 2021) provided by the Sucre Lab for our analysis. For the analysis of the endothelial cells, we extracted cells from the time points E12, E15, E16, E18, P0, and P3. Normalization, PCA, dimensionality reduction and clustering was done using the Scanpy (Wolf et al., 2018) framework and custom scripts. Of note, we did not perform a batch correction on individual samples. We isolated cluster 1 (containing the majority of E16 cells) and cluster 3 (containing the majority of E18 cells) and utilized Scanpy's (*rank_genes_groups*) function to determine the differentially expressed genes between the two clusters.
For epithelial analysis, we excluded Ciliated, Secretory, and Neuroendocrine clusters from the analysis. To score for the SPARCL1-induced NF-κB signature genes, we used Scanpy's (*score_genes*) function. The resulting score was used to split cells into "NF-kB+" and "NF-kB-" subpopulations and plotting (Figures 4B, S4A, S4B, S4D). Trajectory and pseudo-time analysis in Figure S4C was performed using scFates (Faure et al., 2023) with (*method="ppt", Nodes=50, ppt_lambda=200, ppt_sigma=0.6*). The proportion analysis for both datasets was done

using the tool Scanpro (Alayoubi et al., 2024), utilizing real and pseudo-replicates. *p* values from Scanpro are from empirical Bayes moderated ANOVA test.

For Figures S4E-S4J, the raw data were downloaded from GEO: GSE158192. We only used control samples for our analysis (Aggregate12files_control) (Hassan and Chen, 2024). The subsequent analysis was carried out using the sc_framework environment (Schultheis et al. (2024), https://zenodo.org/doi/10.5281/zenodo.11065517). We sub-setted the dataset to only include time points E16.5, E18.5, P7 and 15wk. QC included doublet detection and filtering by Scrublet (Wolock et al., 2019). All cells with less than 200 and more than 4000 genes, as well as cells with more than 35% ribosomal gene counts were filtered out. Further, cells with a high mitochondrial content (>10%) were discarded. This initial cell filtering resulted in a dataset with 20374 cells and 18619 genes. Counts per cell were normalized to the median count over all cells and transformed into log space to stabilize variance. The cell cycle for each cell was predicted based on predefined marker gene lists.

For (sub)clustering of *Cdh1* lineage cells, we used principal component analysis (PCA) for dimensionality reduction and included the first 35 components. We then calculated a neighbor graph (15 neighbors) and used UMAP for two dimensional embedding. To annotate cells, we used the “lineage” information provided by the original publication (Hassan and Chen, 2024), which divided the cells into epithelial, immune, mesenchymal, and endothelial clusters based on the expression of *Cdh1*, *Ptprc*, *Col3a1*, and *Icam2* respectively. After lineage annotation, we isolated epithelial (*Cdh1*+) cells and ran PCA, UMAP embedding and clustering separately as described before. To remove possible contamination, we retained only cells that express *Nkx2-1* (marker for epithelial cells) and removed all cells expressing *Vim* (marker for mesenchymal cells). We further subsetted the dataset to AT1, AT2, Sox9 progenitors and prolif. AT2 cells using the labels from the original publication, resulting in a dataset with 3474 cells. We calculated the mean expression of SPARCL1-induced NF-κB signature genes (*Ccl2*, *Tnfaip2*, *Cxcl5*, *Cxcl1*, *Lcn2*, *Adora1*, *Slc5a1*, *Ccl20*, *Mab21l3*, *Vnn3*, *Tnfaip3*, *Chil1*, *Cxcl2*, *Steap4*, *Muc4*, *Fcer2a*, *Lyz1*, *Scd1*, *Nfkbiz*, *Col6a4*, *Parm1*, *Saa3*, *Cftr*, *Zc3h12a*, *C3*, *Cd83*) in each time point and plotted them in the UMAP embedding as well as a violin plot, comparing the mean expression in AT1 and AT2 cells.

Phosphatidylcholine uptake and acidic organelle staining

Assays for the uptake of neutral lipids and acidic organelle staining were performed as described in (Chiu et al., 2022). Briefly, organoids were cultured for 24 h in the presence of 1 μM β-BODIPY FL C12-HPC (Invitrogen D3792). After washing with culture medium, organoids were stained using 100 nM LysoTracker Deep Red (Invitrogen L12492) and Hoechst 33342 (Invitrogen H3570) for 30 min at room temperature immediately followed by imaging.

Transmission Electron Microscopy

The organoids were fixed in 2% PFA and 2.5% glutaraldehyde in 0.1 M sodium cacodylate buffer (pH 7.4) for 2 h at room temperature, and then stored at 4°C overnight. Samples were washed in 0.1 M sodium cacodylate buffer and postfixed in 2% (w/v) OsO_4, followed by *en bloc* staining with 2% uranyl acetate. Samples were dehydrated with a graded series of washes in ethanol, transferred to ethanol/Epon solutions, and eventually embedded in Epon. Ultra-thin sections (approximately 60 nm thick) obtained with a Leica UC7 microtome were collected on copper slot grids with a pioloform support film. Sections were post-stained with uranyl acetate and lead citrate. Sections were examined with a Jeol JEM-1400 Plus transmission electron microscope (Jeol, Japan), operated at an accelerating

voltage of 120 kV. Digital images were recorded with an EM-14800 Ruby Digital CCD camera unit (3296 px x 2472 px).

SUPPLEMENTAL REFERENCES

Alayoubi, Y., Bentsen, M., and Looso, M. (2024). Scanpro is a tool for robust proportion analysis of single-cell resolution data. Sci Rep *14*, 15581. https://doi.org/10.1038/s41598-024-66381-7.

Beauchemin, K.J., Wells, J.M., Kho, A.T., Philip, V.M., Kamir, D., Kohane, I.S., Graber, J.H., and Bult, C.J. (2016). Temporal dynamics of the developing lung transcriptome in three common inbred strains of laboratory mice reveals multiple stages of postnatal alveolar development. PeerJ *4*, e2318. https://doi.org/10.7717/peerj.2318.

Bolger, A.M., Lohse, M., and Usadel, B. (2014). Trimmomatic: a flexible trimmer for Illumina sequence data. Bioinformatics *30*, 2114–2120. https://doi.org/10.1093/bioinformatics/btu170.

Chiu, M.C., Li, C., Liu, X., Yu, Y., Huang, J., Wan, Z., Xiao, D., Chu, H., Cai, J.-P., Zhou, B., et al. (2022). A bipotential organoid model of respiratory epithelium recapitulates high infectivity of SARS-CoV-2 Omicron variant. Cell Discov *8*, 1–15. https://doi.org/10.1038/s41421-022-00422-1.

Daniel, E., Azizoglu, D.B., Ryan, A.R., Walji, T.A., Chaney, C.P., Sutton, G.I., Carroll, T.J., Marciano, D.K., and Cleaver, O. (2018). Spatiotemporal heterogeneity and patterning of developing renal blood vessels. Angiogenesis *21*, 617–634. https://doi.org/10.1007/s10456-018-9612-y.

Dobin, A., Davis, C.A., Schlesinger, F., Drenkow, J., Zaleski, C., Jha, S., Batut, P., Chaisson, M., and Gingeras, T.R. (2013). STAR: Ultrafast universal RNA-seq aligner. Bioinformatics *29*, 15–21. https://doi.org/10.1093/bioinformatics/bts635.

Faure, L., Soldatov, R., Kharchenko, P.V., and Adameyko, I. (2023). scFates: a scalable python package for advanced pseudotime and bifurcation analysis from single-cell data. Bioinformatics *39*, btac746. https://doi.org/10.1093/bioinformatics/btac746.

Gillich, A., Zhang, F., Farmer, C.G., Travaglini, K.J., Tan, S.Y., Gu, M., Zhou, B., Feinstein, J.A., Krasnow, M.A., and Metzger, R.J. (2020). Capillary cell-type specialization in the alveolus. Nature *586*, 785–789. https://doi.org/10.1038/s41586-020-2822-7.

Hassan, D., and Chen, J. (2024). CEBPA restricts alveolar type 2 cell plasticity during development and injury-repair. Nat Commun *15*, 4148. https://doi.org/10.1038/s41467-024-48632-3.

Liao, Y., Smyth, G.K., and Shi, W. (2014). featureCounts: an efficient general purpose program for assigning sequence reads to genomic features. Bioinformatics *30*, 923–930. https://doi.org/10.1093/bioinformatics/btt656.

Liu, B., Yi, D., Yu, Z., Pan, J., Ramirez, K., Li, S., Wang, T., Glembotski, C.C., Fallon, M.B., Oh, S.P., et al. (2022). TMEM100, a Lung-Specific Endothelium Gene. Arteriosclerosis, Thrombosis, and Vascular Biology *42*, 1495–1497. https://doi.org/10.1161/ATVBAHA.122.317683.

Love, M.I., Huber, W., and Anders, S. (2014). Moderated estimation of fold change and dispersion for RNA-seq data with DESeq2. Genome Biology *15*, 550. https://doi.org/10.1186/s13059-014-0550-8.

Negretti, N.M., Plosa, E.J., Benjamin, J.T., Schuler, B.A., Habermann, A.C., Jetter, C.S., Gulleman, P., Bunn, C., Hackett, A.N., Ransom, M., et al. (2021). A single-cell atlas of mouse lung development. Development *148*, dev199512. https://doi.org/10.1242/dev.199512.

Schultheis, H., Detleffsen, J., Wiegandt, R., Bentsen, M., Alayoubi, Y., Valente, G., Keßler, M.F., Heger, V., Khassafi, F., Talyan, S., et al. (2024). loosolab/SC-Framework: Version 0.7 (Zenodo).

Shin, H., Morty, R.E., Sucre, J.M., Negretti, N.M., Markmann, M., Hossain, H., Krauss-Etschmann, S., Dehmel, S., and Hilgendorff, A. (2022). Reference genes for the developing mouse lung under consideration of biological, technical and experimental confounders. Sci Rep *12*, 17679. https://doi.org/10.1038/s41598-022-19071-1.

Wolf, F.A., Angerer, P., and Theis, F.J. (2018). SCANPY: large-scale single-cell gene expression data analysis. Genome Biology *19*, 15. https://doi.org/10.1186/s13059-017-1382-0.

Wolock, S.L., Lopez, R., and Klein, A.M. (2019). Scrublet: Computational Identification of Cell Doublets in Single-Cell Transcriptomic Data. Cell Systems *8*, 281-291.e9. https://doi.org/10.1016/j.cels.2018.11.005.
